# Translational Profiling of Clock Cells Reveals Circadianly Synchronized Protein Synthesis

**DOI:** 10.1371/journal.pbio.1001703

**Published:** 2013-11-05

**Authors:** Yanmei Huang, Joshua A. Ainsley, Leon G. Reijmers, F. Rob Jackson

**Affiliations:** Department of Neuroscience, Sackler School of Biomedical Sciences, Tufts University School of Medicine, Boston, Massachusetts, United States of America; University of Geneva, Switzerland

## Abstract

Genome-wide studies of circadian transcription or mRNA translation have been hindered by the presence of heterogeneous cell populations in complex tissues such as the nervous system. We describe here the use of a *Drosophila* cell-specific translational profiling approach to document the rhythmic “translatome” of neural clock cells for the first time in any organism. Unexpectedly, translation of most clock-regulated transcripts—as assayed by mRNA ribosome association—occurs at one of two predominant circadian phases, midday or mid-night, times of behavioral quiescence; mRNAs encoding similar cellular functions are translated at the same time of day. Our analysis also indicates that fundamental cellular processes—metabolism, energy production, redox state (e.g., the thioredoxin system), cell growth, signaling and others—are rhythmically modulated within clock cells via synchronized protein synthesis. Our approach is validated by the identification of mRNAs known to exhibit circadian changes in abundance and the discovery of hundreds of novel mRNAs that show translational rhythms. This includes *Tdc*2, encoding a neurotransmitter synthetic enzyme, which we demonstrate is required within clock neurons for normal circadian locomotor activity.

## Abstract 2

This study describes, for the first time, the rhythmic translational program within circadian clock cells. The results indicate that most clock cell mRNAs are translated at low-energy times of either mid-day or mid-night, and also that related cellular functions are coordinately regulated by the synchronized translation of relevant mRNAs at the same time of day.

## Author Summary

The circadian clock controls daily rhythms in physiology and behavior via mechanisms that regulate gene expression. While numerous studies have examined the clock regulation of gene transcription and documented rhythms in mRNA abundance, less is known about how circadian changes in protein synthesis contribute to the orchestration of physiological and behavioral programs. Here we have monitored mRNA ribosomal association (as a proxy for translation) to globally examine the circadian timing of protein synthesis specifically within clock cells of *Drosophila*. The results reveal, for the first time in any organism, the complete circadian program of protein synthesis (the “circadian translatome”) within these cells. A novel finding is that most mRNAs within clock cells are translated at one of two predominant circadian phases—midday or mid-night—times of low energy expenditure. Our work also finds that many clock cell processes, including metabolism, redox state, signaling, neurotransmission, and even protein synthesis itself, are coordinately regulated such that mRNAs required for similar cellular functions are translated in synchrony at the same time of day.

## Introduction

Genetic studies carried out in several model systems have provided seminal knowledge about the biochemistry of the circadian molecular oscillator and the neural circuitry regulating circadian behavior. The best characterized circadian oscillators consist of transcriptional/translational feedback loops (TTFLs) [1], although nontranscriptional oscillators (NTOs) exist in organisms ranging from unicellulars to *Drosophila* and humans [2]–[4]. In *Drosophila* and mammals, a well-characterized TTFL oscillator consisting of several canonical clock genes regulates circadian behavioral rhythms (reviewed in [1]). Similarly, transcription of many (perhaps most) genes is orchestrated by the circadian clock, based on gene profiling studies carried out in *Drosophila*, mammals and plants. Only a few studies, however, have documented cell-type–specific transcriptional rhythms [5]–[7], due to methodological limitations. Most of those studies utilized Fluorescence-Activated Cell Sorting (FACS), the manual isolation of identified cells, or cell-specific transcriptional profiling techniques, but such methods are either not applicable to all cell populations or lack the sensitivity to detect the entire transcriptome; nor do they distinguish between ribosome-bound (i.e., translating) and soluble mRNA without the use of polyribosome isolation.

Drosophila is an excellent model for cell-type–specific profiling of clock cells because of its outstanding genetics and well-characterized circadian system. Studies have described the fly circadian molecular oscillator [8] and the circadian neuronal circuitry [9], revealing molecular and functional differences among groups of pacemaker neurons that mediate morning and evening bouts of activity or responses of the clock to environmental cues [5],[10]–[18]. To date, no study has documented genome-wide expression profiles for all clock cells of the fly head or the complete translatome of such cells. In this study, we describe use of the Translating Ribosome Affinity Purification (TRAP) method [19] to define the rhythmic translatome of circadian clock cells. Our results reveal a daily synchronization of protein synthesis and identify novel cycling mRNAs within clock cells that are required for diverse physiological processes.

## Results

### Implementation of TRAP for Studies of Circadian Biology

**Figure 1:**
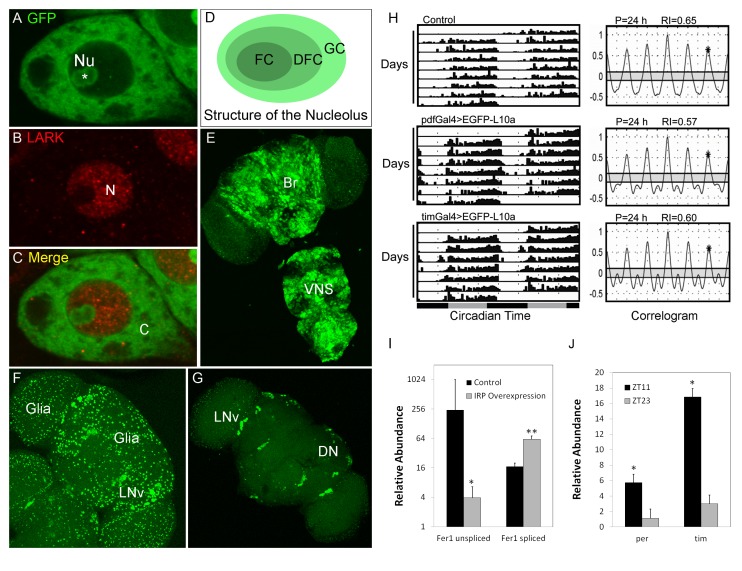
Expression of EGFP-L10a and assays of function in clock cells. (A–C) Expression of EGFP-L10a in a large neurosecretory cell. Nu, nucleolus; N, Nucleus; C, Cytoplasm. Staining for a nuclear protein called LARK (red signal) is used to identify the nucleus. (D) Schematic representation of the structure of the nucleolus. FC, Fibrillar Center; DFC, Dense Fibrillar Components; GC, Granular Components. GC is the location of ribosome assembly. (E) Expression pattern of EGFP-L10a in the brain and ventral ganglion using the *elav-Gal4* pan-neuronal driver. (F) Expression of EGFP-L10a in all clock cells driven by *tim-Gal4*. (G) Restricted expression of EGFP-L10a to clock neuron but not glia using a combination of *tim-Gal4* and *repo-Gal80*. (H) Expression of EGFP-L10a in clock cells does not disrupt normal circadian behavior. Left panels shows representative free-running actograms of control flies and flies expressing EGFP-L10a in either PDF neurons (using *pdf-Gal4*) or all clock cells (using *tim-Gal4*). Right panels show the corresponding correlograms. (I) TRAP is capable of detecting changes in mRNA translation, as assayed by changes in the translational status of Ferritin 1 Heavy Chain Homolog (Fer1HCH) mRNA in response to overexpression of the Iron Regulatory Protein (IRP). Control, *w^1118^; act5C-Gal4/tub-Gal80^ts^; UAS-EGFP-L10a/+*. IRP overexpression, *w^1118^; act5C-Gal4/tub-Gal80^ts^; UAS-EGFP-L10a/UAS-IRP*. (J) Circadian changes in the translation of period (*per*) and timeless (*tim*) mRNAs. Genotype of the flies assayed, *elav-Gal4; UAS-EGFP-L10a/+*. Error bar represents standard error of the mean (SEM). *p<0.01; **p<0.001 (Student's *t* test).

Previous studies have shown that TRAP reflects the translational status of mRNAs in a manner similar to that of conventional polyribosomal analysis [19]. In addition, a recent study in *Drosophila* indicates that an EGFP-L10a fusion incorporates into polysomes and can be employed for cell-specific translational profiling [20]. To employ TRAP in our studies, we generated *Drosophila* strains carrying a *UAS-EGFP-L10a* transgene insertion (see Materials and Methods). Using a pan-neuronal driver (*elav-Gal4*), we found that the EGFP-L10a fusion has a cytoplasmic/nucleolar pattern of localization in neurons (Figure 1A–C), consistent with incorporation into ribosomes. Indeed, the ring shape pattern in nucleoli (seen in the nucleus of Figure 1A) likely results from expression in the Granular Component (GC, Figure 1D), a structure within which ribosomal proteins assemble into functional ribosomes. As expected, EGFP-L10a was localized in all neurons of the adult nervous system (Figure 1E). In contrast, the *tim-uas-Gal4* driver results in expression within the cytoplasm of clock neurons and glia of the nervous system (Figure 1F) or only clock neurons when combined with *repo-Gal80* (Figure 1G), which inhibits expression in all glial cells. A different GFP–*Drosophila* ribosomal protein fusion (L11) has an identical intracellular localization pattern [21]. In addition, it has recently been shown that our EGFP-L10a fusion localizes to branch points of neuronal dendrites, consistent with incorporation into ribosomes that mediate local protein synthesis [22]. Collectively, these pieces of evidence indicate that EGFP-L10a incorporates into functional ribosomes.

We examined circadian locomotor activity of flies overexpressing the *UAS-EGFP-L10a* transgene in clock cells (Pigment Dispersing Factor, PDF, or Timeless, TIM) to determine whether there were adverse effects on behavior. As shown in Figure 1H, these files have normal behavioral rhythmicity, indicating that EGFP-L10a does not act in a dominant negative manner even at high levels [Average periods (P) and Rhythmicity Indices (RI) were 23.7±0.08/0.57±0.02, 24.0±0.03/0.55±0.01, and 24.3±0.14/0.50±0.03 for control, *pdf-Gal4>UAS-EGFP-L10a*, and *tim-uas-Gal4>UAS-EGFP-L10a* flies; *n* = 17–30]. Thus, the presence of GFP-tagged ribosomes in clock cells does not affect their function.

### TRAP Can Detect Changes in Translational Status

We optimized TRAP methods for use with *Drosophila* and demonstrated that significant amounts of RNA could be immunoprecipitated from head tissues of flies expressing *UAS-EGFP-L10a* under control of the pan-neural *elav-Gal4* or clock cell *tim-uas-Gal4* driver (see Materials and Methods). Prior to pursuing genome-wide studies, we wished to determine if our *Drosophila* TRAP methods could detect *bona fide* changes in translational status. To ask this question, we employed overexpression of Iron Regulatory Protein (IRP), which is known to repress translation of an unspliced form of ferritin (*fer*) mRNA by inhibiting binding of the small ribosomal subunit to the message. We generated *act5C-Gal4/tub-Gal80^ts^, UAS-EGFP-L10a/UAS-IRP* flies in order to be able to activate expression of the TRAP and IRP transgenes conditionally during larval development (by raising temperature to inactivate Gal80^ts^, an inhibitor of Gal4). Larvae of this genotype and controls (*act5C-Gal4/tub-Gal80^ts^; UAS-EGFP-L10a*) were exposed to 30°C to activate expression of *UAS-EGFP-L10a* in both genotypes and additionally *UAS-IRP* in the experimental class. Early pupae were collected for both genotypes and subjected to TRAP coupled with Q-RT-PCR to quantify ribosome-associated *fer* mRNA (relative to control *Rp49* mRNA). Similar to previous studies in *Drosophila* that employed polysome gradient analysis [23], we observed IRP-induced translational repression of an unspliced but not a spliced form of *fer* (Figure 1I). Indeed, translation of spliced *fer* was enhanced slightly by IRP overexpression, similar to that observed from the analysis of a high molecular weight polysome fraction in the previous study [23]. This result shows feasibility for the use of TRAP in *Drosophila* to detect changes in translational status.

To determine if our methods were able to detect rhythmic changes in the ribosomal association of cycling transcripts, we examined the *period (per)* and *timeless (tim)* clock mRNAs. TRAP methods were employed to immunopurify RNA from head tissues of *elav-Gal4/UAS-EGFP-L10a* flies two times of day (ZT11 and ZT23, the times of high and low *per/tim* RNA abundance, respectively). Extracted RNA was then subjected to Q-RT-PCR, using gene-specific primers, to detect the clock mRNAs. Figure 1J shows that the abundances of ribosome-bound *tim* and *per* clock mRNAs are significantly higher at ZT11 than at ZT23. This result is consistent with the known rhythmic profile of *tim* and *per* RNA abundances at the two time points (higher at ZT11) and the expected translational status of the mRNA at the two times of day. We emphasize that Figure 1J shows differences in ribosome association of the clock RNAs, not simply the previously documented RNA abundance for *per* and *tim*. In addition, we note that the temporal resolution of our measurements does not exclude translational regulation of *per* mRNA, which has been suggested in certain studies [24]–[26]. Nonetheless, these results demonstrate that TRAP methods are capable of detecting diurnal changes in the translational status of specific mRNAs.

### Clock Cell-Specific Expression Profiling Efficiently Detects Circadianly Translated RNAs

Using the newly developed methods, we performed TRAP on head tissue lysates of *tim-uas-Gal4; UAS-EGFP-L10a* flies that were collected at 4-h intervals during the first two days of constant darkness (DD) following entrainment to LD 12∶12. Such flies express the EGFP-L10a fusion in all clock cells of the head, including the ∼150 pacemaker neurons, photoreceptors, and glia. RNA was extracted from affinity-purified samples and used to generate libraries representing all ribosome-associated transcripts (see Materials and Methods). TRAP libraries corresponding to six different times of the circadian cycle (CT0, 4, 8, 12, 16, and 20) were independently constructed for DD1 and DD2 (see details in Materials and Methods). Libraries were sequenced, using a multiplexing strategy, to produce single end, 100 base sequencing reads; these were mapped to the *Drosophila* reference genome and analyzed as described in Materials and Methods.

**Figure 2:**
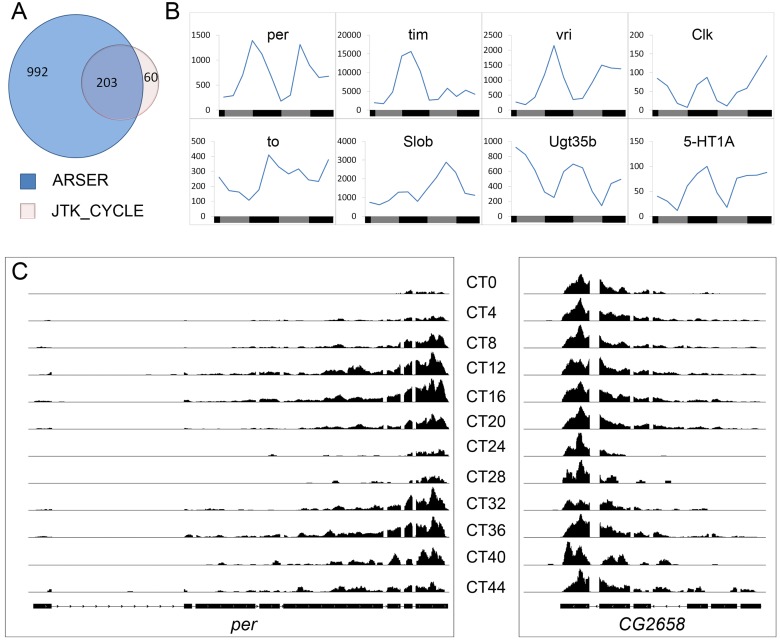
Identification of mRNAs displaying a circadian translational rhythm in clock cells. (A) Number of rhythmically translated genes identified by two different programs: JTK_CYCLE and ARSER. (B) Translational profile of known cycling genes. The *y*-axis represents normalized read counts. (C) Quantification of sequence reads aligned to the period (*per*) gene and a nearby nonrhythmic gene (CG2658) across the time-series.

We employed two recently developed programs, JTK_CYCLE and ARSER [27],[28], to compare their usefulness for detecting circadian rhythms in the ribosome association of mRNAs. Using criteria and statistical cutoffs described in the Materials and Methods section, 1,195 and 263 translationally cycling mRNAs were detected by the ARSER and JTK_CYCLE programs, respectively. Interestingly, the majority of the cycling mRNAs (203 out of 263) detected by JTK_CYCLE were also detected by the ARSER program (Figure 2A), indicating consistency of the two analyses. Figure S1 shows robust cycling for eight mRNAs out of the 60 identified by JTK_CYCLE but not ARSER. Thus, JTK_CYCLE may identify cycling mRNAs not detected by ARSER. Table S4 lists the 1,255 mRNAs that were identified as exhibiting significant translational cycling by either program (mRNAs identified by both programs are indicated in bold). The False Discovery Rate (FDR) calculated by the ARSER program at the relevant *p* value was 0.148, indicating that approximately 186 mRNAs are false positives. This FDR is quite low relative to other recent genome-wide studies of cycling mRNAs [29]–[31]. We did not compute an FDR for the JTK_CYCLE program, because 203/263 mRNAs identified by JTK_CYCLE are included in the ARSER dataset, and therefore the latter dataset represents a good approximation of FDR for our analyses. Based on the ARSER analysis, we estimate that approximately 1,069 of these mRNAs show circadian changes in translation in clock cells of the adult head, representing about 10% of all analyzed genes in the genome. This large number of cycling mRNAs is consistent with recent studies utilizing manual dissection approaches to perform cell-specific transcriptional profiling of the *Drosophila* PDF clock neurons [5],[10]. Cell-specific profiling methods may identify a larger number of cycling *Drosophila* mRNAs, relative to previous studies, due to a more homogeneous starting cell population (i.e., clock cells).

### Known Cycling mRNAs Exhibit Translational Rhythms

We examined a number of mRNAs in our datasets that had previously been shown to exhibit abundance rhythms to assess the quality of our datasets. These include both clock and clock-regulated mRNAs (*per*, *tim*, *vri*, *clk*, *to*, *fer2*, *slob*, *ugt35b*, *5-HT1A*, *bw*, *Ir*, and *WupA*). All showed translational rhythmicity (Figure 2B) with an expected phase, although the *tim* rhythm damped on DD2. Figure 2C, for example, shows robust rhythmicity in the sequence reads for *per* and lack of rhythmicity for a nearby gene. Our analysis also revealed translational cycling for many other genes that express rhythmic mRNAs. For example, our list of mRNAs includes 49 of 420 mRNAs showing circadian abundance rhythms identified in five previous microarray-based studies (see Introduction). This comparison does not include a recent study that identified 2,751 cycling mRNAs in hand-dissected PDF neurons [10]; our results include 172 of those mRNAs (see Table S4). Interestingly, *Ugt35b* mRNA, one of several encoding fly glucuronosyltransferase activity, was previously shown to exhibit transcriptional cycling in head tissues but not in PDF neurons [10]. Given that we employed a clock cell *tim-Gal4* driver in our TRAP studies, we suggest that *Ugt35b* cycles in other clock cells of the head.

We conducted TRAP combined with quantitative PCR for *Ugt35b*, *tim*, and 18 novel cycling mRNAs (not previously found to show abundance rhythms in head tissues) to verify results obtained by RNA-seq. As expected, *Ugt35b* and *tim* exhibited rhythmicity, presumably a consequence of their mRNA abundance rhythms. Of the novel mRNAs, 15/18 showed rhythmic changes in translation, with a profile very similar to that observed with RNA-seq analysis (Figure S2). We further analyzed cycling of a number of these mRNAs in the *per^0^* mutant, which lacks a functional clock, during the first day of constant darkness (DD1). We found that rhythmic expression of these mRNAs was abolished in the *per^0^* mutant, confirming their circadian clock regulation (Figure S3).

### Translational Profiling Reveals Circadianly Synchronized Protein Synthesis

**Figure 3:**
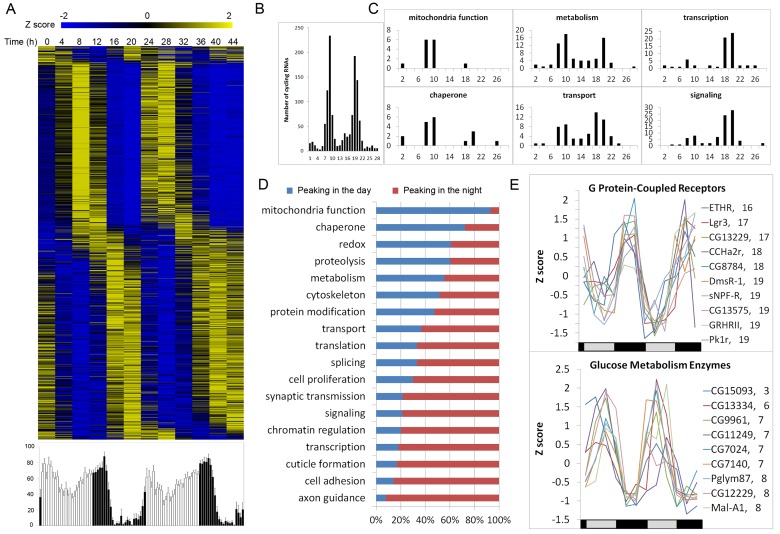
TRAP identifies two major phases of rhythmic translation. (A, Upper) A heat map showing the relative level of translation during DD days 1–2 for each of the 1,255 genes. Genes are arranged vertically according to their phases. (A, Lower) Population plot of free-running activity (DD days 1–2) for the fly strain used to generate the translational profiles (vertical axis, activity level; horizontal axis, time of day). *n* = 17, error bars are SEM. (B) Phase distributions of ribosome association for all cycling RNAs. (C) Phase distributions of cycling RNAs relevant for several different cellular processes. Horizontal axes show phase; vertical axes indicate the number of RNAs. (D) Day or night distribution for major biological processes. (E) Translational profiles of mRNAs representing two functional groups: G protein–coupled receptors (upper panel) and glucose metabolic enzymes (lower panel).

Previous genome-wide studies showed that peaks of mRNA abundance occur at many different circadian phases (see Figure S4). In contrast, our profiling of the clock cell translatome revealed a striking feature of circadianly regulated protein synthesis. We found that peak translation for most of the 1,255 mRNAs identified in our study occurs predominantly during two circadian phases: midday or mid-night (Figure 3A–C; Figure S4). These are times of relative behavioral quiescence and just prior to initiation of locomotor activity bouts (Figure 3A, lower panel). Thus, protein synthesis may be confined to times of day that require reduced metabolic expenditure and/or are just prior to initiation of behavioral activities. A further analysis revealed surprisingly synchronized translation of mRNAs required for the same cellular process: translation is predominantly unimodal (with a peak during the day or night) or biomodal, depending on the process (Figure 3C). This bias in the timing of translation was true of many other cellular processes (Figure 3D). For example, of the 10 enzymes involved in glucose metabolism in our list of cycling RNAs, nine are translated during the day. In contrast, all 10 GPCRs in our list are translated during the night (Figure 3E).

Of note, mRNAs encoding a number of translational initiation factors (eIF4E isoforms) exhibit cycling with a phase that corresponds to the daytime peak of circadian translation (Figure S5). Thus, circadian translation of these eIFs may contribute to a broad clock regulation of protein synthesis. In contrast, the major initiation factor, eIF4E-1, does not exhibit translational cycling, suggesting that it does not participate in circadian regulation (Figure S5). Consistent with previous results indicating that ribosome biogenesis is regulated in a circadian manner [32], 20 mRNAs encoding ribosomal proteins, translation initiation factors, or other translational regulatory components show translational rhythmicity (Table S4).

### Translational Regulation Contributes to Circadian Gene Expression

The synchronized rhythmic expression profiles identified by our cell-specific profiling approach may result from a clock regulation of translation or mRNA abundance. To ask whether changes in translational status contribute to the synchronization of gene expression in clock cells, we carried out additional studies, using TRAP/RNA-seq methods.

We reasoned that total RNA isolated from whole heads contains mRNAs from both clock and nonclock cells. Thus, if a gene is robustly expressed in nonclock cells, the abundance profile obtained from whole head total RNA will not represent its expression profile in clock cells. However, for mRNAs predominantly expressed in clock cells (such as *per*, *tim*, and others), assays of total head RNA will reflect clock cell expression. Such an mRNA ought to show enrichment in a TRAP sample from *tim-uas-gal4>EGFP-L10a* heads relative to total RNA from the starting lysate, and the circadian expression profile, when assayed from total RNA, should approximate the profile in clock cells. Thus, if such an mRNA shows a rhythm by TRAP but not in total RNA, then it is likely to be regulated at the translational level.

**Figure 4:**
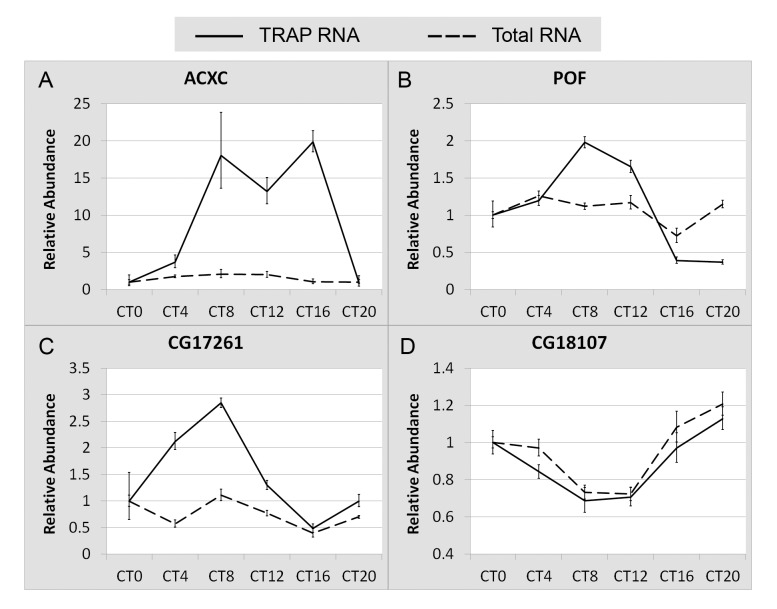
Comparison of abundance and ribosome-association profiles for several mRNAs. (A–C) Examples of mRNAs that show constant abundance but rhythms in ribosome association. (D) An example of an mRNA showing both abundance and ribosome association rhythms. RNA abundances were normalized to that of Rp49 for each time point. Abundance is expressed relative to that of the first time point (CT0), which was designated a value of 1. Negative and positive error bars show the range of possible relative values calculated based on the SEM of the Ct values obtained in the Q-PCR experiments. Each data point represents a sample size of 6 (3 biological replicates, each with 2 technical replicates).

To identify mRNAs enriched in clock cells, we created new genome-wide libraries for TRAP and total RNA samples from head tissues of *tim-uas-gal4>EGFP-L10a*–expressing flies. These were sequenced to identify mRNAs that are substantially enriched by TRAP relative to total RNA—that is, enriched in clock cells. We identified many that show an enrichment within clock cells similar to or greater than that observed for *tim* mRNA. Forty-nine of them are present in our previous list of cycling mRNAs. We chose 12 cycling mRNAs from the enriched list and examined their expression profiles in total RNA versus TRAP RNA samples using Q-PCR methods. Of the 12 mRNAs tested, three did not show cycling similar to that detected by RNA-seq analysis (25%, and the same as we reported for another set of RNAs; Figure S2); thus, these three were not examined further. Of the remaining nine mRNAs, which showed cycling by Q-PCR similar to that detected by RNA-seq, three of them exhibited constant abundance in total RNA but showed circadian cycling in ribosome association, indicating that they are likely regulated at the translational level. Figure 4 shows cycling profiles for these three mRNAs and a fourth mRNA showing both abundance and ribosome-association rhythms (Figure 4D). Thus, for certain mRNAs, there is good evidence for a clock regulation of translation.

### Broad Circadian Regulation of Clock Cell Physiology

**Figure 5:**
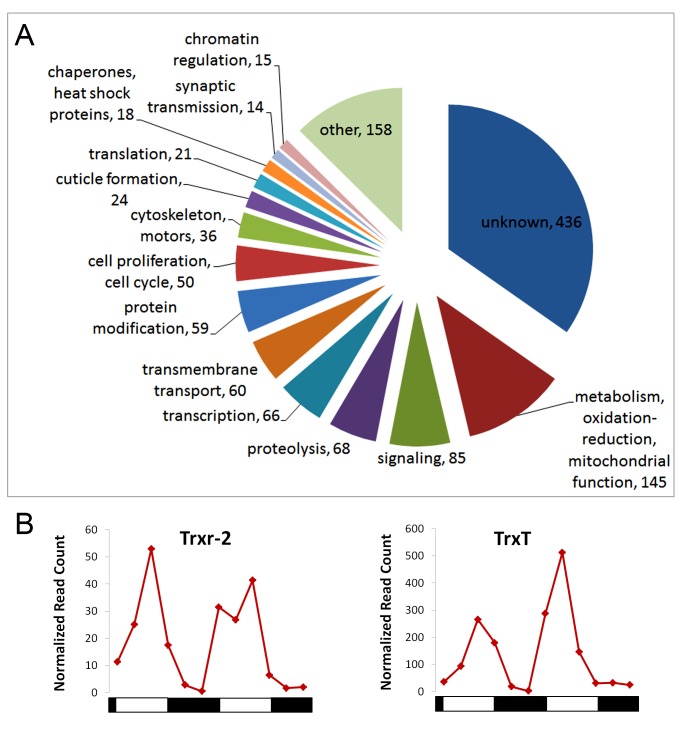
Biological processes represented by the rhythmically translated mRNAs. (A) Pie chart showing different represented processes. The number of mRNAs belonging to each category is shown next to each slice of the pie. (B) Translational profile of thioredoxin system mRNAs.

We manually annotated the proteins encoded by the 1,255 cycling RNAs using information obtained from Flybase and classified them by biological process (Figure 5A). Of the annotated genes, the most represented functional class is metabolism/energy production, including NAD-dependent processes and oxidation-reduction reactions. This class includes 85 genes involved in intermediary metabolism, 14 genes with mitochondrial functions, and 46 genes that regulate oxidation-reduction processes. These results are consistent with *Drosophila* and mouse circadian transcriptional profiling studies that identified a large subset of metabolic genes [33],[34]. Another overrepresented group is signaling (including both intracellular pathways and intercellular signaling mechanisms). Interestingly, 44 members of the signaling class belong to the G Protein signaling family, represented by many G Protein Coupled Receptors (GPCRs) and GTPases.

### Rhythmic Translational Regulation of the NADP+/NADPH Ratio and Cellular Redox State

Several particularly interesting cycling mRNAs encode proteins that potentially modulate the NADP+/NADPH ratio or are known components of the cellular redox (thioredoxin) system. Examples include the CG3483 and CG7755 genes, both predicted to encode isocitrate dehydrogenase-like proteins. While at least one isocitrate dehydrogenase (*IDH*) is a component of the mitochondrial citric acid cycle, others have a cytoplasmic localization, producing αketoglutarate with a conversion of NADP+ to NADPH [35]. We also found that the mRNA encoding Glutathione Transferase E10 (GstE10), which utilizes the redox regulator glutathione in detoxification reactions, exhibits a translational rhythm (Table S4). Interestingly, it was recently shown that glutathione and a different *Gst* mRNA (*GstD1*) show circadian changes in abundance in *Drosophila* head tissues [36], suggesting a complex regulation of redox state.

Components of the thioredoxin (TRX) system, a general regulator of cellular redox state, are also under circadian control. *Thioredoxin T* (*TrxT*) and *Thioredoxin reductase* (*Trxr-2*) mRNAs show robust circadian changes in translation, with peaks in the late subjective day (Figure 5B). This circadian translation may reflect an underlying transcriptional control as both *TrxT* and *Trxr-2* show mRNA abundance rhythms in *Drosophila* head tissues (Figure S6). Of interest, it was previously suggested that *TrxT* showed an mRNA abundance rhythm within the *Drosophila* PDF clock neurons, but this was based only on examination of two circadian times in a screen for cycling mRNAs [10]. Thioredoxin reductases are known to catalyze reduction of thioredoxin, in the process converting NADPH to NADP+ [37], an important regulator of cellular redox. In addition to these TRX system genes, *Grx-1*, a glutaredoxin also involved with cell redox state homeostasis, shows circadian translational cycling (Table S4). Rhythmicity in cellular redox state is significant as it regulates many biochemical processes including circadian transcription factors (see Discussion).

### Circadian Translational Regulation of Nervous System Functions

Previous studies have indicated that synaptic vesicle cycling mechanisms are important within clock neurons [38] and glial cells [39] for circadian oscillator or output functions. Similarly, there are reciprocal interactions between the oscillator and neuronal membrane events, including ion channel activity, that are critical for timekeeping in Drosophila and mammals [6],[40],[41]. It is of interest that we identified mRNAs encoding at least 20 ion channels or channel regulatory proteins that exhibit rhythms in ribosome association. These include *cac* (Ca^2+^ channel), *Ir* (K+ channel), *SK* (K+ channel), *Slob* (K+ channel regulator), and *inaF-B* (Trp channel regulator), although *Ir* showed significant rhythmicity only during day 1 of DD. Interestingly, however, *Ir* was identified in a previous study as a rhythmic mRNA within PDF neurons that is important for oscillator function [6]. Likewise, a number of mRNAs encoding vesicle trafficking or release proteins, including *exo70*, *syn*, and *unc-104*, exhibited rhythmicity in our experiments.

We note that at least two potential brain glial mRNAs were revealed in our study: CG9977 and CG6218. CG9977 encodes adenosylhomocysteinase activity, whereas CG6218 encodes an ATPase. Both were identified in a previous microarray-based screen for *Drosophila* mRNAs enriched in glial cells [42], and are known to be expressed in the adult brain according to FlyAtlas [43]. The CG9977 enzymatic activity converts S-adenosyl-L-homocysteine to L-homocysteine and adenosine, the latter a known mammalian gliotransmitter [44]. As the *tim-uas-gal4* driver is expressed in neurons and glia (including astrocytes) with PER-based oscillators, CG9977 and CG6218 may be expressed in the latter cell type.

### *Tdc2*, a Rhythmically Translated mRNA, Is Expressed in PDF Neurons and Required for Circadian Locomotor Activity

**Figure 6:**
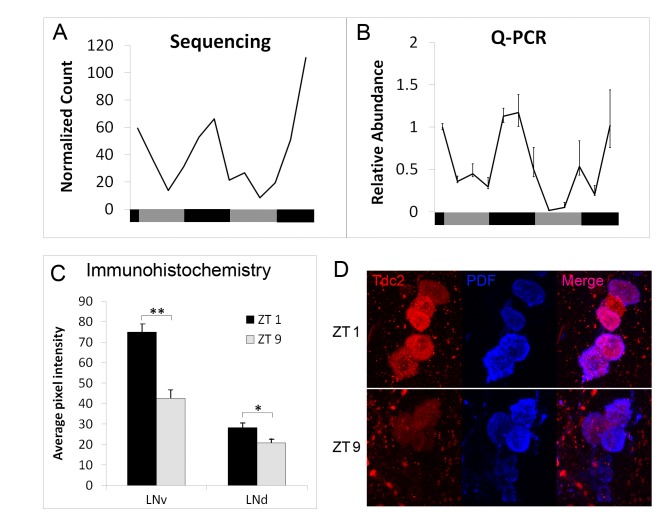
TDC2 protein shows circadian changes in the PDF-positive large ventral lateral neurons (l-LNvs) and dorsal lateral neurons (LNds). (A–B) Translational profile of *Tdc2* revealed by RNA sequencing (A) and Q-PCR (B). In the Q-PCR graph, the level of mRNA expression for the first time point (CT0) serves as a reference, and is thus designated a value of 1. RNA expression levels at other time points are plotted relative to the value at CT0. Negative and positive error bars show the range of possible relative values calculated based on the SEM of the Ct values obtained in the Q-PCR experiments. *n*≥4 for all time points. (C) Abundance of TDC2 protein in the l-LNvs and LNds at two different times of the circadian cycle, using immunohistochemical methods. (D) Sample images showing differential expression of TDC2 in l-LNvs (red channel) at ZT1 and ZT9. Quantification of average pixel intensities is described in the Materials and Methods section. For LNvs, 10 pairs of brain hemispheres were compared between ZT1 and ZT9. For LNds, nine pairs of brain hemispheres were compared between ZT1 and ZT9. *p<0.01; **p<1.5E-05 based on paired Student's *t* test.

*Tdc2* encodes the neurally expressed isoform of tyrosine decarboxylase, which converts tyrosine to tyramine, the latter compound acting as a substrate for octopamine synthesis. In *Drosophila*, both tyramine and octopamine serve as neurotransmitters, regulating diverse functions including adult locomotion, male aggression, male courtship, drug sensitivity, ovulation, circadian activity rhythms, and appetitive memory formation [45]–[49]. Therefore, it is of interest that *Tdc2* mRNA exhibits circadian translational rhythms in clock cells (Figure 6A; Table S4). We verified the circadian translation of *Tdc2* using the TRAP technique coupled with Q-RT-PCR (Figure 6B), and showed that this rhythm is abolished in *per^0^* flies (Figure S3). Using an anti-TDC2 antibody, we further verified that expression of the TDC2 protein exhibits the predicted circadian changes in two major groups of clock neurons, the l-LNvs and the LNds (Figure 6C and D).

We used two different strategies to characterize the expression pattern of *Tdc2* in the adult brain, in particular in various groups of clock cells. First, we characterized the expression pattern of a *Tdc2-Gal4* transgene [50] and its co-localization with PERIOD protein. We found that a *UAS-GFP* reporter, driven by *Tdc2-Gal4*, was expressed in multiple regions of the fly brain, as expected. However, the only clock cells showing GFP fluorescence (identifiable by PER expression) were the ventral lateral (LNv) PDF neurons of the brain (Figure S7A), which are critical for circadian behavior [9]. Next, using anti-TDC2 antibody, we localized the TDC2 protein in flies expressing a membrane-bound GFP (mCD8-GFP) in all clock cells (driven by *tim-uas-gal4*). As expected, we found that there is a strong immunoreactive signal for TDC2 in many nonclock neurons. Within the clock neuronal population, we detected TDC2 immunoreactivity in all l-LNvs (Figure S7B, a–d), s-LNvs (a–d), and LNds (i–k), as well as a few cells in the DN1 region (l–n). Finally, a comparison of TDC2 immunoreactivity and *Tdc2-gal4* driven mCD8-GFP expression found that *Tdc2-gal4* does not express in all TDC2 immunoreactive cells (unpublished data), indicating that the *Tdc2-gal4* transgene does not reflect the complete expression pattern of the *Tdc2* gene. These results suggest that rhythmic production of TDC2 in various clock neurons, and a consequent rhythm in release of tyramine and/or octopamine from these cells, may be required for normal circadian behavior.

**Figure 7:**
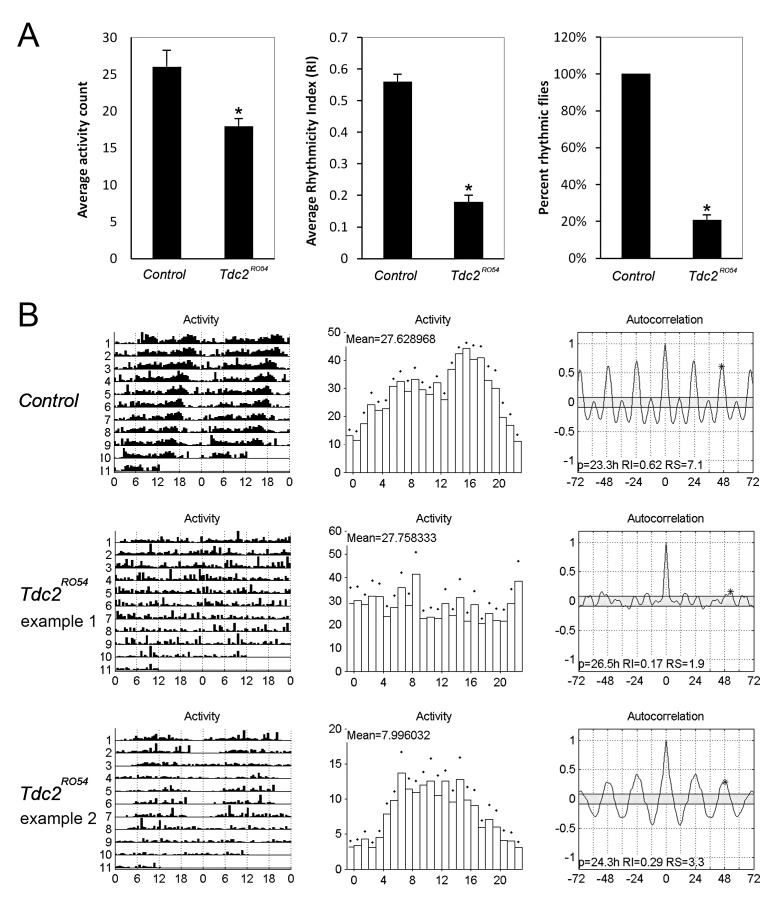
Mutation of the *Tdc2* gene results in decreased activity and circadian arrhythmicity for adult locomotor activity. (A) Quantification of average activity level, average rhythmicity index (RI), and percent of rhythmic flies in wild-type and *Tdc2^RO54^* populations. *n* = 25 for control; *n* = 29 for *Tdc2^RO54^*. Error bars represent SEM. *p<0.0001. (B) Representative actograms, mean activity, and correlograms for control flies and the *Tdc2^RO54^* mutant.

To assess the role of *Tdc2* in circadian behavior, we analyzed locomotor activity of the *Tdc2^RO54^* mutant, which carries a point mutation that abolishes the enzymatic activity of TDC2 [50]. Consistent with previous reports [45], we found that *Tdc2^RO54^* mutants displayed decreased activity (Figure 7A). In addition, however, the mutant population exhibited decreased rhythmicity. The average Rhythmicity Index (RI) for *Tdc2^RO54^* was 0.18±0.02 compared to 0.56±0.02 for control flies, and indeed only 20±3% of the mutant population displayed significant free running rhythms, whereas the control population was 100% rhythmic (Figure 7A). We note that decreased activity does not result in arrhythmicity, as there was no correlation between activity level and rhythmic locomotor activity (Figure 7B). These observations indicate that octopamine and/or tyramine are required for normal circadian behavior (see Discussion).

**Figure 8:**
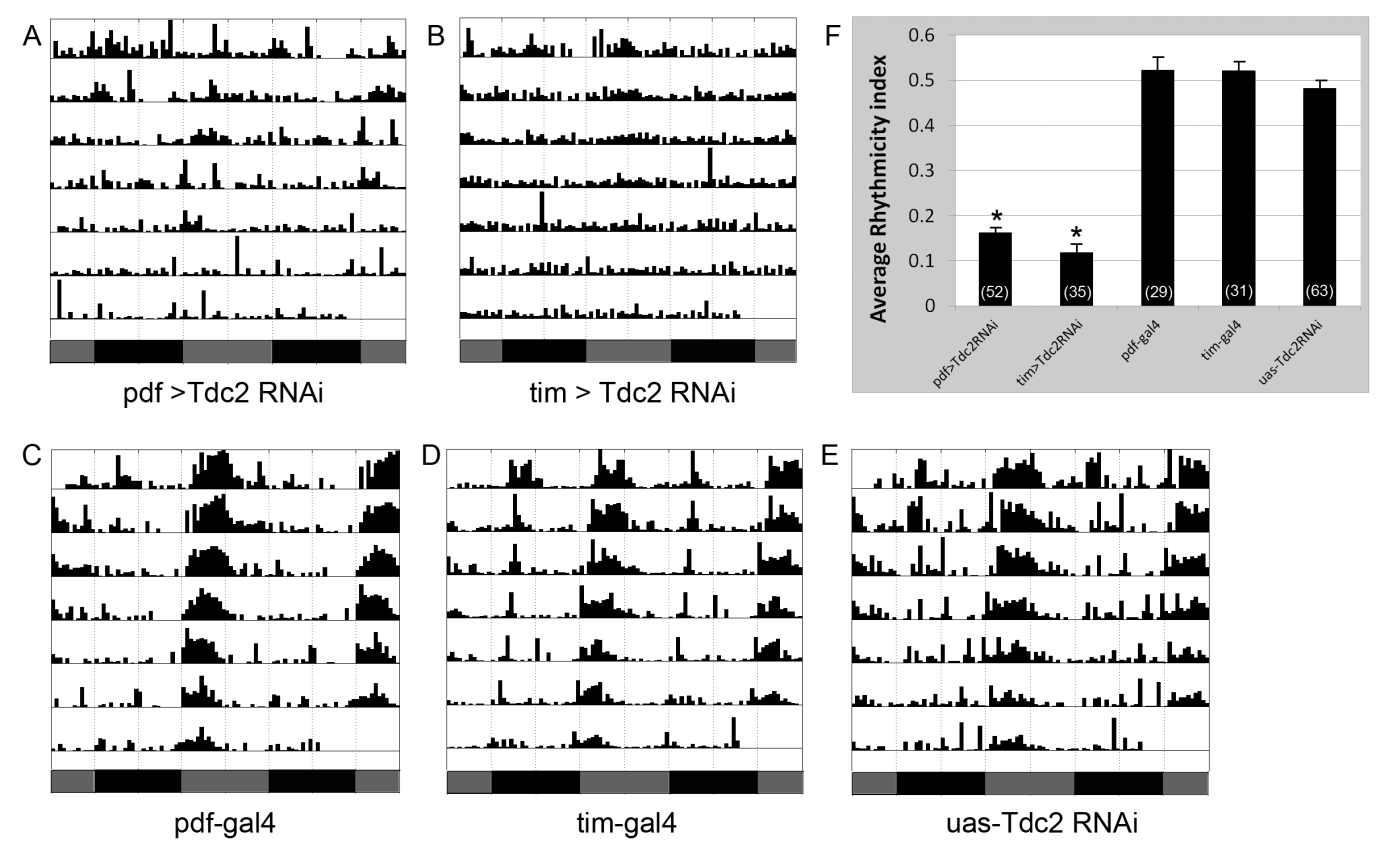
Knockdown of *Tdc2* in clock neurons results in circadian behavioral arrhythmicity. (A–E) Representative actograms showing free-running locomotor activity of flies with a Tdc2 knockdown in PDF neurons (A) or all clock neurons (B), as well as relevant control files (C–E). (F) Quantification of the average rhythmicity index (RI) for various genotypes. Number of flies tested is indicated on the histograms. *p<1.4E-30 for comparison with the control groups based on Student's *t* test.

To ask whether the observed arrythmicity of the *Tdc2* null mutant results from loss of *Tdc2* function in clock cells, we examine circadian behavior in flies with a *Tdc2* knockdown specifically in clock cells. We found that populations of flies expressing *Tdc2* RNAi, driven by either *pdf-gal4* or *tim-uas-gal4*, were 75% arrhythmic and had low average Rhythmicity Indices (Figure 8A,B,F), whereas control flies were normally rhythmic (Figure 8C–F) Thus, tdc2 is required within clock neurons for normal locomotor activity rhythms.

## Discussion

### Synchronized Translational Rhythmicity

This study is the first to profile the circadian translatome of a defined cell population in a complex tissue. In contrast to previous studies showing that mRNA abundance rhythms peak at multiple circadian phases (Figure S4), our results indicate that translation of most rhythmic transcripts within clock cells is restricted to two major phases—midday and mid-night. Furthermore, we provide evidence that circadian regulation of either mRNA abundance or protein synthesis (depending on the mRNA) contributes to this synchronization. We speculate that protein synthesis may occur predominantly at circadian phases that are associated with reduced metabolic expenditure. In *Drosophila*, such times coincide with behavioral quiescence, just prior to initiation of locomotor activity bouts (Figure 3). The synchronized translation of functionally related mRNAs (Figure 3B–D) suggests a clock-orchestrated sequential activation of biological processes; these results reinforce the concept that fundamental cellular processes are under circadian control within clock cells.

Two significant technical improvements enabled the discovery of synchronized translation. First, our analysis was restricted to circadian clock cells, circumventing the problem of profiling a mixed population, in which some cells express a rhythmic mRNA, whereas others express the same mRNA constitutively (thus masking rhythmicity). In addition, different cell types may express out-of-phase rhythmic mRNAs, also masking a rhythm in a mixed cell population. Second, our technique analyzes ribosome association rather than steady-state mRNA abundance, representing a more direct assessment of protein expression. Although it is not currently technically feasible to directly compare transcriptional and translational rhythms in the same cell types, our results indicate that translational regulatory mechanisms contribute to synchronized protein synthesis. Consistent with this idea, we have shown that mRNAs encoding relevant translational regulatory factors are rhythmically expressed. These include translation initiation factors, ribosomal proteins, and enzymes involved in rRNA and tRNA synthesis (Table S4). In mammals, ribosome biogenesis is known to be regulated by the circadian clock [32]. Thus, it is possible that the circadian clock regulates translation of many mRNAs, including those relevant for clock function [25],[26] by controlling availability of the translational apparatus.

### Rhythms in Cellular Redox State

We document rhythmic translation of mRNAs within clock cells that is relevant for diverse biochemical and behavioral functions. A particularly interesting class includes factors important for cell redox homeostasis (*CG3483*, *CG7755*, *TrxT*, and *Trxr-2*), as it has been demonstrated that a clock control of redox state drives rhythms in the excitability of suprachiasmatic nuclei (SCN) neurons [51]. Furthermore, there is redox control of many cellular factors, including enzymes, receptors, cytokines, growth factors, and transcription factors. Thioredoxin, for example, regulates NFkB activity [52], which is known to be under circadian control [53]. NADP(H) and NAD(H), the reduced forms of these cofactors, stimulate DNA binding of the CLOCK/BMAL1 and NPAS2/BMAL1 transcriptional heterodimers, which are critical components of the mammalian circadian clock [54]. Circadian translational regulation of cellular redox may be important for rhythmicity of clock components and clock outputs as well as metabolic feedback to the clock [33],[55].

The rhythm in *TrxT* translation may also function in another important circadian output. It has recently been demonstrated that there is circadian control of peroxiredoxin (PRX) protein oxidation state in organisms ranging from unicellulars to humans, and that this rhythm is regulated by an uncharacterized NTO (reviewed in [1]). Significantly, oxidized PRX multimers serve as cellular chaperones and cell cycle modulators. Thioredoxin (TRX) mediates reduction of oxidized PRX molecules to complete the PRX catalytic cycle [1], and thus rhythmic *TrxT* may contribute to circadian changes in PRX oxidation state. Of relevance, mRNAs encoding other chaperones are also rhythmically translated (Table S4).

### Evidence for a Novel Neurotransmitter in PDF Neurons

Rhythmic factors important for neurotransmission were also identified by our analysis. Among them, *Tdc2*—encoding the synthetic enzyme for tyramine and octopamine—is rhythmically expressed in clock neurons and localized to the PDF cell population. Rhythmic release of these transmitters from PDF or other clock neurons may contribute to the temporal coordination of the clock cell circuitry, similar to the role of PDF [56]. Alternatively, rhythmic release of octopamine and/or tyramine may regulate downstream neurons that drive locomotor activity rhythms.

Of note, previous studies have suggested a clock control of tyramine synthesis, showing that there was decreased tyrosine decarboxylase activity in the brains of *per* clock mutants [57]. In addition, mutants of several clock genes, including *per*, *clock*, *cycle*, and *doubletime*, were found to be required for normal cocaine sensitization, a process depending on induction of tyrosine decarboxylase activity and production of tyramine [58]. Expression of *Tdc2* in clock neurons is consistent with a role for tyramine, and perhaps octopamine, in this process.

*Tdc2* was not described as a cycling mRNA in several previous genome-wide circadian expression studies [59]–[63] that utilized whole fly heads as a starting material. Based on the expression pattern of *Tdc2*—that is, broad and strong expression in a large number of neurons including only certain clock neurons —it seems likely that the cycling of *Tdc2* eluded detection in previous studies because of the presence of other TDC2-containing neurons in which the transcript does not exhibit rhythms in abundance. Indeed, *Tdc2* was included in a long list of mRNAs (2,751) showing enrichment in the large LNv clock neurons at one time of day (ZT12) in a recent study that utilized manual dissection procedures to profile PDF neurons [10]. Our detection of rhythmic *Tdc2* translation and confirmation of its role in maintaining circadian locomotor activity rhythms clearly demonstrates the advantage of a cell-type–specific approach in genome-wide studies of gene expression.

## Materials and Methods

### *Drosophila* Strains, Rearing Conditions, and Genetic Crosses

For translational profiling of flies with a normal circadian clock, males of a homozygous w^1118^; tim-uas-gal4 stock were crossed to virgin females of a homozygous *w^1118^; UAS-EGFP-mL10a* stock. F1 progeny carrying both the UAS and Gal4 transgenes (and expressing EGFP-tagged ribosomes in all clock cells) were collected and used for TRAP experiments. To profile a clock mutant, females from a homozygous *per^0^ w^1118^; UAS-EGFP-L10a* stock were crossed to a Gal4 strain and only male flies from the F1 progeny were used in the TRAP experiments. All flies were reared in a lighting schedule consisting of 12 h of light and 12 h of dark (LD 12∶12) at 25°C and 60% humidity. The tdc2^RO54^ strain and its isogenic parental strain were gifts from Dr. Jay Hirsh of University of Virginia. *UAS-Tdc2* RNAi flies were obtained from the VDRC stock center (stock numbers 10687R-1 and 10687R-3).

### Construction of a *UAS-EGFP-L10a* Transgene and Production of Transgenic *Drosophila*

The *UAS-EGFP-L10a* transgene was generated by cloning the coding sequence of the *EGFP-L10a* fusion protein (provided by Nat Heintz) into the pUAST vector. We chose to use the mouse L10a ribosomal protein (mL10a) because it is virtually the same as fly L10a (identical in size and ∼90% similar)—not surprising for a ribosomal subunit—and it has been shown to work well for the TRAP method. The cloning service was provided by Entelechon (Regensburg, Germany), and the resulting *UAS-EGFP-L10a* plasmid was verified by sequencing. The *UAS-EGFP-L10a* plasmid was purified using a Qiagen Maxi-prep kit and then used to generate transgenic flies (Genetic Services, Cambridge, MA). Genomic insertions were mapped to chromosomes using standard segregation analysis procedures.

### Affinity Purification of Ribosomes and Isolation of Ribosome-Bound mRNAs

Adult flies were collected in 50 ml conical tubes at desired time points and flash frozen in liquid nitrogen. Fly heads were collected by vigorously shaking frozen flies and passing them through geological sieves according to standard procedures. Approximately 200 heads were employed for each affinity purification experiment. Frozen heads were homogenized in a buffer containing 20 mM HEPES-KOH (pH 7.4), 150 mM KCl, 5 mM MgCl_2_, 0.5 mM DTT, 100 µg/ml Cycloheximide, and 2 U/ml SUPERase (Life Technologies) and centrifuged at 20,000× *g* for 15 min to obtain cleared lysate. After adding DHPC and Igepal CA-630 to a final concentration of 30 mM and 1%, respectively, the lysates were incubated on ice for 5 min and centrifuged at 20,000× *g* again for 15 min. After centrifugation, the supernatant was applied to magnetic beads coated with a purified high-affinity anti-EGFP antibody (prepared using the Dyabeads Antibody Couple Kit from Invitrogen) and incubated at 4°C with end-to-end rotation for 1 h to allow binding of EGFP-tagged ribosome to the antibodies. Following incubation, samples were washed with a buffer containing 20 mM HEPES-KOH (pH 7.4), 150 mM KCl, 5 mM MgCl_2_, 0.5 mM DTT, 100 µg/ml Cycloheximide, and 1% Igepal CA-630 for five times at room temperature. RNA was extracted from the beads using the TRIzol reagent (Life Technologies). Quality and quantity of the isolated RNAs were analyzed using a Bioanalyzer (Agilent).

Using these methods, we affinity purified RNA-containing ribosomes from head tissues of adult flies expressing *UAS-EGFP-L10a* in all neurons or clock cells. Similar to published studies [19], we optimized homogenization procedures for *Drosophila* head tissues, included magnesium and cycloheximide in the lysis buffer to preserve polysomes, inhibited RNAase activity, and employed a purified, high-affinity anti-GFP antibody for ribosome precipitation. In those experiments, the *UAS-EGFP-L10a* transgene was expressed in all neurons or all clock cells using, respectively, the *elav-Gal4* or *tim-uas-Gal4* drivers. In three pilot experiments—two using *elav-Gal4* and one using *tim-uas-Gal4*—we obtained a total of 305–544 ng RNA from head tissues of 200 *UAS-EGFP-L10a*–expressing flies, whereas there were negligible amounts (50–100-fold less) of precipitated RNA in control samples (*elav-Gal4* or *tim-uas-Gal4* alone) (Figure S8). Nearly as much RNA was precipitated using the *tim-uas-Gal4* driver as with *elav-Gal*, and we attribute this result to the strength of the *tim-uas-Gal4* driver and the observation that it is expressed in all clock neurons including photoreceptors and thousands of glial cells. With expression of *UAS-EGFP-L10a* in only the clock neuron population (∼150 neurons, some of which can be seen in Figure 1G), we were able to immunopurify 44 ng of RNA from 200 fly heads—10-fold more than control precipitations—indicating good sensitivity for our methods. Expression of a different ribosomal protein fusion, GFP-*Drosophila* L11 [21], can also be employed for TRAP analysis; we immunopurified 118 ng of ribosome-bound RNA from *elav-Gal4/UAS-GFP-L11* head tissues starting with 150 flies (unpublished data).

### RNA-Seq Library Construction and Sequencing

We employed standard Illumina protocols and reagents (the TruSeq RNA sample preparation kit) for RNA-seq library construction. RNAs extracted from the immunoprecipitation contain a mixture of mRNAs, ribosomal RNAs, and other small RNAs that are involved in translation, such as tRNAs. Using the TruSeq RNA kit, mRNAs were isolated using poly-dT coupled magnetic beads and fragmented by addition of divalent cations at 94°C. Cleaved mRNAs were then reverse transcribed into cDNA using random primers, and cDNA was subjected to second strand synthesis using DNA polymerase I and RNaseH. DNAs were end repaired, “A” tailed, and then ligated to Illumina sequencing adaptors prior to enrichment by PCR to create a library. Sequencing of libraries was accomplished using an Illumina HiSeq 2000 in the Tufts Medical School Molecular Core Facility. Sequence reads were obtained and their quality analyzed using the quality control metrics provided by the FastQC pipeline (http://www.bioinformatics.babraham.ac.uk/projects/fastqc/). We obtained, on average, 21 million high-quality 100-b reads for each of the 24 samples (after removing low-quality reads), and an average of 82% of the high-quality reads could be mapped to the *Drosophila* 5.22 reference genome (Table S1) using Tophat (v 2.0.0) and Bowtie2 (v 2.0.0.5) [64],[65]. The reads represent approximately ∼12,000 genes that are expressed in clock cells of the *Drosophila* head.

### Analysis of RNA-Seq Data

After mapping with Tophat and Bowtie, we counted the number of reads aligning to individual annotated genes in the *Drosophila* genome using HTseq-count (EMBL). Using these methods, there was good agreement between the two biological replicates for each time point in the DD1 and DD2 datasets. The correlation coefficient (r) of the two replicates was greater than 0.9 for all time points (Table S2, representative scatter plots of two replicates are shown in Figure S9). Next, we conducted a preliminary assessment of each of the four individual datasets (DD1, replicate 1 and 2 and DD2, replicate 1 and 2) by calculating the “best cosine correlation” for all genes including 10 genes in our datasets that are known to show transcriptional cycling from previous studies (Table S3). The “best cosine correlation” is obtained by calculating the correlation coefficient (r) between read counts of the six time points and corresponding six values on one of 48 cosine curves each with 0.5 h difference in phase, and selecting the highest r value from the 48 comparisons. We found that 10 “control” RNAs had high r values in the two DD1 datasets and DD2 dataset 1. However, poor correlation coefficients were observed for DD2 dataset 2, and thus this dataset was not used in our subsequent analyses. Given the good correlation between DD1 datasets 1 and 2, we pooled reads from these two replicates to generate one set of combined expression values. For DD2, we employed only dataset 1 in the analysis. As a consequence of improved sequencing technology, samples of the DD2 dataset 2 contained roughly the same number of reads as the combined DD1 datasets 1 and 2. Thus, the total number of reads analyzed for each sample was similar across all time points of DD1 and DD2—on average ∼32 million reads per sample. The resulting datasets (six time points for both DD1 and DD2) were quantile normalized to control for variation among experiments.

### Identification of Genes and Transcripts Showing a Circadian Expression Pattern

Relative sequence read coverage at different circadian time points, quantified using HTseq-count and quantile normalized, were used to construct a time-lapse expression series and analyzed using two different programs, ARSER and JTK_CYCLE [27],[28], to identify the presence of circadian periodicity. ARSER was developed by Yang and Su [27], and it analyzes circadian expression data by harmonic regression based on autoregressive spectral estimation; JTK_CYCLE was developed by Hughes et al. [28], and uses a nonparametric algorithm to detect rhythmic components in genome scale datasets. Results obtained from the two different analyses were filtered in several ways to obtain the final set of cycling genes: (1) we required the average raw read counts across the 12 time points to be at least 20; (2) we required a “cycling amplitude,” defined as ½ (maximum expression value – minimum expression value)/median expression value, of at least 0.5; and (3) for results with the ARSER program, *p*<0.021 was considered statistically significant, whereas for the JTK_CYCLE program, *p*<0.015 was used as a cutoff. As the two programs appear to have different sensitivities in detecting circadian genes, different cutoff *p* values were chosen for them in order to include the majority of known clock genes. We think the use of this biological criterion to determine a statistical cutoff is reasonable for this type of analysis.

### Identification of Genes Predominantly Expressed in Clock Cells

One-week-old adult flies expressing EGFP-mL10a in all clock cells—that is, carrying one copy each of *tim-uas-gal4* and *UAS-EGFP-L10a*—were entrained to a LD 12∶12 cycle for 3 d at 25°C and flash frozen in liquid nitrogen at ZT8 on the 4^th^ day. Three sets of samples, each containing about 200 flies, were collected. Head collection, homogenization, TRAP, and RNA isolation were carried out as described above in “Affinity Purification of Ribosomes and Isolation of Ribosome-Bound mRNAs.” Before the immunoprecipitation step, 1/10 of the tissue lysate was set aside for extraction of total RNA. RNAs were isolated from the TRAP immunoprecipitates (referred to as “TRAP RNA”) as well as from the input whole head lysates (referred to as “total RNA”). Equal amounts (300 ng) of TRAP RNA and total RNA were used to construct RNA-seq libraries. For each of the three sets of fly heads, one TRAP RNA library and one total RNA library were constructed. RNA-seq library construction, sequencing, and mapping were conducted as described above. Sequence read counts were obtained using HTSeq (EMBL) with BDGP5, Ensembl release 68 for gene coordinates. Normalized sequence read counts were used to test for differential expression between the TRAP RNA samples and whole head total RNA samples. Differential expression was determined using the DESeq package for R [66]. Genes that showed significantly increased abundance in the TRAP RNA samples were considered to be enriched in clock cells.

### Antibodies and Immunohistochemistry

Adult or larval brain and ventral ganglion were dissected in PBS (137 mM NaCl, 2.7 mM KCl, 8 mM Na_2_HPO_4_ • 2 H_2_O, 2 mM KH_2_PO_4_, pH 7.4) under a dissecting microscope and fixed in 4% paraformaldehyde. After fixation, tissues were washed three times with PBST (PBS with 0.1% Triton-X-100), blocked with 5% Normal Goat Serum (NGS) in PBST for 3 h, and incubated with primary antibody solution in PBST with 2% NGS at 4°C overnight. Anti-PER, anti-LARK, anti-TDC2, and anti-PDF primary antibodies were diluted 1∶15,000, 1∶2,000, 1∶300, and 1∶20, respectively. The primary antibody solution was removed the next day and tissues were washed five times in PBST and incubated with fluorescence-conjugated secondary antibody for 3 h (Cy3 conjugated goat anti-rabbit secondary antibody from Jackson ImmunoResearch for PER, LARK, and TDC2; Alexa Fluor 488 or Alexa Fluor 647 conjugated goat anti-mouse secondary antibody from Invitrogen for PDF). Following incubation with secondary antibody, tissues was washed five times in PBST and mounted on slides in VECTASHIELD mounting media (Vector Lab).

### Confocal Image Analysis and Quantification

Florescence microscopy of brains was conducted using either a Leica SP2 confocal microscope at the Tufts Center for Neuroscience Research (CNR) Imaging Core or a Leica SP8 confocal microscope at the Enhanced Neuroimaging Core of the Harvard NeuroDiscovery Center. GFP, Cy3, and Alexa Fluor 647 were excited using laser light of 488 nm, 561 nm, and 647 nm, respectively. Fluorescence excitation and image acquisition in the three different channels were performed in a sequential manner to avoid signal bleed-through between channels. One-micron optical sections were acquired in the vicinity of the LNv and LNd neurons using a 63× oil objective. Brain specimens collected from ZT1 and ZT9 were imaged in an alternating order so that every ZT1 image was paired with a ZT9 image and paired *t* tests were used in the final statistical analyses of image quantification. Such analyses minimize random variation due to fluctuation in laser power. To quantify TDC2 immunoreactivity in l-LNv and LNd, Regions of Interest (ROIs) were manually selected to include all l-LNv cells or all LNd cells based on PDF immunoreactivity (for l-LNv) or expression of *tim-uas-gal4*–driven mCD8-GFP in the appropriate region (for LNd). A custom Image J program was used to calculate the average pixel intensity across the entire stack within the ROI for all pixels that had an intensity value greater than that of a manually selected background region.

### Primers and Q-RT-PCR

Quantitative real-time PCR was conducted on a Stratagene Mx3000P or Mx4000 QPCR system using SYBR Green Real-time PCR Master Mix (Applied Biosystems). Primer sequences are listed in Table S5. Primers were tested to be sure a single product was amplified with the expected melting temperature. A primer pair for an abundant noncycling gene, Rp49, was used in all samples to serve as an internal control for the amount of starting material. The relative abundance of a gene of interest was calculated based on the difference between the Ct value of the specific primer pair and that of the Rp49 primer pair.

## Supporting Information

Figure S1 **Translational profiles of a number of genes identified by the JTK_CYCLE but not the ARSER program.**
(TIF)

Figure S2 **Comparison of RNA Sequencing and Q-PCRs result for 20 candidate cycling mRNAs.** Two panels are shown side-by-side for each mRNA containing the sequencing (left) and Q-PCR (right) results. In the Q-PCR graphs, mRNA abundance at the first time point (CT0) serves as a reference, and is thus designated a value of 1. Abundances at other time points are plotted relative to the value at CT0. Negative and positive error bars show the range of possible relative values calculated based on the SEM of the Ct values obtained in the Q-PCR experiments. *n*≥4 for all time points.
(TIF)

Figure S3 **Q-PCR analyses for six rhythmic mRNAs in wild-type and the** ***per^0^*** **mutant during the first day of DD.** Relative abundances were calculated based on comparison to that of a noncycling gene, Rp49. Negative and positive error bars show the range of possible relative values calculated based on the SEM of the Ct values obtained in the actual Q-PCR experiments. *n*≥4 for all mRNAs analyzed.
(TIF)

Figure S4 **Phase comparisons of translation (this study) and mRNA abundance rhythms documented in several previous studies.** Cycling mRNAs are arranged along the *x*-axis according to their phases, shown on the *y*-axis.
(TIF)

Figure S5 **Rhythmic translation of the *eIF-4E* mRNAs.** For each mRNA, translational level at each time point is normalized to the average translation across the time series.
(TIF)

Figure S6 **Q-PCR analyses of transcript abundance for *Trxr-2* and *TrxT* in total RNA samples collected at two different time points: CT8 and CT20.** *n* = 4 for all time points. Error bars represent SEM. *p<0.001 (Student's *t* test).
(TIF)

Figure S7 ***Tdc2*** **mRNA and protein are expressed in several groups of clock neurons including the PDF positive large and small ventral lateral neurons (LNvs), the dorsal lateral neurons (LNds), and dorsal neurons (DNs).** (A) Expression of *Tdc2-gal4* in the PDF neurons. Green, expression of mCD8-GFP driven by *Tdc2-gal4*; blue, PDF neuropeptide detected by anti-PDF antibody; red, PER protein detected by anti-PER antibody; arrow head, large LNvs; arrow, small LNvs; asterisk, PDF-negative small LNv. (B) Expression of TDC2 protein in LNvs (e–h), LNds (i–k), and DNs (l–m). Green, expression of mCD8-GFP driven by *tim-uas-gal4* (for marking all clock cells); blue, PDF neuropeptide detected by anti-PDF antibody; red, TDC2 protein detected by anti-TDC2 antibody; arrowhead, large LNvs; arrow, small LNvs.
(TIF)

Figure S8 **Using the TRAP technique, RNAs can be isolated from flies expressing EGFP-L10a but not from control flies without the transgene.** Note the difference in the scale of the *y*-axis.
(TIF)

Figure S9 **Scatter plots of read counts for all genes in two independent biological samples (sample 1, vertical axis; sample 2, horizontal axis) for all time points analyzed.**
(TIF)

Table S1 **RNA-seq statistics for all samples.**
(DOCX)

Table S2 **Correlation coefficients for two independent samples for each circadian time point.**
(DOCX)

Table S3 **Correlation coefficient of the circadian expression profiles of known clock mRNAss and those predicted by a standard cosine function.**
(DOCX)

Table S4 **All identified cycling mRNAs.**
(DOCX)

Table S5 **Primers used in the Q-RT-PCR experiments.**
(DOCX)

## Abbreviations

EGFP: enhanced green fluorescent protein
eIF4E: eukaryotic translation initiation factor 4E
GPCR: G protein-coupled receptor
L10a: large ribosomal protein 10a
L11: large ribosomal protein 11
LNd: dorsal lateral clock neurons
LNv: ventral lateral clock neurons
NTO: nontranscriptional oscillator
PDF: pigment dispersing factor
Per: Period
Tdc2: tyrosine decarboxylase 2
Tim: Timeless
TTFL: transcriptional/translational feedback loop
TRAP: Translating Ribosome Affinity Purification
TRX: thioredoxin
