# Supplementary figures and images for: Translational Profiling of Clock Cells Reveals Circadianly Synchronized Protein Synthesis
Source: PLoS Biol. 2013 Nov 5;11(11):e1001703. doi: 10.1371/journal.pbio.1001703 (PMC3864454; doi:10.1371/journal.pbio.1001703)

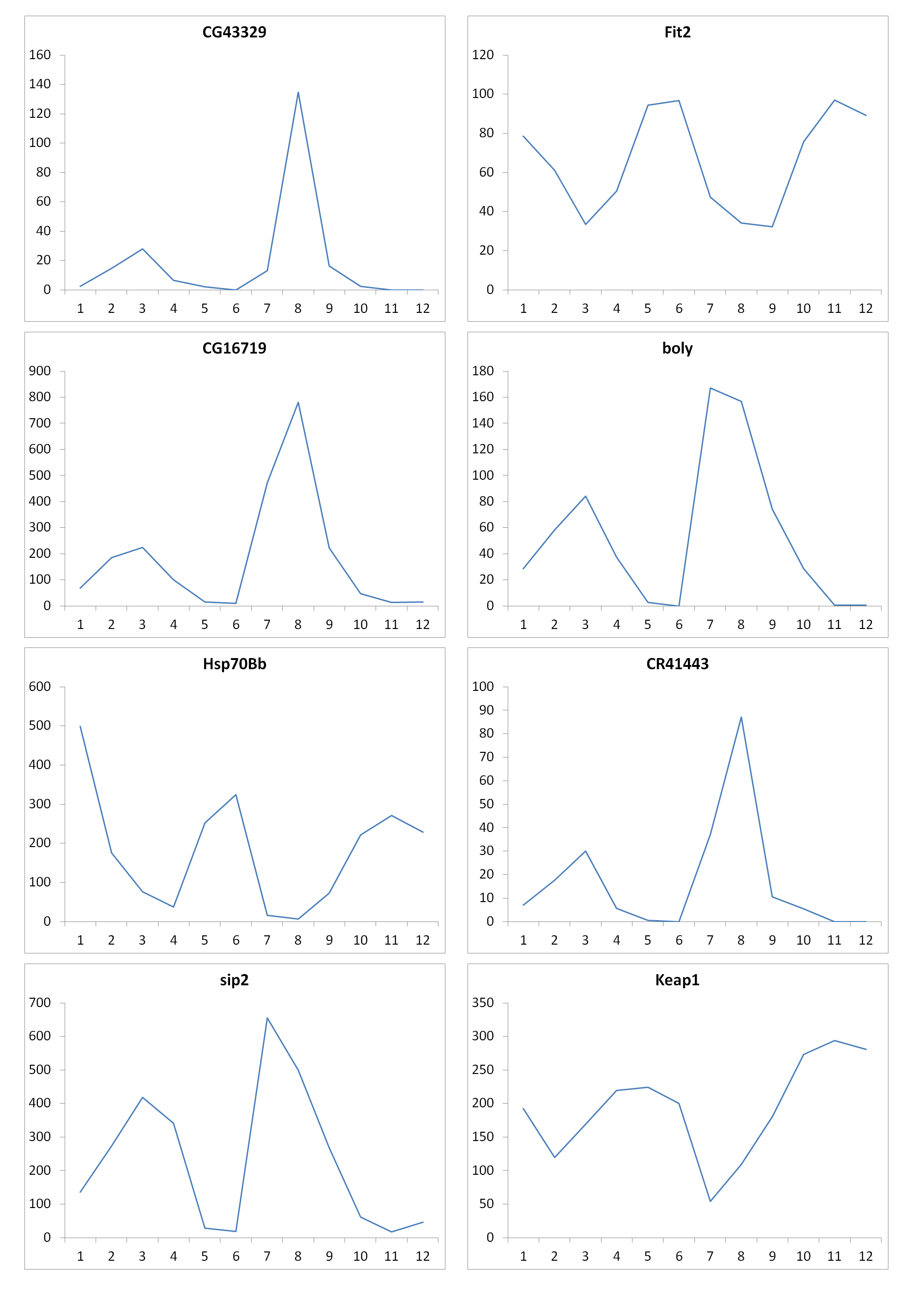

Supplement: Figure S1 — Translational profiles of a number of genes identified by the JTK_CYCLE but not the ARSER program. (TIF) [file pbio.1001703.s001.tif]

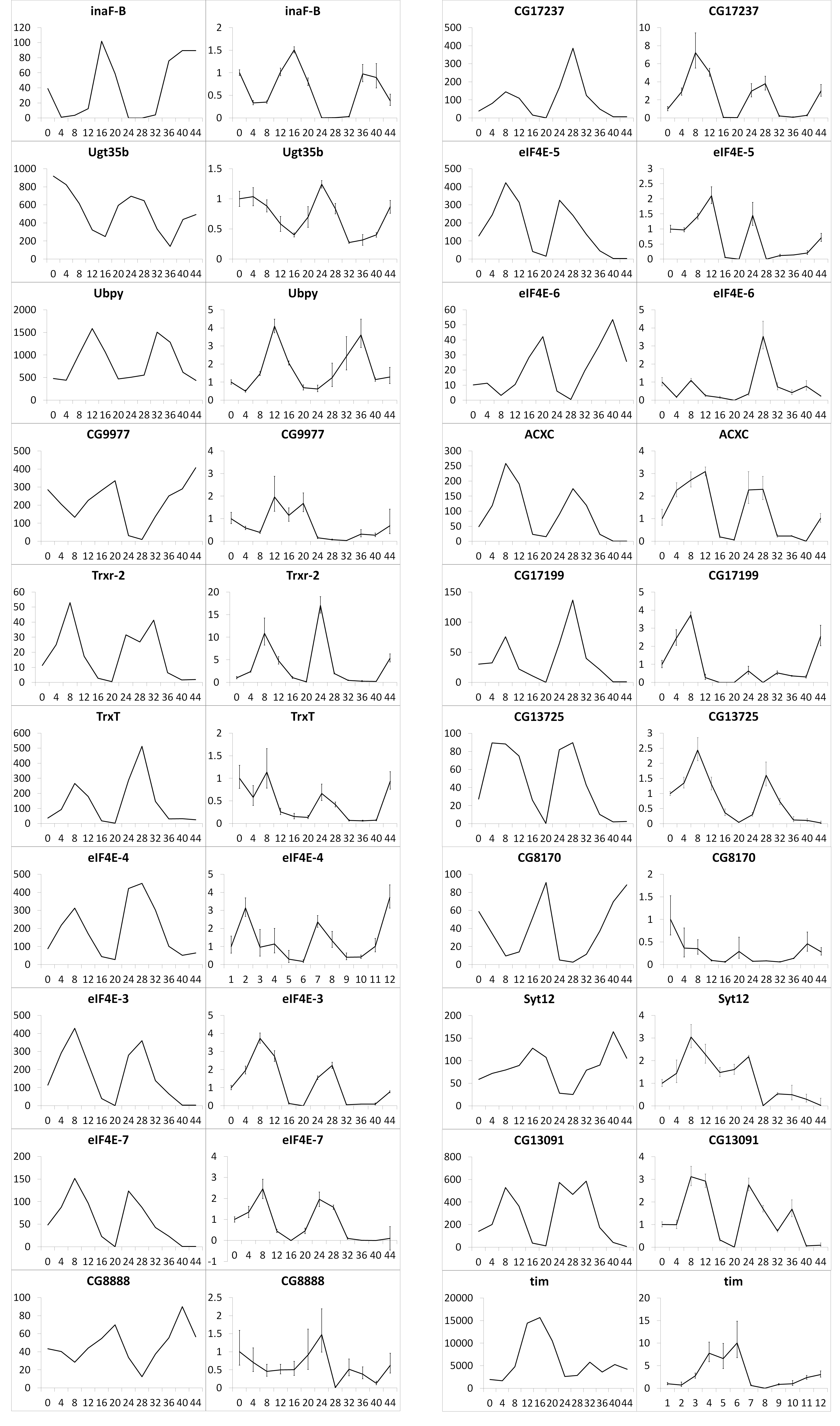

Supplement: Figure S2 — Comparison of RNA Sequencing and Q-PCRs result for 20 candidate cycling mRNAs. Two panels are shown side-by-side for each mRNA containing the sequencing (left) and Q-PCR (right) results. In the Q-PCR graphs, mRNA abundance at the first time point (CT0) serves as a reference, and is thus designated a value of 1. Abundances at other time points are plotted relative to the value at CT0. Negative and positive error bars show the range of possible relative values calculated based on the SEM of the Ct values obtained in the Q-PCR experiments. n≥4 for all time points. (TIF) [file pbio.1001703.s002.tif]

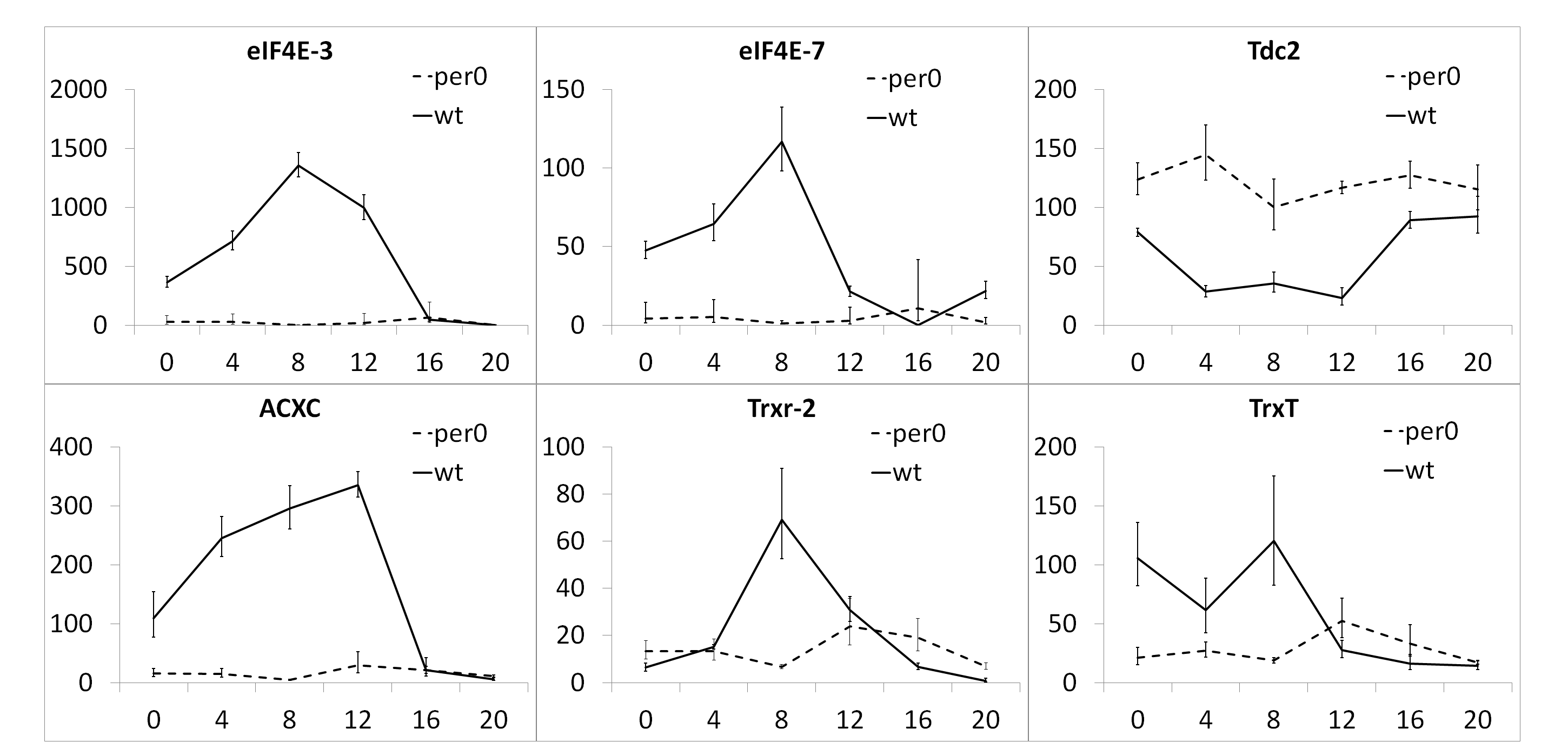

Supplement: Figure S3 — Q-PCR analyses for six rhythmic mRNAs in wild-type and the per0 mutant during the first day of DD. Relative abundances were calculated based on comparison to that of a noncycling gene, Rp49. Negative and positive error bars show the range of possible relative values calculated based on the SEM of the Ct values obtained in the actual Q-PCR experiments. n≥4 for all mRNAs analyzed. (TIF) [file pbio.1001703.s003.tif]

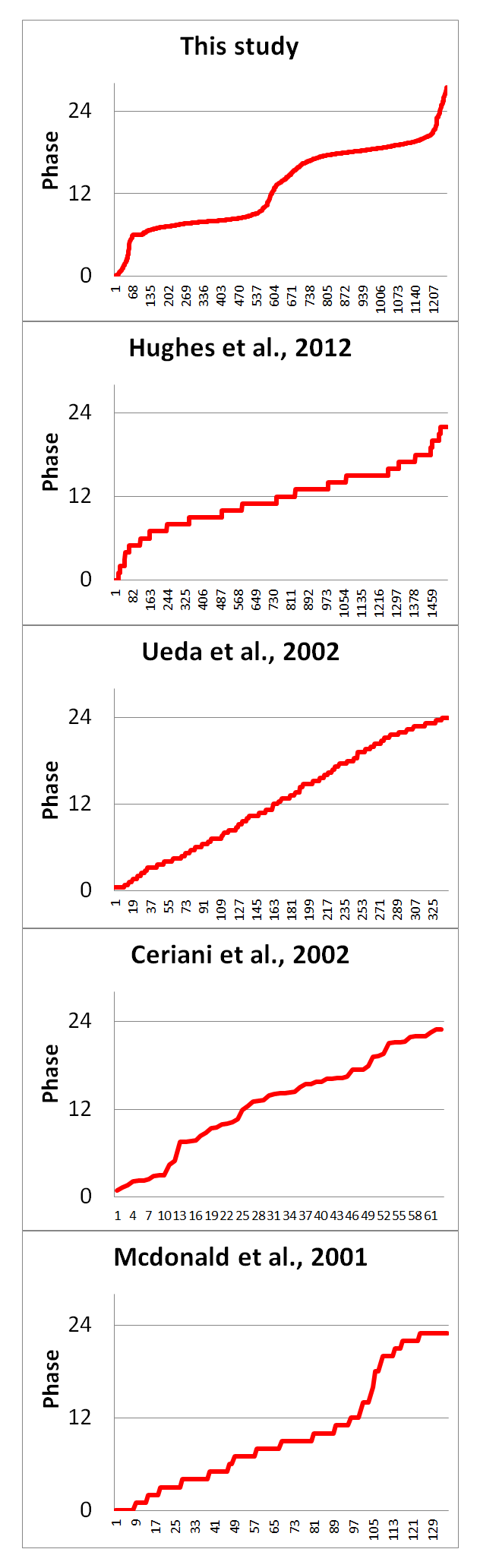

Supplement: Figure S4 — Phase comparisons of translation (this study) and mRNA abundance rhythms documented in several previous studies. Cycling mRNAs are arranged along the x-axis according to their phases, shown on the y-axis. (TIF) [file pbio.1001703.s004.tif]

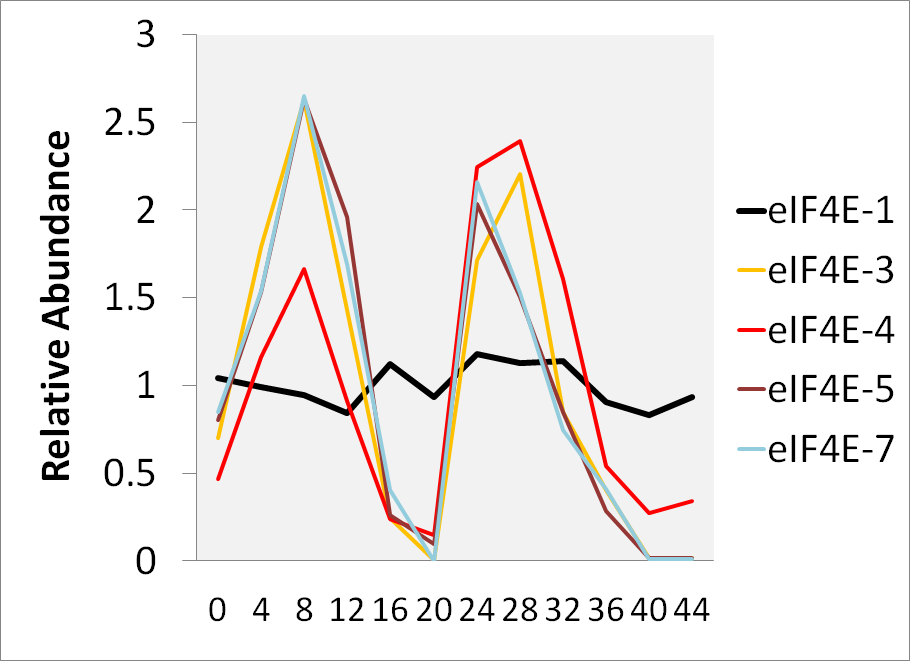

Supplement: Figure S5 — Rhythmic translation of the eIF-4E mRNAs. For each mRNA, translational level at each time point is normalized to the average translation across the time series. (TIF) [file pbio.1001703.s005.tif]

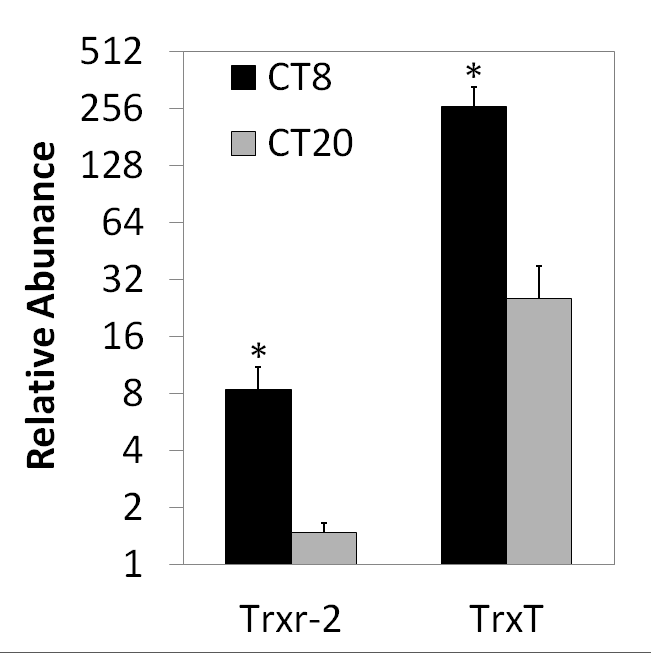

Supplement: Figure S6 — Q-PCR analyses of transcript abundance for Trxr-2 and TrxT in total RNA samples collected at two different time points: CT8 and CT20. n = 4 for all time points. Error bars represent SEM. *p<0.001 (Student's t test). (TIF) [file pbio.1001703.s006.tif]

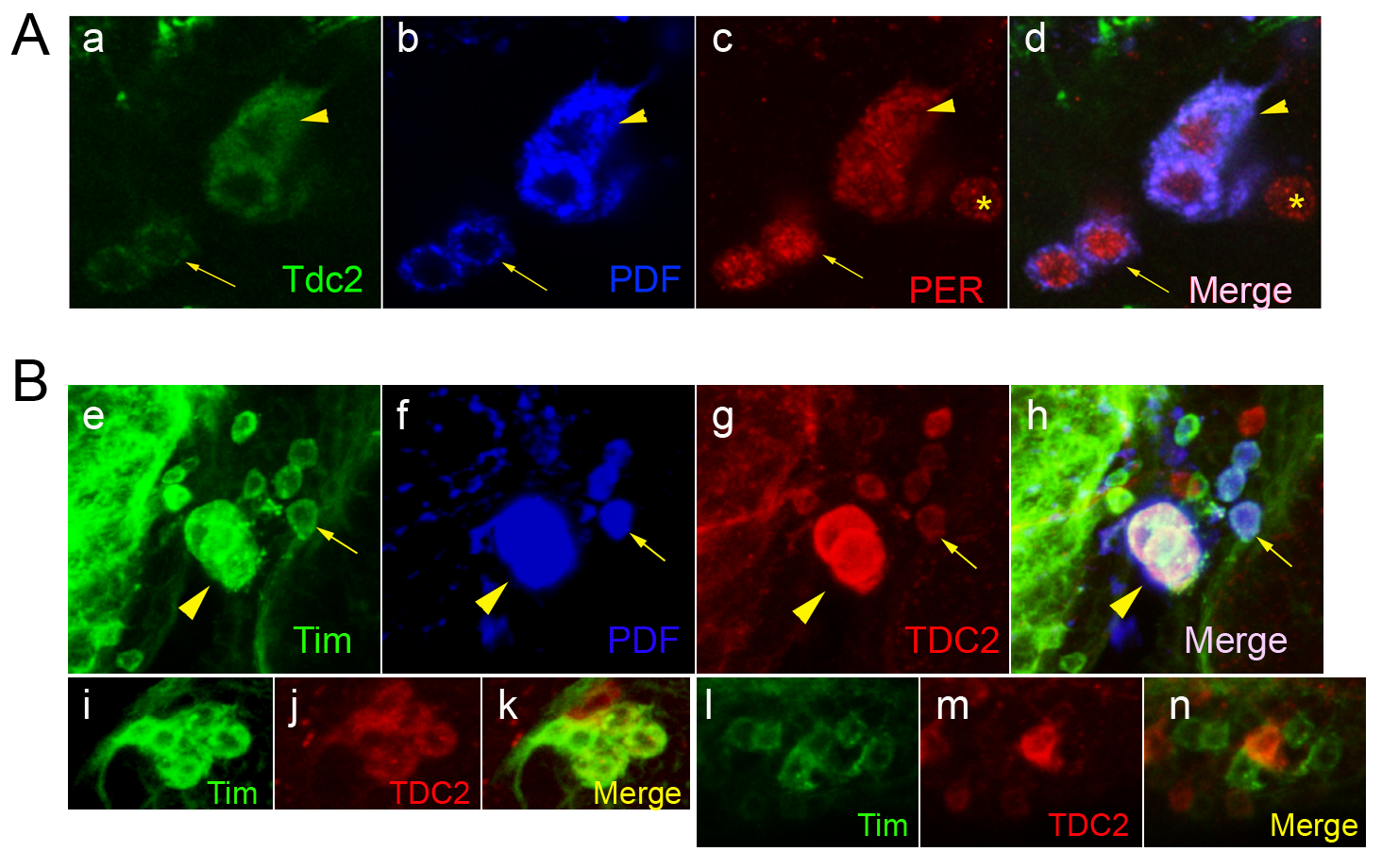

Supplement: Figure S7 — Tdc2 mRNA and protein are expressed in several groups of clock neurons including the PDF positive large and small ventral lateral neurons (LNvs), the dorsal lateral neurons (LNds), and dorsal neurons (DNs). (A) Expression of Tdc2-gal4 in the PDF neurons. Green, expression of mCD8-GFP driven by Tdc2-gal4; blue, PDF neuropeptide detected by anti-PDF antibody; red, PER protein detected by anti-PER antibody; arrow head, large LNvs; arrow, small LNvs; asterisk, PDF-negative small LNv. (B) Expression of TDC2 protein in LNvs (e–h), LNds (i–k), and DNs (l–m). Green, expression of mCD8-GFP driven by tim-uas-gal4 (for marking all clock cells); blue, PDF neuropeptide detected by anti-PDF antibody; red, TDC2 protein detected by anti-TDC2 antibody; arrowhead, large LNvs; arrow, small LNvs. (TIF) [file pbio.1001703.s007.tif]

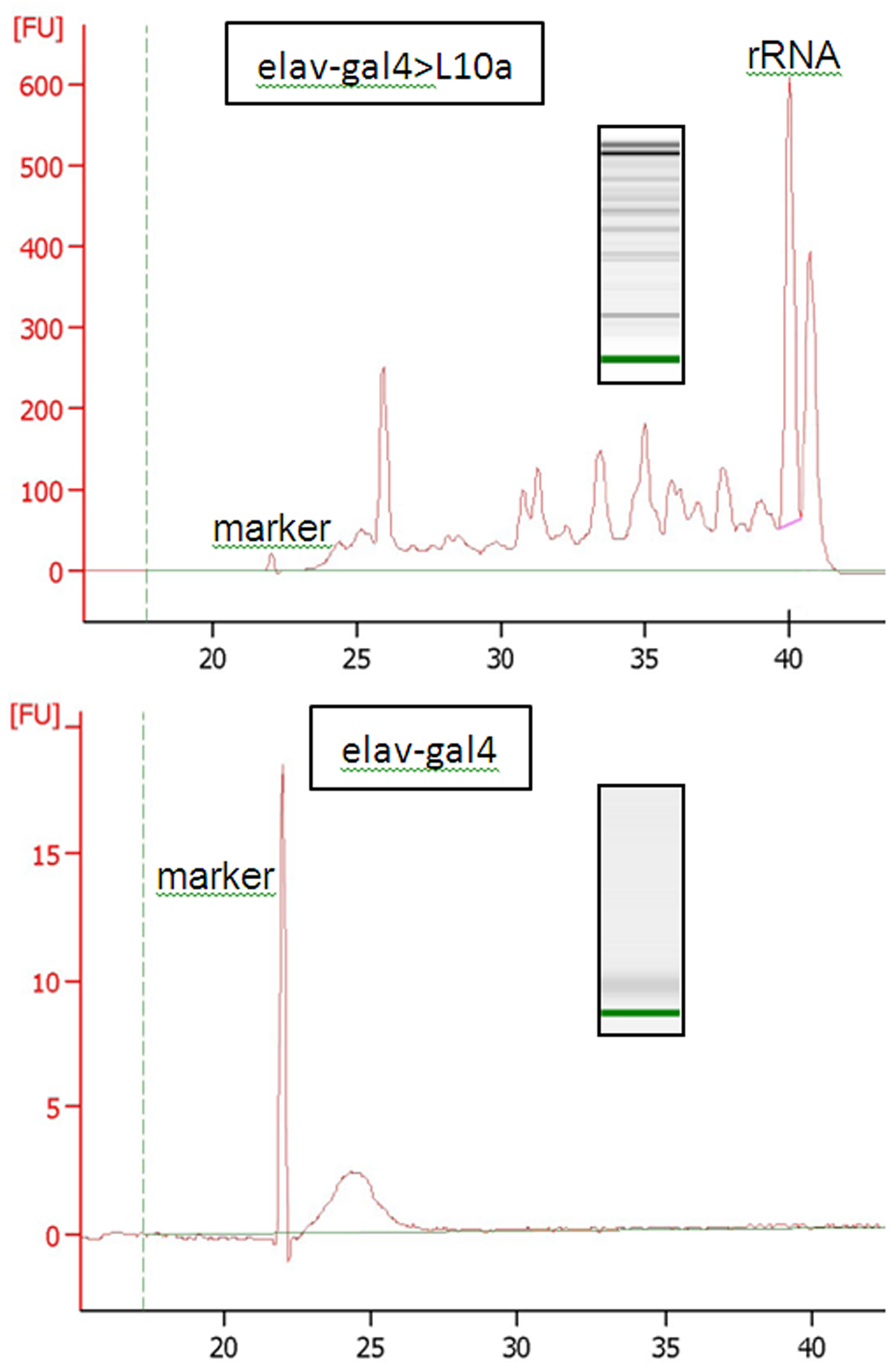

Supplement: Figure S8 — Using the TRAP technique, RNAs can be isolated from flies expressing EGFP-L10a but not from control flies without the transgene. Note the difference in the scale of the y-axis. (TIF) [file pbio.1001703.s008.tif]

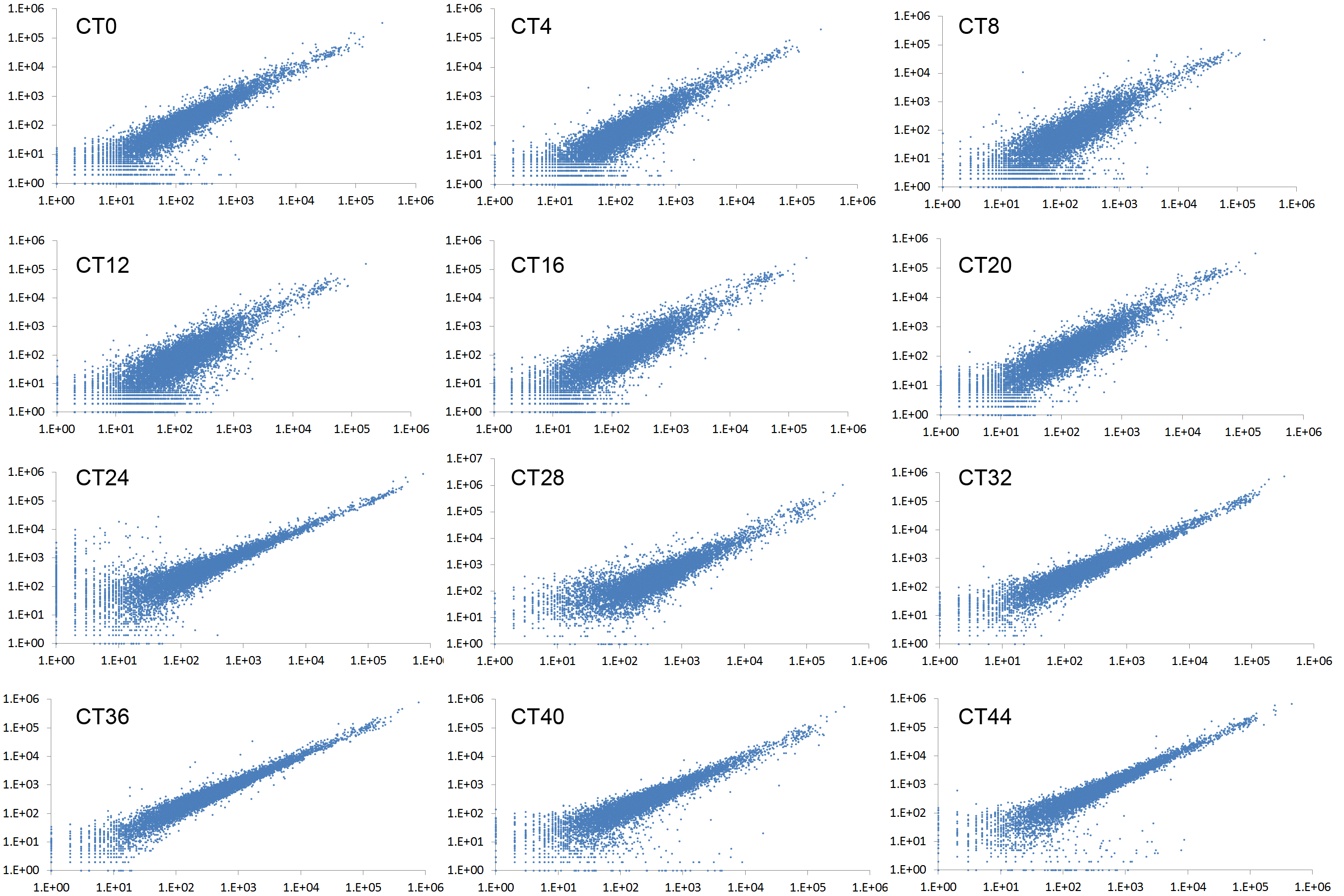

Supplement: Figure S9 — Scatter plots of read counts for all genes in two independent biological samples (sample 1, vertical axis; sample 2, horizontal axis) for all time points analyzed. (TIF) [file pbio.1001703.s009.tif]
