# Supplementary material for: Translational Profiling of Clock Cells Reveals Circadianly Synchronized Protein Synthesis
Source: PLoS Biol. 2013 Nov 5;11(11):e1001703. doi: 10.1371/journal.pbio.1001703 (PMC3864454; doi:10.1371/journal.pbio.1001703)
Supplement: Table S1 — RNA-seq statistics for all samples. (DOCX) [file pbio.1001703.s010.docx]

**Table S1. RNA-seq statistics for all samples.**

| **Sample** | **# of raw reads** | **Mean quality score** | **# of usable reads** | **% usable reads** | **# of mapped reads** | **% mapped** | **# of uniquely mapped** | **% uniquely mapped** |
| --- | --- | --- | --- | --- | --- | --- | --- | --- |
| CT00-1 | 20,714,235 | 30.43 | 13,260,728 | 64.02% | 10,382,156 | 78% | 10,136,553 | 76% |
| CT04-1 | 29,655,662 | 29.31 | 19,478,252 | 65.68% | 8,371,842 | 43% | 8,236,369 | 42% |
| CT08-1 | 20,052,247 | 30.55 | 13,028,729 | 64.97% | 8,856,023 | 68% | 8,520,266 | 65% |
| CT12-1 | 14,405,229 | 30.43 | 9,174,186 | 63.69% | 7,368,635 | 80% | 7,018,597 | 77% |
| CT16-1 | 18,798,083 | 30.29 | 11,840,010 | 62.99% | 9,304,214 | 79% | 9,045,493 | 76% |
| CT20-1 | 15,289,081 | 30.31 | 9,759,728 | 63.83% | 7,672,485 | 79% | 7,507,197 | 77% |
| CT00-2 | 11,514,418 | 33.96 | 11,481,934 | 99.72% | 10,063,326 | 88% | 9,314,919 | 81% |
| CT04-2 | 7,281,411 | 33.59 | 7,261,069 | 99.72% | 6,424,124 | 88% | 5,729,700 | 79% |
| CT08-2 | 9,264,953 | 32.65 | 9,238,795 | 99.72% | 7,711,132 | 83% | 6,537,994 | 71% |
| CT12-2 | 6,554,724 | 33.40 | 6,536,035 | 99.71% | 5,892,310 | 90% | 5,520,404 | 84% |
| CT16-2 | 12,994,504 | 32.18 | 12,957,823 | 99.72% | 10,729,190 | 83% | 9,957,092 | 77% |
| CT20-2 | 14,178,698 | 32.68 | 14,138,566 | 99.72% | 11,964,279 | 85% | 11,528,008 | 82% |
| CT24-1 | 39,713,359 | 34.48 | 39,700,997 | 99.97% | 32,487,012 | 82% | 30,761,954 | 77% |
| CT28-1 | 38,795,494 | 34.94 | 38,792,688 | 99.99% | 31,446,794 | 81% | 28,129,581 | 73% |
| CT32-1 | 31,503,593 | 34.62 | 31,501,968 | 99.99% | 28,746,021 | 91% | 26,853,168 | 85% |
| CT36-1 | 32,751,327 | 34.29 | 32,749,807 | 100.00% | 29,669,433 | 91% | 28,623,742 | 87% |
| CT40-1 | 23,129,545 | 34.56 | 23,127,987 | 99.99% | 16,723,154 | 72% | 15,858,282 | 69% |
| CT44-1 | 41,035,465 | 34.21 | 41,034,132 | 100.00% | 36,678,592 | 89% | 35,772,224 | 87% |
| CT24-2 | 32,038,496 | 34.38 | 32,032,814 | 99.98% | 28,904,092 | 90% | 27,279,643 | 85% |
| CT28-2 | 21,834,550 | 34.27 | 21,833,552 | 100.00% | 19,531,230 | 89% | 19,161,833 | 88% |
| CT32-2 | 17,409,802 | 34.26 | 17,408,777 | 99.99% | 15,652,686 | 90% | 15,356,835 | 88% |
| CT36-2 | 41,161,961 | 33.90 | 41,159,757 | 99.99% | 30,033,626 | 73% | 28,221,566 | 69% |
| CT40-2 | 20,508,259 | 34.75 | 20,507,665 | 100.00% | 18,852,833 | 92% | 17,613,023 | 86% |
| CT44-2 | 27,072,280 | 34.33 | 27,069,004 | 99.99% | 20,179,395 | 75% | 19,386,360 | 72% |
