# Supplementary material for: Translational Profiling of Clock Cells Reveals Circadianly Synchronized Protein Synthesis
Source: PLoS Biol. 2013 Nov 5;11(11):e1001703. doi: 10.1371/journal.pbio.1001703 (PMC3864454; doi:10.1371/journal.pbio.1001703)
Supplement: Table S2 — Correlation coefficients for two independent samples for each circadian time point. (DOCX) [file pbio.1001703.s011.docx]

**Table S2. Correlation coefficients for two independent samples for each circadian time point.**

| Time point | R |
| --- | --- |
| CT00 | 0.930 |
| CT04 | 0.947 |
| CT08 | 0.904 |
| CT12 | 0.915 |
| CT16 | 0.921 |
| CT20 | 0.928 |
| CT24 | 0.970 |
| CT28 | 0.904 |
| CT32 | 0.954 |
| CT36 | 0.976 |
| CT40 | 0.917 |
| CT44 | 0.966 |
