# Supplementary material for: Translational Profiling of Clock Cells Reveals Circadianly Synchronized Protein Synthesis
Source: PLoS Biol. 2013 Nov 5;11(11):e1001703. doi: 10.1371/journal.pbio.1001703 (PMC3864454; doi:10.1371/journal.pbio.1001703)
Supplement: Table S4 — All identified cycling mRNAs. (DOCX) [file pbio.1001703.s013.docx]

**Table S4. All identified cycling mRNAs.**

| **Flybase_ID** | **Gene_Symbol** | **pvalue** | **period** | **phase** | **amp** | **known cycling gene** | **LARK target** |
| --- | --- | --- | --- | --- | --- | --- | --- |
| **FBgn0000097** | **aop** | **1.2712E-05** | **20** | **18** | **1.08** | **yes** |  |
| **FBgn0001217** | **Hsc70-2** | **0.00512752** | **23** | **6** | **1.16** |  |  |
| **FBgn0003002** | **opa** | **0.00061488** | **20** | **19** | **0.97** |  |  |
| **FBgn0003068** | **per** | **0.00188985** | **21** | **13** | **0.91** | **yes** |  |
| **FBgn0029722** | **CG7024** | **0.00023766** | **21** | **7** | **0.64** |  |  |
| **FBgn0029750** | **CG3323** | **0.00120814** | **22** | **7** | **1.68** |  |  |
| **FBgn0029789** | **CG3160** | **0.00337723** | **22** | **19** | **0.66** |  |  |
| **FBgn0030001** | **cyr** | **0.0025887** | **24** | **16** | **1.72** |  |  |
| **FBgn0030034** | **CG10555** | **0.00798139** | **21** | **17** | **0.78** |  |  |
| **FBgn0013973** | **Gycbeta100B** | **0.00690958** | **22** | **18** | **0.78** |  |  |
| **FBgn0004456** | **mew** | **0.00047616** | **20** | **18** | **0.77** | **yes** |  |
| **FBgn0004652** | **fru** | **0.01343564** | **23** | **19** | **1.00** | **yes** |  |
| **FBgn0005658** | **Ets65A** | **0.00032993** | **20** | **18** | **1.12** |  |  |
| **FBgn0005660** | **Ets21C** | **0.00231473** | **21** | **20** | **1.60** |  |  |
| **FBgn0010383** | **Cyp18a1** | **0.00302855** | **21** | **18** | **0.67** | **yes** |  |
| **FBgn0011270** | **Pglym87** | **0.00170804** | **21** | **8** | **1.85** |  |  |
| **FBgn0011674** | **insc** | **0.00038765** | **22** | **18** | **0.80** |  |  |
| **FBgn0028700** | **RfC38** | **0.00050221** | **20** | **19** | **1.01** |  |  |
| **FBgn0028868** | **CG4701** | **0.00132424** | **21** | **8** | **2.36** |  |  |
| **FBgn0028892** | **CG4161** | **0.00314066** | **21** | **8** | **1.30** |  |  |
| **FBgn0015316** | **Try29F** | **0.00014316** | **21** | **1** | **0.55** |  |  |
| **FBgn0015380** | **drl** | **0.00448212** | **21** | **18** | **0.78** | **yes** | **Yes** |
| **FBgn0015831** | **Rtnl2** | **0.00320411** | **20** | **8** | **2.36** |  |  |
| **FBgn0016076** | **vri** | **0.00356537** | **23** | **15** | **0.98** | **yes** |  |
| **FBgn0016684** | **NaPi-T** | **0.00094817** | **22** | **9** | **1.93** |  |  |
| **FBgn0017556** | **Pros28.1B** | **0.00323371** | **21** | **8** | **1.13** |  |  |
| **FBgn0020280** | **laf** | **0.00086968** | **21** | **8** | **1.46** |  |  |
| **FBgn0023130** | **a6** | **0.00053569** | **22** | **17** | **0.60** |  |  |
| **FBgn0024332** | **Mcm3** | **3.3281E-05** | **22** | **21** | **0.78** |  |  |
| **FBgn0026314** | **Ugt35b** | **8.8829E-05** | **22** | **3** | **0.71** | **yes** |  |
| **FBgn0031085** | **CG9570** | **0.01033874** | **21** | **7** | **2.53** |  |  |
| **FBgn0029501** | **Crtp** | **0.00033354** | **21** | **8** | **1.63** |  |  |
| **FBgn0030384** | **CG2577** | **0.00030511** | **20** | **8** | **1.36** |  |  |
| **FBgn0030441** | **CG2209** | **0.00757212** | **20** | **8** | **2.81** |  |  |
| **FBgn0032047** | **CG13088** | **0.00268702** | **21** | **7** | **0.92** |  |  |
| **FBgn0032055** | **CG13091** | **0.00685532** | **21** | **8** | **1.54** |  |  |
| **FBgn0032266** | **CG18302** | **0.00322093** | **25** | **15** | **0.75** | **yes** |  |
| **FBgn0032275** | **CG17097** | **0.00398373** | **20** | **10** | **1.94** |  |  |
| **FBgn0032276** | **CG17098** | **0.01159435** | **21** | **7** | **2.31** |  |  |
| **FBgn0032286** | **CG7300** | **0.00142635** | **23** | **20** | **0.72** |  |  |
| **FBgn0030673** | **CG15601** | **0.01867436** | **20** | **18** | **1.30** |  |  |
| **FBgn0030697** | **CG8565** | **0.00129464** | **20** | **8** | **1.93** |  |  |
| **FBgn0030798** | **CG13003** | **0.00273324** | **22** | **19** | **1.65** |  |  |
| **FBgn0031849** | **CG11327** | **0.00052186** | **20** | **8** | **1.66** |  |  |
| **FBgn0031855** | **CG11221** | **0.01116696** | **22** | **18** | **0.78** | **yes** |  |
| **FBgn0031872** | **ihog** | **0.00138541** | **23** | **20** | **1.31** |  |  |
| **FBgn0031895** | **CG4497** | **0.00727187** | **20** | **0** | **0.71** |  |  |
| **FBgn0031920** | **CG6441** | **0.0004741** | **21** | **8** | **1.53** |  |  |
| **FBgn0031925** | **Cyp4d21** | **0.00322995** | **24** | **19** | **1.09** | **yes** |  |
| **FBgn0030958** | **CG6900** | **0.00642909** | **23** | **18** | **1.24** |  |  |
| **FBgn0030964** | **Pvf1** | **0.0027038** | **22** | **18** | **0.56** | **yes** |  |
| **FBgn0031323** | **CG5139** | **0.00044578** | **20** | **8** | **2.14** |  |  |
| **FBgn0031410** | **CG17237** | **0.01892398** | **22** | **7** | **2.95** |  |  |
| **FBgn0031504** | **CG15403** | **0.00331866** | **21** | **7** | **2.62** |  |  |
| **FBgn0031545** | **CG3213** | **0.00033863** | **20** | **8** | **2.06** |  |  |
| **FBgn0031785** | **CG13991** | **0.00278697** | **21** | **8** | **1.76** |  |  |
| **FBgn0032381** | **Mal-B1** | **0.0003048** | **23** | **19** | **0.82** |  |  |
| **FBgn0032471** | **CG5122** | **0.00268667** | **21** | **7** | **1.98** |  |  |
| **FBgn0032520** | **CG10859** | **0.00215289** | **20** | **8** | **1.57** |  |  |
| **FBgn0032525** | **Hsp60D** | **0.02075889** | **26** | **8** | **2.00** |  |  |
| **FBgn0032624** | **CG6304** | **0.0166854** | **22** | **7** | **2.04** |  |  |
| **FBgn0032637** | **CG5050** | **0.0003556** | **21** | **7** | **1.31** |  |  |
| **FBgn0032648** | **CG15144** | **0.01241104** | **22** | **6** | **2.56** |  |  |
| **FBgn0032677** | **CG5790** | **0.00386959** | **23** | **6** | **1.33** |  |  |
| **FBgn0032705** | **Grip71** | **0.0165351** | **27** | **13** | **0.64** |  |  |
| **FBgn0032779** | **CG16771** | **0.00070649** | **21** | **18** | **0.74** |  |  |
| **FBgn0032856** | **CG16798** | **0.00086168** | **26** | **23** | **0.82** |  |  |
| **FBgn0033056** | **CG7856** | **0.00542163** | **21** | **8** | **1.99** |  |  |
| **FBgn0033365** | **CG8170** | **0.00447262** | **22** | **19** | **1.23** |  |  |
| **FBgn0034053** | **Cyp4aa1** | **0.00019018** | **21** | **0** | **0.72** |  |  |
| **FBgn0036278** | **GRHRII** | **0.00650913** | **21** | **19** | **0.70** | **yes** |  |
| **FBgn0036321** | **CG14120** | **2.4215E-05** | **21** | **10** | **1.88** |  |  |
| **FBgn0050090** | **CG30090** | **0.00314306** | **22** | **18** | **0.98** |  |  |
| **FBgn0033679** | **CG8888** | **0.00452896** | **20** | **18** | **0.88** | **yes** |  |
| **FBgn0033706** | **Vha36-2** | **0.00030956** | **21** | **9** | **1.49** |  |  |
| **FBgn0033742** | **CG8550** | **0.01248496** | **23** | **17** | **0.70** | **yes** |  |
| **FBgn0033856** | **CG13334** | **0.01425192** | **23** | **6** | **2.27** |  |  |
| **FBgn0033888** | **CG18568** | **0.00291663** | **20** | **8** | **2.14** |  |  |
| **FBgn0034009** | **CG8155** | **0.00444978** | **21** | **18** | **0.57** |  |  |
| **FBgn0034105** | **CG7755** | **0.0069529** | **23** | **6** | **1.46** |  |  |
| **FBgn0034122** | **CG15711** | **0.01985166** | **21** | **7** | **2.49** |  |  |
| **FBgn0034127** | **CG7848** | **0.00056964** | **21** | **8** | **0.98** |  |  |
| **FBgn0034374** | **CG15086** | **0.00122577** | **22** | **7** | **1.51** |  |  |
| **FBgn0034416** | **CG15109** | **0.00658474** | **21** | **7** | **1.73** |  |  |
| **FBgn0034540** | **Lrt** | **0.00600679** | **22** | **16** | **0.74** |  |  |
| **FBgn0034657** | **LBR** | **0.0021556** | **22** | **18** | **2.02** |  |  |
| **FBgn0034816** | **CG3085** | **0.01914722** | **22** | **8** | **2.01** |  |  |
| **FBgn0034824** | **Klp59C** | **0.00288793** | **20** | **8** | **1.83** |  |  |
| **FBgn0034827** | **Klp59D** | **0.00083129** | **20** | **8** | **1.47** |  |  |
| **FBgn0034844** | **CG9861** | **0.00269222** | **21** | **7** | **1.84** |  |  |
| **FBgn0034957** | **CG3121** | **0.00658199** | **20** | **8** | **3.21** |  |  |
| **FBgn0034979** | **CG13569** | **0.01516406** | **23** | **6** | **2.28** |  |  |
| **FBgn0035218** | **CG9173** | **0.00098885** | **22** | **8** | **1.50** |  |  |
| **FBgn0035309** | **CG15879** | **0.00025489** | **21** | **17** | **1.02** |  |  |
| **FBgn0035355** | **CG16985** | **0.00976898** | **22** | **6** | **0.62** |  |  |
| **FBgn0035380** | **CG9970** | **0.00480632** | **21** | **7** | **2.94** |  |  |
| **FBgn0035427** | **ckd** | **6.7986E-05** | **20** | **18** | **1.19** |  |  |
| **FBgn0035585** | **CG12027** | **0.00028326** | **21** | **8** | **1.10** |  |  |
| **FBgn0035619** | **CG10592** | **0.01245976** | **21** | **9** | **1.94** |  |  |
| **FBgn0035730** | **CG17744** | **0.00272809** | **22** | **7** | **2.24** |  |  |
| **FBgn0035860** | **eIF4E-3** | **0.00054064** | **21** | **8** | **1.69** |  |  |
| **FBgn0035921** | **CG13305** | **0.00252636** | **24** | **1** | **1.21** | **yes** |  |
| **FBgn0035977** | **PGRP-LF** | **0.00360278** | **21** | **17** | **0.62** |  |  |
| **FBgn0036014** | **CG3222** | **0.00334851** | **20** | **8** | **1.82** |  |  |
| **FBgn0036075** | **CG8065** | **0.00071132** | **21** | **8** | **1.39** |  |  |
| **FBgn0036179** | **CG7368** | **0.00224738** | **21** | **18** | **0.73** | **yes** |  |
| **FBgn0036345** | **CG17300** | **0.00079077** | **21** | **9** | **1.43** |  |  |
| **FBgn0036346** | **CG11251** | **0.00076066** | **23** | **7** | **1.09** |  |  |
| **FBgn0036564** | **Taspase1** | **0.01054128** | **22** | **18** | **0.76** |  |  |
| **FBgn0036652** | **CG13032** | **0.00211007** | **20** | **8** | **1.50** |  |  |
| **FBgn0036660** | **CG13025** | **0.00393406** | **22** | **18** | **1.13** |  |  |
| **FBgn0036708** | **CG13725** | **0.00108481** | **20** | **8** | **1.27** |  |  |
| **FBgn0036778** | **Cyp312a1** | **0.00559802** | **20** | **8** | **1.94** |  |  |
| **FBgn0036899** | **tey** | **0.01274537** | **24** | **19** | **1.29** |  |  |
| **FBgn0036941** | **CG7335** | **0.0041637** | **20** | **7** | **2.76** |  |  |
| **FBgn0037000** | **CG5130** | **0.01200631** | **21** | **19** | **0.71** |  |  |
| **FBgn0037040** | **CG12983** | **8.2318E-05** | **21** | **8** | **1.69** |  |  |
| **FBgn0037064** | **CG9389** | **0.01709643** | **20** | **7** | **1.50** | **yes** |  |
| **FBgn0037151** | **CG7130** | **0.00037671** | **22** | **18** | **0.71** |  |  |
| **FBgn0037170** | **Trxr-2** | **0.00245505** | **21** | **8** | **1.82** |  |  |
| **FBgn0037388** | **CG14676** | **0.00128949** | **21** | **8** | **1.96** |  |  |
| **FBgn0037398** | **CG15580** | **0.00508569** | **21** | **7** | **2.33** |  |  |
| **FBgn0037461** | **CG15177** | **0.00039113** | **21** | **8** | **1.91** |  |  |
| **FBgn0037519** | **CG3014** | **0.00038862** | **20** | **11** | **1.22** |  |  |
| **FBgn0037579** | **CG18193** | **0.00687915** | **20** | **8** | **1.39** |  |  |
| **FBgn0037617** | **CG8145** | **0.00055995** | **21** | **18** | **0.94** |  |  |
| **FBgn0037986** | **CG14736** | **0.00596849** | **23** | **8** | **2.35** |  |  |
| **FBgn0037988** | **CG14740** | **0.00073779** | **20** | **9** | **1.72** |  |  |
| **FBgn0038018** | **Tim17a1** | **0.00359873** | **21** | **8** | **1.32** |  |  |
| **FBgn0038051** | **CG17207** | **0.01743406** | **21** | **20** | **1.45** |  |  |
| **FBgn0038125** | **CG8141** | **0.01586625** | **21** | **9** | **4.86** |  |  |
| **FBgn0038136** | **CG8774** | **0.00100902** | **21** | **9** | **1.44** |  |  |
| **FBgn0038163** | **CG10841** | **0.00189671** | **21** | **7** | **1.68** |  |  |
| **FBgn0038208** | **CG14355** | **0.00318727** | **22** | **7** | **1.51** |  |  |
| **FBgn0038373** | **CG4546** | **0.00741969** | **21** | **8** | **1.60** |  |  |
| **FBgn0038402** | **Fer2** | **0.00065234** | **21** | **20** | **0.98** | **yes** |  |
| **FBgn0038404** | **CG8925** | **0.00098147** | **28** | **18** | **1.04** |  |  |
| **FBgn0038709** | **CG15025** | **0.01001255** | **21** | **7** | **2.71** |  |  |
| **FBgn0038732** | **CG11391** | **0.00156651** | **25** | **20** | **1.04** |  |  |
| **FBgn0038862** | **Ubpy** | **2.2285E-05** | **22** | **12** | **0.98** |  |  |
| **FBgn0038874** | **ETHR** | **0.0050868** | **23** | **16** | **0.72** |  |  |
| **FBgn0038892** | **CG15498** | **0.00864631** | **21** | **7** | **2.28** |  |  |
| **FBgn0039088** | **CG10164** | **0.0021189** | **21** | **7** | **2.14** |  |  |
| **FBgn0039184** | **CG6432** | **0.00101114** | **22** | **9** | **1.47** |  |  |
| **FBgn0039310** | **CG11878** | **0.00023099** | **22** | **9** | **1.23** |  |  |
| **FBgn0039398** | **CG14540** | **0.01397713** | **21** | **7** | **2.12** |  |  |
| **FBgn0039421** | **CG6036** | **0.00047989** | **21** | **7** | **1.45** |  |  |
| **FBgn0039498** | **CG17991** | **0.00211958** | **20** | **8** | **1.29** |  |  |
| **FBgn0039553** | **CG5017** | **0.00323362** | **21** | **8** | **2.44** |  |  |
| **FBgn0039752** | **CG15530** | **0.00191158** | **20** | **8** | **1.90** |  |  |
| **FBgn0040347** | **mus81** | **0.01296166** | **23** | **19** | **1.19** |  |  |
| **FBgn0040356** | **CG12498** | **0.00622305** | **23** | **6** | **1.63** |  |  |
| **FBgn0040508** | **ACXC** | **3.7192E-05** | **20** | **9** | **1.85** |  |  |
| **FBgn0050418** | **nord** | **0.00326759** | **24** | **19** | **0.94** |  |  |
| **FBgn0050446** | **Tdc2** | **0.01213424** | **24** | **20** | **1.53** | **yes** |  |
| **FBgn0051146** | **Nlg1** | **0.00175868** | **21** | **18** | **0.92** |  |  |
| **FBgn0051294** | **CG31294** | **0.01261962** | **21** | **7** | **2.26** |  |  |
| **FBgn0051343** | **CG31343** | **0.00096939** | **21** | **10** | **1.96** |  |  |
| **FBgn0051533** | **CG31533** | **0.00069552** | **20** | **8** | **1.60** |  |  |
| **FBgn0051538** | **CG31538** | **0.00259062** | **22** | **7** | **1.45** |  |  |
| **FBgn0051679** | **CG31679** | **0.00040547** | **20** | **8** | **1.62** |  |  |
| **FBgn0051735** | **CG31735** | **0.01254962** | **22** | **6** | **1.86** |  |  |
| **FBgn0051803** | **CG31803** | **0.00021035** | **20** | **9** | **1.76** |  |  |
| **FBgn0051804** | **CG31804** | **0.00379405** | **22** | **6** | **1.63** |  |  |
| **FBgn0051812** | **CG31812** | **2.1924E-05** | **20** | **19** | **0.71** |  |  |
| **FBgn0051814** | **CG31814** | **0.00453825** | **24** | **16** | **1.32** |  |  |
| **FBgn0051907** | **CG31907** | **0.00585113** | **21** | **8** | **3.04** |  |  |
| **FBgn0051920** | **CG31920** | **0.00013213** | **21** | **8** | **0.87** |  |  |
| **FBgn0051924** | **CG31924** | **0.00375606** | **21** | **7** | **2.01** |  |  |
| **FBgn0051948** | **CG31948** | **0.00750046** | **20** | **8** | **2.01** |  |  |
| **FBgn0051954** | **CG31954** | **0.0062811** | **23** | **17** | **2.14** |  |  |
| **FBgn0052061** | **CG32061** | **0.00314454** | **21** | **9** | **0.97** |  |  |
| **FBgn0052105** | **CG32105** | **0.00251902** | **24** | **19** | **0.83** |  |  |
| **FBgn0052529** | **CG32529** | **0.00106093** | **21** | **17** | **0.86** |  |  |
| **FBgn0052656** | **Muc11A** | **0.00865486** | **20** | **9** | **1.37** |  |  |
| **FBgn0052806** | **CG32806** | **0.00795498** | **21** | **7** | **1.64** |  |  |
| **FBgn0052832** | **CG32832** | **0.00201835** | **20** | **9** | **2.32** |  |  |
| **FBgn0053057** | **CG33057** | **8.1206E-06** | **21** | **19** | **0.71** |  |  |
| **FBgn0053092** | **P5CDh2** | **0.00517972** | **21** | **8** | **1.59** |  |  |
| **FBgn0058006** | **CG40006** | **0.01095625** | **20** | **18** | **0.62** |  |  |
| **FBgn0062412** | **Ctr1B** | **0.00690569** | **22** | **19** | **0.96** |  |  |
| **FBgn0063499** | **GstE10** | **0.00614803** | **21** | **10** | **1.31** |  |  |
| **FBgn0085248** | **CG34219** | **0.00521032** | **22** | **19** | **1.65** |  |  |
| **FBgn0085426** | **Rgk3** | **0.01233563** | **23** | **18** | **1.66** | **yes** |  |
| **FBgn0085480** | **CG34451** | **0.0022862** | **24** | **19** | **1.20** |  |  |
| **FBgn0085486** | **CG34457** | **0.00081462** | **21** | **7** | **1.73** |  |  |
| **FBgn0087040** | **alphaTub67C** | **0.0051905** | **23** | **15** | **1.06** |  |  |
| **FBgn0250827** | **whip** | **0.00686353** | **21** | **8** | **3.90** |  |  |
| **FBgn0250846** | **glob2** | **0.0171116** | **21** | **7** | **2.55** |  |  |
| **FBgn0259216** | **RhoGAP102A** | **0.00340174** | **22** | **18** | **1.16** |  |  |
| **FBgn0259224** | **CG42324** | **0.0037806** | **22** | **17** | **0.57** |  |  |
| **FBgn0259713** | **CG42367** | **4.668E-05** | **23** | **21** | **1.59** |  |  |
| **FBgn0259918** | **inaF-B** | **0.00342465** | **22** | **18** | **1.98** |  |  |
| **FBgn0259922** | **CG42448** | **0.01216004** | **23** | **5** | **1.72** |  |  |
| **FBgn0260481** | **CG32454** | **0.00501044** | **21** | **8** | **2.47** |  |  |
| **FBgn0261085** | **Syt12** | **0.01954478** | **25** | **15** | **0.82** | **yes** |  |
| **FBgn0261277** | **rtv** | **0.01192639** | **24** | **17** | **1.01** |  |  |
| **FBgn0261882** | **l(2)35Bc** | **0.01008996** | **22** | **19** | **0.56** |  |  |
| **FBgn0262097** | **CG42850** | **0.00331571** | **21** | **7** | **1.56** |  |  |
| **FBgn0262535** | **CG43089** | **0.01247712** | **24** | **5** | **1.85** |  |  |
| **FBgn0262568** | **CG43108** | **0.001626** | **21** | **7** | **1.11** |  |  |
| **FBgn0262733** | **Src64B** | **0.00114952** | **20** | **16** | **0.79** | **yes** |  |
| **FBgn0263093** | **CR43361** | **0.0203255** | **21** | **18** | **1.07** |  |  |
| FBgn0000038 | nAcRbeta-64B | 0.01865338 | 20 | 10 | 0.81 | yes |  |
| FBgn0000055 | Adh | 0.00162609 | 21 | 18 | 0.80 | yes |  |
| FBgn0000241 | bw | 0.00571616 | 21 | 18 | 0.69 |  |  |
| FBgn0000242 | Bx | 0.00395092 | 21 | 18 | 1.24 | yes | yes |
| FBgn0000246 | c(3)G | 0.00022262 | 22 | 18 | 0.77 |  |  |
| FBgn0000251 | cad | 0.00269863 | 22 | 8 | 1.42 |  |  |
| FBgn0000253 | Cam | 0.01838674 | 28 | 27 | 1.37 |  | yes |
| FBgn0000274 | Pka-C2 | 0.01009956 | 21 | 7 | 1.81 |  |  |
| FBgn0000464 | Lar | 0.01979328 | 24 | 16 | 0.81 |  |  |
| FBgn0000490 | dpp | 0.00635525 | 21 | 18 | 1.01 |  |  |
| FBgn0000524 | dx | 0.00042695 | 22 | 17 | 0.76 |  |  |
| FBgn0000542 | ec | 0.0141271 | 24 | 18 | 0.84 |  |  |
| FBgn0000567 | Eip74EF | 0.00471693 | 24 | 13 | 0.75 | yes | yes |
| FBgn0000577 | en | 0.00879129 | 26 | 20 | 1.09 |  |  |
| FBgn0000578 | ena | 0.01529594 | 20 | 18 | 1.89 | yes |  |
| FBgn0000588 | esc | 0.01322088 | 20 | 19 | 0.57 |  |  |
| FBgn0000592 | Est-6 | 0.01952372 | 21 | 19 | 0.77 | yes |  |
| FBgn0000615 | exu | 0.0053877 | 20 | 8 | 0.83 |  |  |
| FBgn0000636 | Fas3 | 0.01529788 | 27 | 25 | 0.51 | yes |  |
| FBgn0000658 | fj | 0.00396718 | 22 | 18 | 1.14 |  |  |
| FBgn0000723 | Fps85D | 0.01351463 | 27 | 13 | 0.60 | yes |  |
| FBgn0000810 | fs(1)K10 | 0.00289715 | 21 | 1 | 0.52 |  |  |
| FBgn0000996 | dup | 0.00263628 | 21 | 16 | 0.74 |  |  |
| FBgn0001078 | ftz-f1 | 0.01385465 | 20 | 17 | 0.63 |  |  |
| FBgn0001084 | fy | 0.01826693 | 21 | 20 | 0.58 |  |  |
| FBgn0001112 | Gld | 0.00693119 | 24 | 19 | 0.93 |  |  |
| FBgn0001206 | Hmr | 0.00855147 | 22 | 16 | 0.70 |  |  |
| FBgn0001229 | Hsp67Bc | 5.0383E-05 | 20 | 17 | 0.72 |  |  |
| FBgn0001301 | kel | 0.00141013 | 21 | 18 | 0.53 | yes |  |
| FBgn0001404 | egh | 0.00686212 | 20 | 18 | 0.79 |  | yes |
| FBgn0001990 | wek | 0.01624099 | 20 | 20 | 0.74 |  |  |
| FBgn0002528 | LanB2 | 0.00919993 | 24 | 14 | 0.64 |  |  |
| FBgn0002543 | lea | 0.00207135 | 22 | 16 | 0.94 | yes |  |
| FBgn0002567 | ltd | 0.01344607 | 23 | 17 | 0.59 | yes |  |
| FBgn0002570 | Mal-A1 | 0.00742248 | 21 | 9 | 2.89 |  |  |
| FBgn0002609 | HLHm3 | 0.00083187 | 20 | 19 | 0.86 | yes |  |
| FBgn0002641 | mal | 0.01623011 | 23 | 14 | 0.55 |  |  |
| FBgn0002707 | mei-9 | 0.01818963 | 24 | 18 | 0.54 |  |  |
| FBgn0002723 | Met | 0.00975094 | 21 | 17 | 0.79 |  |  |
| FBgn0002842 | sa | 0.00910527 | 23 | 6 | 2.10 | yes |  |
| FBgn0002862 | Mst87F | 0.01131391 | 21 | 7 | 3.10 |  |  |
| FBgn0002865 | Mst98Ca | 0.00876727 | 20 | 8 | 1.74 |  |  |
| FBgn0002873 | mud | 0.00316712 | 20 | 9 | 2.63 |  |  |
| FBgn0002899 | mus301 | 0.01753477 | 24 | 17 | 0.85 |  |  |
| FBgn0002989 | okr | 0.01926729 | 20 | 18 | 0.95 |  |  |
| FBgn0003028 | ovo | 0.00432614 | 23 | 20 | 0.92 |  |  |
| FBgn0003041 | pbl | 0.0059635 | 27 | 12 | 0.89 |  |  |
| FBgn0003044 | Pcl | 0.00718623 | 22 | 18 | 1.01 | yes |  |
| FBgn0003060 | CG9757 | 0.00552395 | 24 | 20 | 1.81 |  |  |
| FBgn0003124 | polo | 0.00967349 | 21 | 7 | 1.92 |  |  |
| FBgn0003256 | rl | 0.00644537 | 24 | 11 | 0.55 |  |  |
| FBgn0003295 | ru | 0.01429647 | 21 | 0 | 0.92 |  |  |
| FBgn0003328 | scb | 0.00042471 | 28 | 12 | 0.60 |  |  |
| FBgn0003334 | Scm | 0.0005107 | 20 | 18 | 0.70 |  |  |
| FBgn0003339 | Scr | 0.00842842 | 20 | 18 | 0.96 |  |  |
| FBgn0003358 | Jon99Ci | 0.00293454 | 22 | 9 | 1.07 |  |  |
| FBgn0003396 | shn | 0.01753755 | 21 | 18 | 1.05 | yes |  |
| FBgn0003415 | skd | 0.0047825 | 26 | 16 | 0.93 |  |  |
| FBgn0003425 | sli | 0.00351033 | 20 | 18 | 1.07 |  |  |
| FBgn0003459 | stwl | 0.01633442 | 21 | 17 | 0.71 |  |  |
| FBgn0003507 | srp | 0.01932818 | 20 | 18 | 0.64 |  |  |
| FBgn0003638 | su(w[a]) | 0.00343473 | 20 | 10 | 0.56 |  |  |
| FBgn0003654 | sw | 0.00515928 | 22 | 15 | 0.53 |  |  |
| FBgn0003751 | trk | 0.0049441 | 21 | 19 | 1.06 |  |  |
| FBgn0003889 | betaTub85D | 0.00086988 | 20 | 8 | 1.46 |  |  |
| FBgn0003892 | ptc | 0.00715971 | 21 | 18 | 1.45 |  |  |
| FBgn0003961 | Uro | 0.01185466 | 24 | 7 | 4.42 |  |  |
| FBgn0003964 | usp | 0.01858533 | 21 | 18 | 0.72 |  |  |
| FBgn0003996 | w | 0.00868052 | 22 | 21 | 1.13 |  |  |
| FBgn0004108 | Nrt | 0.01867957 | 22 | 20 | 1.83 |  |  |
| FBgn0004168 | 5-HT1A | 0.01105121 | 22 | 17 | 0.65 | yes |  |
| FBgn0004171 | Mst98Cb | 0.01438091 | 21 | 8 | 2.08 |  |  |
| FBgn0004228 | mex1 | 0.02077069 | 24 | 7 | 3.03 |  |  |
| FBgn0004394 | pdm2 | 0.0080463 | 25 | 16 | 0.70 |  |  |
| FBgn0004395 | unk | 0.02082383 | 20 | 17 | 0.80 | yes |  |
| FBgn0004575 | Syn | 0.00138063 | 20 | 11 | 0.51 | yes | yes |
| FBgn0004882 | orb | 0.01834642 | 20 | 8 | 3.42 | yes |  |
| FBgn0004892 | sob | 0.00824928 | 21 | 19 | 1.19 |  |  |
| FBgn0004919 | gol | 0.00474997 | 24 | 13 | 0.78 | yes |  |
| FBgn0005696 | DNApol-alpha73 | 0.01580333 | 23 | 19 | 0.94 |  |  |
| FBgn0008654 | Su(z)2 | 0.00053199 | 20 | 17 | 0.79 | yes | yes |
| FBgn0010051 | Itp-r83A | 0.02063492 | 24 | 15 | 0.81 |  |  |
| FBgn0010052 | Jhe | 0.01189977 | 22 | 21 | 0.91 |  |  |
| FBgn0010194 | Wnt5 | 0.00671823 | 21 | 20 | 0.99 |  |  |
| FBgn0010241 | Mdr50 | 0.01930086 | 24 | 16 | 0.93 |  |  |
| FBgn0010246 | Myo61F | 0.00400746 | 25 | 9 | 0.61 |  |  |
| FBgn0010265 | RpS13 | 0.02055522 | 27 | 1 | 0.51 |  |  |
| FBgn0010269 | Dsor1 | 0.01149643 | 27 | 27 | 0.51 |  |  |
| FBgn0010315 | CycD | 0.0136057 | 21 | 17 | 0.76 | yes |  |
| FBgn0010341 | Cdc42 | 0.0030934 | 20 | 18 | 1.15 | yes |  |
| FBgn0010389 | htl | 0.00377892 | 22 | 21 | 0.77 |  |  |
| FBgn0010441 | pll | 0.0079731 | 21 | 18 | 0.61 | yes |  |
| FBgn0010812 | unc-45 | 0.00721386 | 22 | 19 | 0.51 |  |  |
| FBgn0011206 | bol | 0.00374873 | 21 | 8 | 1.55 |  |  |
| FBgn0011244 | Hsp60B | 0.00057912 | 21 | 8 | 1.56 |  |  |
| FBgn0011555 | thetaTry | 0.00413984 | 22 | 8 | 1.26 |  |  |
| FBgn0011589 | elk | 0.00835026 | 21 | 17 | 0.90 |  |  |
| FBgn0011648 | Mad | 0.00024473 | 20 | 17 | 0.51 | yes |  |
| FBgn0011703 | RnrL | 0.01702578 | 24 | 15 | 0.91 |  |  |
| FBgn0011743 | Arp53D | 0.0031252 | 21 | 8 | 1.92 |  |  |
| FBgn0011746 | ana | 0.01818739 | 21 | 19 | 1.22 | yes |  |
| FBgn0011747 | Ank | 0.00591986 | 24 | 14 | 0.51 | yes |  |
| FBgn0011823 | Pen | 0.00575276 | 22 | 7 | 1.98 |  |  |
| FBgn0013279 | Hsp70Bc | 0.00023316 | 21 | 18 | 0.76 |  |  |
| FBgn0013810 | Dhc36C | 0.01007319 | 20 | 8 | 2.54 |  |  |
| FBgn0014011 | Rac2 | 0.00882629 | 20 | 19 | 1.20 | yes | yes |
| FBgn0014340 | mof | 0.00071467 | 20 | 20 | 0.51 |  |  |
| FBgn0014464 | Cp7Fa | 0.01258359 | 21 | 20 | 1.31 |  |  |
| FBgn0014861 | Mcm2 | 0.0007349 | 22 | 17 | 0.85 |  |  |
| FBgn0015376 | cutlet | 0.00579283 | 24 | 15 | 0.61 |  |  |
| FBgn0015591 | Ast | 0.00213672 | 21 | 20 | 0.70 | yes |  |
| FBgn0015618 | Cdk8 | 9.3278E-05 | 21 | 20 | 0.92 |  |  |
| FBgn0015663 | Dot | 0.00337254 | 20 | 20 | 0.78 |  |  |
| FBgn0015904 | ara | 0.00492605 | 23 | 18 | 1.12 |  |  |
| FBgn0016059 | Sema-1b | 0.01906909 | 28 | 12 | 0.51 |  |  |
| FBgn0016061 | side | 0.00368475 | 23 | 17 | 0.94 | yes |  |
| FBgn0016641 | PTP-ER | 0.01065563 | 24 | 17 | 0.53 |  |  |
| FBgn0016797 | fz2 | 0.01971427 | 22 | 18 | 2.75 | yes |  |
| FBgn0016974 | swaPsi | 0.0054252 | 21 | 8 | 1.77 |  |  |
| FBgn0017448 | CG2187 | 0.00615726 | 22 | 9 | 2.41 |  |  |
| FBgn0020236 | ATPCL | 0.00087013 | 28 | 12 | 0.74 | yes |  |
| FBgn0020261 | pcm | 0.01769815 | 26 | 14 | 0.60 |  |  |
| FBgn0020299 | stumps | 0.02034579 | 21 | 15 | 0.51 |  |  |
| FBgn0020399 | Mst89B | 0.00689695 | 22 | 6 | 1.92 |  |  |
| FBgn0020412 | JIL-1 | 0.01441656 | 24 | 14 | 0.52 |  |  |
| FBgn0020633 | Mcm7 | 0.00705869 | 21 | 18 | 1.41 |  |  |
| FBgn0020756 | DNApol-epsilon | 0.01340298 | 25 | 17 | 0.74 |  |  |
| FBgn0021818 | cnk | 0.00348614 | 27 | 15 | 0.51 |  |  |
| FBgn0022768 | Pp2C1 | 0.00992957 | 22 | 18 | 0.50 | yes |  |
| FBgn0022770 | Peritrophin-A | 0.01333284 | 24 | 18 | 0.87 |  |  |
| FBgn0022960 | vimar | 0.01545791 | 21 | 18 | 0.65 | yes |  |
| FBgn0022986 | qkr58E-1 | 0.00267229 | 22 | 19 | 1.64 |  |  |
| FBgn0022987 | qkr54B | 0.00260812 | 20 | 18 | 0.94 |  |  |
| FBgn0023001 | melt | 0.01301668 | 24 | 16 | 0.68 |  |  |
| FBgn0023076 | Clk | 0.01311368 | 21 | 20 | 1.12 | yes |  |
| FBgn0023083 | fray | 0.01765278 | 20 | 19 | 0.59 |  |  |
| FBgn0023172 | RhoGEF2 | 0.01964923 | 26 | 13 | 0.53 | yes |  |
| FBgn0023518 | trr | 0.01056661 | 27 | 15 | 0.67 | yes |  |
| FBgn0024187 | syd | 0.00979911 | 22 | 17 | 0.57 |  |  |
| FBgn0024189 | sns | 0.00594751 | 22 | 18 | 1.32 |  |  |
| FBgn0024277 | trio | 0.01645765 | 26 | 14 | 0.55 | yes |  |
| FBgn0024290 | Slob | 0.02024825 | 20 | 12 | 0.91 | yes |  |
| FBgn0024806 | DIP2 | 0.01184899 | 26 | 14 | 0.55 | yes |  |
| FBgn0024897 | b6 | 0.00395975 | 21 | 21 | 1.39 |  |  |
| FBgn0024921 | Trn | 0.01150904 | 22 | 18 | 0.90 | yes | yes |
| FBgn0024975 | CG2712 | 0.01620029 | 21 | 19 | 0.59 |  |  |
| FBgn0024983 | CG4293 | 0.00256153 | 21 | 19 | 0.72 |  |  |
| FBgn0024994 | Csat | 0.01563859 | 23 | 17 | 0.58 |  |  |
| FBgn0024997 | CG2681 | 0.02020974 | 26 | 10 | 0.56 |  |  |
| FBgn0025185 | az2 | 0.0022695 | 21 | 19 | 0.73 |  |  |
| FBgn0025684 | CG15438 | 0.00350444 | 20 | 19 | 0.51 |  |  |
| FBgn0025741 | plexA | 0.01114393 | 28 | 12 | 0.66 | yes |  |
| FBgn0025809 | Paf-AHalpha | 0.01744876 | 28 | 25 | 0.83 |  |  |
| FBgn0025821 | I-t | 0.00791389 | 21 | 7 | 1.37 |  |  |
| FBgn0025836 | RhoGAP1A | 0.01755727 | 24 | 15 | 0.70 |  |  |
| FBgn0025864 | Crag | 0.01419605 | 22 | 16 | 0.54 | yes |  |
| FBgn0026085 | dgt4 | 0.0163032 | 21 | 21 | 2.66 | yes |  |
| FBgn0026136 | CkIIbeta2 | 0.01487039 | 21 | 7 | 1.22 |  |  |
| FBgn0026160 | tna | 0.00380166 | 21 | 7 | 1.69 | yes |  |
| FBgn0026319 | Traf4 | 0.01978341 | 21 | 17 | 0.58 |  |  |
| FBgn0026563 | CG1979 | 0.00429583 | 20 | 8 | 1.24 |  |  |
| FBgn0026582 | CG9418 | 0.0111008 | 27 | 25 | 0.72 |  |  |
| FBgn0027074 | CG17324 | 0.01128807 | 24 | 19 | 1.35 |  |  |
| FBgn0027095 | Manf | 0.01818928 | 27 | 27 | 0.97 |  |  |
| FBgn0027500 | spd-2 | 0.00734473 | 24 | 16 | 0.63 |  |  |
| FBgn0027513 | ana2 | 0.00297081 | 22 | 21 | 0.52 |  |  |
| FBgn0027584 | CG4757 | 0.00156162 | 23 | 23 | 0.59 |  |  |
| FBgn0027621 | Pfrx | 0.00336527 | 24 | 16 | 0.54 |  |  |
| FBgn0028292 | ric8a | 0.01377904 | 21 | 19 | 0.64 |  |  |
| FBgn0028379 | fan | 0.01520833 | 21 | 8 | 1.17 |  |  |
| FBgn0028401 | Snap24 | 0.00041858 | 20 | 19 | 0.60 |  |  |
| FBgn0028412 | Mst33A | 0.00664213 | 21 | 7 | 1.62 |  |  |
| FBgn0028418 | Lk | 0.02064974 | 21 | 1 | 1.06 |  |  |
| FBgn0028426 | JhI-1 | 0.00366984 | 20 | 18 | 0.75 |  |  |
| FBgn0028470 | Patr-1 | 0.01583808 | 21 | 18 | 0.90 |  |  |
| FBgn0028509 | cenG1A | 0.00599706 | 21 | 19 | 0.64 |  |  |
| FBgn0028523 | CG5888 | 0.00699319 | 24 | 18 | 0.94 |  |  |
| FBgn0028531 | CG15286 | 0.00241989 | 21 | 8 | 2.34 |  |  |
| FBgn0028533 | CG7953 | 0.01501361 | 21 | 10 | 2.46 |  |  |
| FBgn0028544 | CG16884 | 0.01052624 | 24 | 15 | 1.31 |  |  |
| FBgn0028560 | sut4 | 0.00252676 | 21 | 8 | 1.82 |  |  |
| FBgn0028675 | Sur | 0.01379678 | 24 | 16 | 1.12 | yes |  |
| FBgn0028704 | Nckx30C | 0.00269496 | 20 | 16 | 0.67 |  | yes |
| FBgn0028838 | CSN1a | 0.00383388 | 21 | 7 | 1.70 |  |  |
| FBgn0028847 | CG9014 | 0.01396616 | 22 | 6 | 1.21 |  |  |
| FBgn0028848 | CG7311 | 0.00177053 | 21 | 9 | 1.77 |  |  |
| FBgn0028850 | CG15260 | 0.00045706 | 20 | 8 | 1.15 |  |  |
| FBgn0028855 | CG15282 | 0.01103446 | 23 | 2 | 1.03 |  |  |
| FBgn0028857 | CG12448 | 0.00110309 | 20 | 8 | 1.42 |  |  |
| FBgn0028879 | CG15270 | 0.01676533 | 27 | 14 | 0.82 | yes |  |
| FBgn0028880 | CG15256 | 0.00915191 | 21 | 6 | 2.44 |  |  |
| FBgn0028903 | CG13243 | 0.00554073 | 20 | 8 | 1.26 |  |  |
| FBgn0028919 | CG16865 | 0.00687129 | 28 | 27 | 0.60 |  |  |
| FBgn0028920 | CG8997 | 0.00526133 | 21 | 9 | 1.37 |  |  |
| FBgn0028932 | CG16890 | 0.01045965 | 20 | 18 | 0.63 |  |  |
| FBgn0028942 | CG16852 | 0.01444752 | 21 | 7 | 2.02 |  |  |
| FBgn0028963 | Or49b | 0.01046021 | 22 | 19 | 1.21 |  |  |
| FBgn0028997 | nmdyn-D7 | 0.00521703 | 20 | 7 | 1.39 |  |  |
| FBgn0029002 | miple2 | 0.01074938 | 26 | 18 | 0.66 | yes |  |
| FBgn0029003 | mab-21 | 0.00731239 | 22 | 17 | 0.52 |  |  |
| FBgn0029117 | Surf1 | 0.00020541 | 23 | 22 | 0.60 | yes |  |
| FBgn0029137 | Patsas | 0.02083317 | 27 | 14 | 0.58 | yes |  |
| FBgn0029170 | TwdlT | 0.00670456 | 20 | 20 | 1.44 |  |  |
| FBgn0029661 | CG16781 | 0.00768953 | 20 | 7 | 2.16 |  |  |
| FBgn0029672 | CG2875 | 0.01191349 | 21 | 19 | 0.68 |  |  |
| FBgn0029686 | CG2941 | 0.01103399 | 21 | 19 | 0.71 |  |  |
| FBgn0029693 | CG6379 | 0.00623678 | 21 | 18 | 0.55 | yes |  |
| FBgn0029713 | CG11436 | 0.01364837 | 21 | 19 | 1.14 | yes |  |
| FBgn0029752 | TrxT | 0.01068898 | 21 | 8 | 3.93 | yes |  |
| FBgn0029761 | SK | 0.0152499 | 25 | 18 | 0.91 | yes |  |
| FBgn0029813 | CG15766 | 0.01892032 | 20 | 19 | 0.63 |  |  |
| FBgn0029825 | CG12728 | 0.00561662 | 20 | 18 | 0.98 |  |  |
| FBgn0029834 | CG5937 | 0.00370925 | 23 | 18 | 0.93 |  |  |
| FBgn0029863 | CG3823 | 0.00622915 | 21 | 21 | 0.75 | yes |  |
| FBgn0029874 | CG3342 | 0.01413092 | 21 | 18 | 0.52 |  |  |
| FBgn0029877 | CG17717 | 0.0116657 | 20 | 7 | 2.24 |  |  |
| FBgn0029894 | CG14440 | 0.01511515 | 22 | 18 | 0.76 |  |  |
| FBgn0029915 | CG14434 | 0.00016067 | 20 | 0 | 0.56 |  |  |
| FBgn0029955 | CG15478 | 0.00264033 | 23 | 19 | 0.83 |  |  |
| FBgn0029995 | CG2256 | 0.00242912 | 20 | 20 | 1.85 |  |  |
| FBgn0030010 | CG10959 | 0.00095571 | 22 | 0 | 0.60 |  |  |
| FBgn0030041 | CG12116 | 0.01938021 | 24 | 5 | 0.51 | yes |  |
| FBgn0030055 | CG12772 | 0.01241529 | 21 | 18 | 0.79 | yes |  |
| FBgn0030079 | CG7267 | 0.00087823 | 23 | 2 | 0.54 |  |  |
| FBgn0030100 | CG12106 | 0.00683949 | 20 | 17 | 0.66 |  |  |
| FBgn0030104 | CG15368 | 0.00668865 | 25 | 0 | 0.81 |  |  |
| FBgn0030178 | CG2974 | 0.01153702 | 21 | 19 | 0.61 |  |  |
| FBgn0030191 | CG15306 | 0.00267363 | 21 | 8 | 2.14 |  |  |
| FBgn0030218 | CG1628 | 0.00346894 | 21 | 18 | 0.52 | yes |  |
| FBgn0030241 | feo | 0.00847878 | 28 | 25 | 1.09 |  |  |
| FBgn0030243 | CG2186 | 0.01144264 | 24 | 16 | 0.82 | yes |  |
| FBgn0030252 | Myo10A | 0.01299236 | 24 | 18 | 1.00 |  |  |
| FBgn0030260 | CG1537 | 0.00161509 | 20 | 18 | 0.84 |  |  |
| FBgn0030272 | CG15201 | 0.01753555 | 24 | 2 | 0.57 |  |  |
| FBgn0030296 | CG15196 | 0.0129939 | 20 | 8 | 1.38 |  |  |
| FBgn0030339 | Cyp28c1 | 0.01457373 | 22 | 18 | 0.96 |  |  |
| FBgn0030356 | CR32661 | 0.00364573 | 20 | 8 | 2.75 |  |  |
| FBgn0030361 | CG1492 | 0.01834534 | 21 | 19 | 0.71 |  |  |
| FBgn0030376 | CG2750 | 0.00067806 | 21 | 8 | 1.85 |  |  |
| FBgn0030418 | CG4004 | 0.00415112 | 20 | 19 | 1.43 | yes |  |
| FBgn0030421 | CG3812 | 0.00496493 | 22 | 19 | 1.93 |  |  |
| FBgn0030466 | CG15744 | 0.0009731 | 20 | 18 | 0.59 |  |  |
| FBgn0030479 | Rbp1-like | 0.0006824 | 21 | 17 | 0.66 | yes |  |
| FBgn0030502 | tth | 0.00796021 | 21 | 18 | 0.58 |  |  |
| FBgn0030508 | CG15760 | 0.00301241 | 21 | 18 | 1.21 | yes |  |
| FBgn0030562 | CG9400 | 0.00561891 | 22 | 19 | 0.83 |  |  |
| FBgn0030641 | CG6299 | 9.6963E-06 | 21 | 18 | 1.00 |  |  |
| FBgn0030646 | CG9203 | 0.01130832 | 23 | 21 | 1.31 | yes |  |
| FBgn0030654 | CG15643 | 0.01900888 | 20 | 19 | 0.85 |  |  |
| FBgn0030671 | CG8134 | 0.01114674 | 21 | 18 | 0.53 |  |  |
| FBgn0030683 | CG8239 | 0.00030495 | 21 | 19 | 0.50 | yes |  |
| FBgn0030700 | CG15914 | 0.01138042 | 20 | 19 | 1.00 |  |  |
| FBgn0030763 | CG9782 | 0.00148635 | 22 | 18 | 1.20 |  |  |
| FBgn0030773 | CG9676 | 0.01247528 | 20 | 19 | 0.73 |  |  |
| FBgn0030780 | CG13008 | 0.00587672 | 20 | 8 | 3.26 |  |  |
| FBgn0030794 | CG13005 | 0.0018743 | 21 | 19 | 0.74 |  |  |
| FBgn0030809 | CG9086 | 0.01916947 | 24 | 17 | 0.83 |  |  |
| FBgn0030813 | CG4949 | 0.0123125 | 21 | 18 | 1.19 |  |  |
| FBgn0030884 | CG6847 | 0.00389373 | 21 | 19 | 0.81 |  |  |
| FBgn0030921 | CG6290 | 0.00799392 | 20 | 1 | 1.42 |  |  |
| FBgn0030927 | CG15046 | 0.01464486 | 20 | 19 | 0.93 |  |  |
| FBgn0030930 | GalNAc-T2 | 0.00249335 | 21 | 19 | 0.55 |  |  |
| FBgn0030936 | CG6481 | 0.02052503 | 27 | 9 | 2.85 |  |  |
| FBgn0030963 | CG7101 | 0.01039214 | 20 | 19 | 0.78 |  |  |
| FBgn0031053 | CG14223 | 0.00396976 | 22 | 20 | 1.00 |  |  |
| FBgn0031070 | CG12702 | 0.00116395 | 21 | 2 | 1.78 |  |  |
| FBgn0031080 | CG12655 | 0.00632743 | 20 | 19 | 1.36 | yes |  |
| FBgn0031111 | Obp19c | 0.00841212 | 22 | 20 | 0.59 |  |  |
| FBgn0031116 | CG1695 | 0.02091845 | 22 | 18 | 1.46 | yes |  |
| FBgn0031118 | RhoGAP19D | 0.01635981 | 22 | 17 | 0.75 |  |  |
| FBgn0031129 | CG1324 | 0.00901087 | 20 | 8 | 2.29 |  |  |
| FBgn0031139 | CG11227 | 0.00229849 | 21 | 8 | 1.55 | yes |  |
| FBgn0031183 | CG14621 | 0.00480793 | 21 | 19 | 0.58 |  |  |
| FBgn0031184 | CG14615 | 0.01356183 | 20 | 18 | 0.58 |  |  |
| FBgn0031208 | CG11023 | 0.0054617 | 20 | 9 | 2.24 |  |  |
| FBgn0031240 | CG3345 | 0.00241804 | 20 | 8 | 1.80 |  |  |
| FBgn0031279 | CG3544 | 0.00687931 | 22 | 7 | 1.82 |  |  |
| FBgn0031284 | CG3876 | 0.01922023 | 21 | 18 | 0.57 |  |  |
| FBgn0031298 | Atg4 | 0.01373849 | 28 | 15 | 0.57 |  |  |
| FBgn0031313 | CG5080 | 0.01009484 | 20 | 10 | 0.55 |  |  |
| FBgn0031332 | CG5556 | 0.01475958 | 20 | 8 | 2.32 |  |  |
| FBgn0031345 | CG18132 | 0.00554746 | 21 | 8 | 2.73 |  |  |
| FBgn0031367 | c-cup | 0.00383681 | 20 | 8 | 1.17 | yes |  |
| FBgn0031372 | CG7295 | 0.00301655 | 20 | 8 | 2.12 |  |  |
| FBgn0031428 | CG9886 | 0.01143868 | 22 | 19 | 0.56 | yes |  |
| FBgn0031443 | Prosbeta4R2 | 0.00173045 | 20 | 8 | 1.44 |  |  |
| FBgn0031444 | CG9879 | 0.0024621 | 24 | 5 | 1.58 |  |  |
| FBgn0031451 | CG9961 | 0.00051675 | 21 | 7 | 1.50 |  |  |
| FBgn0031457 | CG3077 | 0.00225495 | 21 | 18 | 0.85 | yes |  |
| FBgn0031501 | CG17261 | 0.01649732 | 21 | 7 | 1.90 |  |  |
| FBgn0031517 | CG15406 | 0.00364005 | 21 | 9 | 1.19 |  |  |
| FBgn0031540 | CG3238 | 0.0209 | 24 | 18 | 1.34 |  |  |
| FBgn0031542 | CG15414 | 0.00948205 | 26 | 14 | 0.65 | yes |  |
| FBgn0031564 | CG2816 | 0.00965429 | 23 | 21 | 0.84 |  |  |
| FBgn0031571 | CG3921 | 0.01637373 | 23 | 18 | 0.83 |  |  |
| FBgn0031574 | CG3964 | 0.00272425 | 21 | 8 | 1.82 | yes |  |
| FBgn0031591 | CG15425 | 0.01646056 | 20 | 8 | 1.55 |  |  |
| FBgn0031620 | CG11929 | 0.01735902 | 21 | 8 | 2.33 |  |  |
| FBgn0031631 | CG3225 | 0.00970385 | 24 | 16 | 0.61 |  |  |
| FBgn0031632 | CG15628 | 0.01574613 | 20 | 18 | 0.86 | yes |  |
| FBgn0031646 | CG2837 | 0.00023662 | 22 | 21 | 1.05 |  |  |
| FBgn0031682 | CG5828 | 0.01640925 | 21 | 19 | 0.73 | yes |  |
| FBgn0031690 | CG7742 | 0.00532366 | 21 | 7 | 2.02 |  |  |
| FBgn0031696 | Bub1 | 0.00337057 | 20 | 18 | 0.98 | yes |  |
| FBgn0031722 | CG14011 | 0.01164634 | 22 | 7 | 1.99 |  |  |
| FBgn0031723 | CG7251 | 0.00250675 | 20 | 8 | 1.56 |  |  |
| FBgn0031728 | Hsp60C | 0.00203382 | 21 | 8 | 1.26 |  |  |
| FBgn0031738 | CG9171 | 0.00340677 | 22 | 19 | 1.43 | yes |  |
| FBgn0031752 | CG9044 | 0.00642908 | 27 | 14 | 0.55 | yes |  |
| FBgn0031766 | CG9117 | 0.01493142 | 22 | 19 | 0.53 |  |  |
| FBgn0031784 | CG9222 | 0.0046832 | 21 | 7 | 1.54 |  |  |
| FBgn0031786 | CG13989 | 0.008726 | 21 | 7 | 1.86 |  |  |
| FBgn0031801 | CG9498 | 0.01089983 | 27 | 13 | 0.50 |  |  |
| FBgn0031834 | CG13766 | 0.00886892 | 22 | 18 | 0.63 |  |  |
| FBgn0031859 | CG17377 | 0.01698426 | 22 | 7 | 2.05 |  |  |
| FBgn0031861 | CG17375 | 0.02004931 | 22 | 5 | 1.14 |  |  |
| FBgn0031866 | neuroligin | 0.00095149 | 22 | 18 | 0.66 | yes |  |
| FBgn0031875 | CG3430 | 0.00189734 | 21 | 18 | 0.89 |  |  |
| FBgn0031941 | CG7211 | 0.00107272 | 20 | 8 | 2.28 |  |  |
| FBgn0031944 | CG7196 | 0.00139963 | 21 | 7 | 1.89 |  |  |
| FBgn0031971 | CG7224 | 0.00454885 | 21 | 1 | 0.94 | yes |  |
| FBgn0031976 | CG7367 | 0.00287797 | 23 | 19 | 0.55 |  |  |
| FBgn0031993 | CG8486 | 0.01850024 | 24 | 16 | 1.03 |  |  |
| FBgn0031999 | CG8419 | 0.00920884 | 28 | 13 | 0.51 |  |  |
| FBgn0032006 | Pvr | 0.00275486 | 26 | 14 | 0.89 | yes |  |
| FBgn0032022 | CG14275 | 0.00639901 | 21 | 16 | 0.66 | yes |  |
| FBgn0032039 | CG13385 | 0.01010335 | 21 | 7 | 2.66 |  |  |
| FBgn0032042 | CG13398 | 0.00739608 | 23 | 17 | 0.55 |  |  |
| FBgn0032049 | Bace | 0.00356688 | 20 | 9 | 2.32 |  |  |
| FBgn0032068 | CG9466 | 0.01956595 | 24 | 10 | 2.60 |  |  |
| FBgn0032079 | CG31886 | 0.01979458 | 21 | 17 | 0.84 |  |  |
| FBgn0032109 | CG17005 | 0.00991101 | 20 | 9 | 0.65 |  |  |
| FBgn0032111 | CG13110 | 0.01551506 | 20 | 8 | 1.94 |  |  |
| FBgn0032116 | CG3759 | 0.00629722 | 27 | 14 | 0.73 |  |  |
| FBgn0032131 | CG3841 | 0.01557602 | 22 | 9 | 2.10 |  |  |
| FBgn0032187 | CG4839 | 0.01660928 | 26 | 10 | 2.13 |  |  |
| FBgn0032219 | CG4995 | 0.00996328 | 21 | 8 | 1.42 |  |  |
| FBgn0032235 | CG5096 | 0.00986577 | 21 | 11 | 1.15 |  |  |
| FBgn0032244 | RfC3 | 0.01430153 | 20 | 19 | 1.12 | yes |  |
| FBgn0032281 | CG17107 | 0.01895223 | 20 | 19 | 2.38 |  |  |
| FBgn0032285 | CG17108 | 0.01220398 | 25 | 21 | 0.69 | yes |  |
| FBgn0032290 | CG6443 | 0.02048965 | 27 | 25 | 0.71 |  |  |
| FBgn0032299 | CG17127 | 0.0028297 | 21 | 21 | 2.15 |  |  |
| FBgn0032305 | CG6700 | 0.01080164 | 24 | 14 | 0.52 |  |  |
| FBgn0032314 | CG7309 | 0.00665522 | 20 | 8 | 2.66 |  |  |
| FBgn0032321 | YL-1 | 0.00254249 | 27 | 1 | 0.63 |  |  |
| FBgn0032329 | Art8 | 0.0012776 | 21 | 19 | 0.53 |  |  |
| FBgn0032330 | Samuel | 0.00746977 | 21 | 17 | 0.52 |  |  |
| FBgn0032360 | CG14926 | 0.00425015 | 20 | 8 | 1.54 |  |  |
| FBgn0032364 | CG4970 | 0.00664496 | 20 | 8 | 2.47 |  |  |
| FBgn0032372 | CG4988 | 0.00740707 | 20 | 7 | 2.11 |  |  |
| FBgn0032373 | Vha100-5 | 0.00844963 | 21 | 8 | 1.49 |  |  |
| FBgn0032385 | CG16964 | 0.00455241 | 20 | 9 | 3.72 |  |  |
| FBgn0032391 | escl | 0.00548967 | 22 | 20 | 0.96 |  |  |
| FBgn0032397 | Tom70 | 0.00037577 | 21 | 7 | 0.51 | yes |  |
| FBgn0032414 | CG17211 | 0.01371613 | 21 | 17 | 0.88 | yes |  |
| FBgn0032415 | rho-6 | 0.00409846 | 21 | 18 | 1.29 |  |  |
| FBgn0032422 | atilla | 0.0028176 | 28 | 27 | 0.76 |  |  |
| FBgn0032457 | CG15483 | 0.02003965 | 21 | 9 | 1.05 |  |  |
| FBgn0032464 | Vha68-3 | 0.00037774 | 20 | 9 | 1.76 |  |  |
| FBgn0032481 | CG16972 | 0.00187274 | 21 | 8 | 0.61 |  |  |
| FBgn0032503 | CG16825 | 0.01763559 | 23 | 5 | 1.88 |  |  |
| FBgn0032505 | CG16826 | 0.0206949 | 22 | 8 | 2.53 |  |  |
| FBgn0032506 | CG9395 | 0.01596132 | 24 | 19 | 0.71 | yes |  |
| FBgn0032514 | CG9302 | 0.00159144 | 20 | 1 | 0.77 |  |  |
| FBgn0032553 | CG4480 | 0.00249801 | 20 | 8 | 1.99 |  |  |
| FBgn0032601 | yellow-b | 0.00423166 | 21 | 20 | 0.68 |  |  |
| FBgn0032615 | CG6012 | 0.01709016 | 28 | 25 | 1.20 |  |  |
| FBgn0032632 | CG6380 | 0.00424646 | 20 | 8 | 1.50 |  |  |
| FBgn0032636 | CG5043 | 0.00599323 | 20 | 8 | 2.81 |  |  |
| FBgn0032664 | CG5755 | 0.00739954 | 21 | 7 | 2.13 |  |  |
| FBgn0032668 | CG17681 | 0.00852053 | 23 | 18 | 1.37 |  |  |
| FBgn0032669 | CG15155 | 0.00794703 | 22 | 10 | 1.04 |  |  |
| FBgn0032683 | kon | 0.02020299 | 20 | 18 | 0.67 |  |  |
| FBgn0032707 | CG10348 | 0.01342606 | 22 | 20 | 0.94 |  |  |
| FBgn0032769 | CG10750 | 0.00782269 | 21 | 7 | 1.35 |  |  |
| FBgn0032783 | CG10237 | 0.01659449 | 22 | 21 | 0.65 | yes |  |
| FBgn0032805 | CG10337 | 0.01806557 | 21 | 18 | 1.66 |  |  |
| FBgn0032847 | Taf13 | 0.00869889 | 26 | 2 | 0.66 |  |  |
| FBgn0032869 | CG17470 | 0.00058043 | 20 | 8 | 1.41 |  |  |
| FBgn0032876 | CG1962 | 0.0148231 | 21 | 19 | 0.87 | yes |  |
| FBgn0032878 | CG9316 | 0.00706756 | 21 | 8 | 2.54 |  |  |
| FBgn0032895 | CG9335 | 0.00715732 | 21 | 19 | 1.21 | yes |  |
| FBgn0032897 | CG9336 | 0.01419114 | 26 | 0 | 0.53 | yes |  |
| FBgn0032913 | CG9259 | 0.01215722 | 22 | 10 | 1.43 |  |  |
| FBgn0032921 | Mpp6 | 0.01499569 | 21 | 20 | 0.58 | yes |  |
| FBgn0032945 | CG8665 | 0.01763169 | 24 | 14 | 0.56 |  |  |
| FBgn0033032 | kune | 0.01676327 | 22 | 20 | 0.58 |  |  |
| FBgn0033039 | gp210 | 0.01153723 | 24 | 13 | 1.00 | yes |  |
| FBgn0033047 | CG7882 | 0.00662571 | 24 | 10 | 3.73 |  |  |
| FBgn0033049 | CG14471 | 0.01383359 | 21 | 19 | 1.06 |  |  |
| FBgn0033058 | CCHa2r | 0.01987828 | 21 | 18 | 1.05 |  |  |
| FBgn0033100 | CG3420 | 0.01363224 | 26 | 1 | 0.81 |  |  |
| FBgn0033115 | Spn42De | 0.00476276 | 23 | 18 | 1.19 |  |  |
| FBgn0033135 | Tsp42En | 0.00467967 | 21 | 18 | 0.55 | yes |  |
| FBgn0033159 | Dscam | 0.00682196 | 27 | 13 | 1.02 |  |  |
| FBgn0033185 | CG1603 | 0.01743185 | 20 | 19 | 0.65 |  |  |
| FBgn0033190 | CG2137 | 0.0019267 | 21 | 8 | 0.87 |  |  |
| FBgn0033221 | CG12825 | 0.00691976 | 20 | 11 | 0.84 |  |  |
| FBgn0033283 | CG11635 | 0.00663447 | 21 | 7 | 2.37 |  |  |
| FBgn0033289 | CG2121 | 0.00211284 | 26 | 14 | 0.80 | yes |  |
| FBgn0033297 | Mal-A8 | 0.0072147 | 21 | 9 | 3.16 |  |  |
| FBgn0033313 | Cirl | 0.01715176 | 28 | 13 | 0.62 |  |  |
| FBgn0033315 | beta3GalTII | 0.01729158 | 26 | 14 | 0.52 |  |  |
| FBgn0033368 | CG13743 | 0.01229186 | 24 | 15 | 1.37 | yes |  |
| FBgn0033387 | CG8008 | 0.0066894 | 28 | 17 | 0.51 |  |  |
| FBgn0033467 | CG15863 | 0.01659927 | 28 | 25 | 1.63 |  |  |
| FBgn0033500 | CG12913 | 0.01380235 | 23 | 18 | 0.93 |  |  |
| FBgn0033504 | CAP | 0.00032492 | 21 | 18 | 0.52 | yes |  |
| FBgn0033554 | Lsm10 | 0.00317825 | 20 | 0 | 1.40 |  |  |
| FBgn0033579 | CG13229 | 0.01621652 | 23 | 17 | 1.04 | yes |  |
| FBgn0033628 | CG13203 | 0.00367503 | 22 | 18 | 0.83 |  |  |
| FBgn0033631 | CG9027 | 0.019403 | 21 | 7 | 0.56 | yes |  |
| FBgn0033654 | Sobp | 0.00886786 | 21 | 16 | 0.78 |  |  |
| FBgn0033677 | CG8321 | 0.01642063 | 21 | 18 | 0.69 | yes |  |
| FBgn0033705 | CG13168 | 0.01586751 | 21 | 8 | 1.76 |  |  |
| FBgn0033756 | CG17760 | 0.01537094 | 24 | 19 | 1.23 |  |  |
| FBgn0033760 | CG8785 | 0.00812563 | 24 | 17 | 0.71 |  |  |
| FBgn0033794 | CG13326 | 0.00148892 | 20 | 8 | 1.01 |  |  |
| FBgn0033815 | CG4676 | 0.0178095 | 21 | 18 | 0.76 |  |  |
| FBgn0033818 | CG4712 | 0.00020456 | 21 | 9 | 1.67 |  |  |
| FBgn0033868 | S-Lap7 | 0.00283707 | 21 | 8 | 1.49 |  |  |
| FBgn0033880 | CG6553 | 0.00740782 | 21 | 19 | 1.82 |  |  |
| FBgn0033893 | CG18371 | 0.01281635 | 22 | 6 | 2.18 |  |  |
| FBgn0033936 | CG17386 | 0.0059013 | 20 | 20 | 1.27 | yes |  |
| FBgn0033952 | Adgf-E | 0.00063976 | 20 | 8 | 1.53 |  |  |
| FBgn0033963 | CG12857 | 0.00012734 | 20 | 9 | 1.47 |  |  |
| FBgn0033980 | Cyp6a20 | 0.00249355 | 21 | 20 | 0.53 | yes |  |
| FBgn0033981 | Cyp6a21 | 0.00614185 | 20 | 1 | 0.54 | yes |  |
| FBgn0034032 | CG8195 | 0.01176026 | 23 | 17 | 0.59 |  |  |
| FBgn0034071 | CG8405 | 0.00481887 | 21 | 18 | 0.69 | yes |  |
| FBgn0034075 | Asph | 0.01057686 | 27 | 11 | 0.89 |  |  |
| FBgn0034121 | CG6262 | 0.00294024 | 21 | 8 | 1.45 |  |  |
| FBgn0034132 | S-Lap8 | 0.00215103 | 20 | 9 | 1.72 |  |  |
| FBgn0034139 | CG4927 | 0.00200288 | 22 | 18 | 0.63 |  |  |
| FBgn0034144 | CG5089 | 0.00704492 | 20 | 8 | 1.47 |  |  |
| FBgn0034155 | unc-104 | 0.01826083 | 28 | 14 | 0.85 |  |  |
| FBgn0034172 | CG6665 | 0.01400415 | 20 | 18 | 0.70 |  |  |
| FBgn0034261 | HPS4 | 0.00492646 | 20 | 18 | 0.69 | yes |  |
| FBgn0034264 | CG10933 | 0.00556211 | 23 | 21 | 0.52 |  |  |
| FBgn0034314 | nopo | 0.0199342 | 24 | 18 | 0.58 |  |  |
| FBgn0034330 | CG18107 | 0.01332998 | 23 | 5 | 0.73 |  |  |
| FBgn0034390 | CG15093 | 0.00253118 | 27 | 3 | 0.66 | yes |  |
| FBgn0034438 | CG9416 | 0.01432428 | 26 | 14 | 0.53 |  |  |
| FBgn0034439 | CG10062 | 0.0021899 | 28 | 14 | 0.61 |  |  |
| FBgn0034440 | CG10073 | 0.00174309 | 20 | 1 | 1.68 |  |  |
| FBgn0034447 | CG7744 | 0.00269741 | 21 | 21 | 0.77 |  |  |
| FBgn0034459 | CG16716 | 0.00208111 | 21 | 8 | 1.87 | yes |  |
| FBgn0034467 | CG15128 | 0.00446626 | 21 | 7 | 2.30 |  |  |
| FBgn0034472 | CG8517 | 0.00701538 | 21 | 7 | 1.72 |  |  |
| FBgn0034477 | CG13872 | 0.00187666 | 22 | 8 | 1.20 |  |  |
| FBgn0034479 | CG8654 | 0.00632825 | 27 | 4 | 0.54 |  |  |
| FBgn0034491 | CG11055 | 0.00461341 | 28 | 14 | 0.63 |  |  |
| FBgn0034567 | CG15651 | 0.00648066 | 22 | 18 | 0.73 |  |  |
| FBgn0034605 | CG15661 | 0.01901599 | 24 | 12 | 1.22 |  |  |
| FBgn0034658 | Grx-1 | 0.00568527 | 21 | 8 | 1.24 |  |  |
| FBgn0034684 | CG13501 | 0.00028518 | 20 | 9 | 0.85 |  |  |
| FBgn0034715 | Oatp58Db | 0.00103306 | 22 | 9 | 1.01 |  |  |
| FBgn0034826 | CG13544 | 0.00933624 | 20 | 8 | 3.09 |  |  |
| FBgn0034835 | CG3092 | 0.01731569 | 21 | 7 | 2.26 |  |  |
| FBgn0034840 | CG3124 | 0.00264255 | 20 | 9 | 1.82 |  |  |
| FBgn0034882 | CG5398 | 0.00542392 | 21 | 8 | 1.94 |  |  |
| FBgn0034897 | Sesn | 0.00920542 | 26 | 13 | 0.65 |  |  |
| FBgn0034996 | CG13575 | 0.00040571 | 22 | 19 | 1.31 | yes |  |
| FBgn0034998 | CG13577 | 0.01946914 | 22 | 7 | 1.75 |  |  |
| FBgn0035034 | CG3565 | 0.00312472 | 21 | 9 | 1.84 |  |  |
| FBgn0035047 | Pof | 0.0018622 | 20 | 8 | 1.07 |  |  |
| FBgn0035084 | CG15861 | 0.00344976 | 22 | 19 | 0.93 |  |  |
| FBgn0035085 | CG3770 | 0.00664173 | 21 | 18 | 0.97 | yes |  |
| FBgn0035106 | rno | 0.00322923 | 22 | 17 | 1.03 |  |  |
| FBgn0035113 | pyx | 0.00335356 | 23 | 15 | 0.72 |  |  |
| FBgn0035124 | ttm2 | 0.01216561 | 21 | 7 | 1.95 |  |  |
| FBgn0035132 | mthl10 | 0.00721796 | 22 | 18 | 0.51 |  |  |
| FBgn0035155 | RabX6 | 0.01015434 | 21 | 19 | 0.75 | yes |  |
| FBgn0035161 | CG13898 | 0.0177451 | 21 | 7 | 1.57 |  |  |
| FBgn0035199 | CG9134 | 0.0124874 | 20 | 17 | 0.73 |  |  |
| FBgn0035260 | CG7991 | 0.02069759 | 22 | 18 | 0.72 | yes |  |
| FBgn0035265 | CG18173 | 0.00326135 | 27 | 9 | 1.64 |  |  |
| FBgn0035273 | CG12020 | 0.00508046 | 20 | 8 | 1.25 |  |  |
| FBgn0035279 | Cpr62Ba | 0.00504288 | 20 | 19 | 1.19 |  |  |
| FBgn0035285 | CG12025 | 0.0058138 | 21 | 19 | 0.57 |  |  |
| FBgn0035331 | DmsR-1 | 0.00429359 | 21 | 19 | 1.10 |  |  |
| FBgn0035359 | CG1143 | 0.01425113 | 28 | 24 | 0.58 |  |  |
| FBgn0035379 | spz5 | 0.01317392 | 27 | 23 | 0.89 |  |  |
| FBgn0035382 | Or63a | 0.0187617 | 24 | 15 | 0.88 |  |  |
| FBgn0035398 | Cht7 | 0.01160846 | 26 | 17 | 1.04 |  |  |
| FBgn0035414 | CG14965 | 0.00098082 | 25 | 24 | 0.76 | yes |  |
| FBgn0035445 | CG12014 | 0.01595136 | 24 | 15 | 0.62 | yes |  |
| FBgn0035455 | CG10862 | 0.01053044 | 20 | 8 | 1.63 |  |  |
| FBgn0035477 | CG14982 | 0.00791368 | 22 | 18 | 0.94 | yes |  |
| FBgn0035497 | CG14995 | 0.00684322 | 20 | 8 | 1.28 |  |  |
| FBgn0035534 | mRpS6 | 0.01059775 | 28 | 26 | 1.30 |  |  |
| FBgn0035598 | CG4669 | 0.00200433 | 21 | 8 | 2.31 |  |  |
| FBgn0035625 | Blimp-1 | 0.0123497 | 21 | 18 | 0.61 |  |  |
| FBgn0035665 | Jon65Aiii | 0.01058669 | 23 | 7 | 1.86 |  |  |
| FBgn0035676 | CG6619 | 0.00017242 | 21 | 20 | 0.98 |  |  |
| FBgn0035677 | CG13293 | 0.01913413 | 24 | 20 | 1.06 |  |  |
| FBgn0035689 | CG7376 | 0.01409891 | 22 | 17 | 0.96 | yes |  |
| FBgn0035695 | CG10226 | 0.01424277 | 25 | 14 | 0.88 |  |  |
| FBgn0035702 | CG10147 | 0.00394052 | 22 | 18 | 1.15 |  |  |
| FBgn0035703 | CG8270 | 0.00869636 | 21 | 18 | 0.60 |  |  |
| FBgn0035707 | CG8368 | 0.00986967 | 21 | 7 | 0.53 |  |  |
| FBgn0035709 | eIF4E-4 | 0.00360718 | 21 | 7 | 1.55 |  |  |
| FBgn0035724 | CG10064 | 0.00517172 | 20 | 7 | 1.85 |  |  |
| FBgn0035725 | Mis12 | 0.00107441 | 20 | 0 | 1.00 |  |  |
| FBgn0035776 | CG8564 | 0.02026721 | 22 | 6 | 2.10 |  |  |
| FBgn0035800 | CG7716 | 0.01781837 | 20 | 8 | 2.89 |  |  |
| FBgn0035823 | eIF4E-5 | 0.00282206 | 20 | 8 | 1.58 |  |  |
| FBgn0035833 | CG7565 | 0.01984099 | 25 | 17 | 0.73 |  |  |
| FBgn0035852 | CG7387 | 0.00460714 | 21 | 8 | 1.29 |  |  |
| FBgn0035855 | CG7366 | 0.00699815 | 20 | 9 | 2.19 |  |  |
| FBgn0035871 | CG7188 | 0.01596556 | 28 | 1 | 0.79 |  |  |
| FBgn0035891 | Oseg1 | 0.01041554 | 24 | 15 | 0.78 |  |  |
| FBgn0035892 | exo70 | 0.01145671 | 27 | 12 | 0.55 |  |  |
| FBgn0035895 | Unr | 0.00867204 | 22 | 18 | 0.90 |  |  |
| FBgn0035901 | CG6745 | 0.01292542 | 21 | 19 | 0.71 |  |  |
| FBgn0035902 | CG6683 | 0.00055761 | 21 | 0 | 0.78 |  |  |
| FBgn0035915 | S-Lap1 | 0.00241046 | 21 | 8 | 1.69 |  |  |
| FBgn0035957 | CG5144 | 0.01136076 | 20 | 7 | 1.76 |  |  |
| FBgn0035965 | Use1 | 0.01150804 | 20 | 19 | 0.67 |  |  |
| FBgn0035966 | nwk | 0.00793552 | 23 | 18 | 1.14 | yes |  |
| FBgn0035985 | Cpr67B | 0.00129025 | 26 | 24 | 0.58 |  |  |
| FBgn0035996 | CG3448 | 0.00585147 | 22 | 19 | 2.29 |  |  |
| FBgn0036031 | CG6761 | 0.0159621 | 22 | 9 | 1.70 |  |  |
| FBgn0036032 | CG16711 | 0.01511984 | 23 | 18 | 0.97 |  |  |
| FBgn0036040 | CG6749 | 0.00130958 | 23 | 18 | 0.89 | yes |  |
| FBgn0036063 | CG6674 | 0.01963081 | 21 | 3 | 0.57 |  |  |
| FBgn0036094 | CG14153 | 0.00041101 | 21 | 20 | 1.25 |  |  |
| FBgn0036110 | Cpr67Fb | 0.01229156 | 22 | 19 | 2.05 |  |  |
| FBgn0036125 | CG6279 | 0.00411046 | 20 | 8 | 0.86 |  |  |
| FBgn0036141 | wls | 0.00573849 | 21 | 20 | 0.95 |  |  |
| FBgn0036159 | CG7557 | 0.00048077 | 20 | 9 | 2.03 |  |  |
| FBgn0036162 | CG6140 | 0.00678589 | 20 | 9 | 1.88 |  |  |
| FBgn0036240 | CG6928 | 0.0102147 | 20 | 18 | 0.85 |  |  |
| FBgn0036259 | CG9760 | 0.00658245 | 22 | 19 | 1.94 |  |  |
| FBgn0036279 | Ncc69 | 0.01445813 | 25 | 14 | 0.70 |  |  |
| FBgn0036291 | CG10681 | 0.0115337 | 20 | 18 | 0.70 |  |  |
| FBgn0036292 | CG10646 | 0.01238715 | 22 | 18 | 0.57 |  |  |
| FBgn0036329 | CG11262 | 0.00590272 | 20 | 9 | 1.18 |  |  |
| FBgn0036360 | CG10713 | 0.00726073 | 21 | 20 | 0.55 |  |  |
| FBgn0036369 | CG10089 | 0.01740447 | 20 | 7 | 0.81 | yes |  |
| FBgn0036373 | CG10741 | 0.00889316 | 22 | 22 | 0.52 | yes |  |
| FBgn0036374 | Spt20 | 0.00482234 | 21 | 18 | 0.64 |  |  |
| FBgn0036396 | CG17359 | 0.00798279 | 21 | 17 | 1.12 |  |  |
| FBgn0036415 | CG7768 | 0.0126441 | 20 | 8 | 1.25 |  |  |
| FBgn0036437 | CG5048 | 0.00385312 | 21 | 8 | 1.37 |  |  |
| FBgn0036441 | CG13476 | 0.00838453 | 21 | 7 | 2.22 |  |  |
| FBgn0036442 | CG13473 | 0.01308525 | 20 | 7 | 2.42 |  |  |
| FBgn0036450 | CG13472 | 0.00872264 | 21 | 1 | 0.53 |  |  |
| FBgn0036465 | Rpn12R | 0.00670106 | 21 | 8 | 0.93 |  |  |
| FBgn0036520 | CG13449 | 0.01500973 | 20 | 8 | 1.15 |  |  |
| FBgn0036544 | sff | 0.0116694 | 21 | 14 | 0.77 |  |  |
| FBgn0036576 | CG5151 | 0.01380035 | 20 | 18 | 0.75 |  |  |
| FBgn0036587 | CG4950 | 0.01579577 | 23 | 19 | 1.31 |  |  |
| FBgn0036621 | roq | 0.01852225 | 20 | 18 | 0.77 |  |  |
| FBgn0036627 | CG4680 | 0.00301737 | 21 | 19 | 0.88 |  |  |
| FBgn0036648 | CG4098 | 0.00358297 | 20 | 18 | 1.19 |  |  |
| FBgn0036659 | CG9701 | 0.01465509 | 21 | 19 | 0.63 | yes | yes |
| FBgn0036703 | CG7707 | 0.00849106 | 21 | 9 | 2.85 |  |  |
| FBgn0036713 | Mip | 0.00083378 | 21 | 20 | 1.28 | yes |  |
| FBgn0036723 | CG12229 | 0.0057927 | 21 | 8 | 1.65 |  |  |
| FBgn0036729 | CG13733 | 0.0179254 | 22 | 7 | 2.32 |  |  |
| FBgn0036738 | CG7542 | 0.01827497 | 22 | 10 | 7.44 |  |  |
| FBgn0036750 | CG6034 | 0.00437598 | 20 | 17 | 0.98 |  |  |
| FBgn0036754 | CG5589 | 0.00209055 | 21 | 19 | 0.70 |  |  |
| FBgn0036773 | CG13698 | 0.00445347 | 20 | 18 | 0.87 | yes | yes |
| FBgn0036784 | CG5103 | 0.00685586 | 20 | 8 | 2.36 |  |  |
| FBgn0036785 | CG13700 | 0.02008444 | 21 | 8 | 1.46 |  |  |
| FBgn0036876 | CG9451 | 0.00277437 | 26 | 9 | 0.80 | yes |  |
| FBgn0036895 | CG9392 | 0.00902542 | 21 | 7 | 2.18 |  |  |
| FBgn0036906 | CG14102 | 0.0013535 | 21 | 18 | 1.23 |  |  |
| FBgn0036918 | CG7770 | 0.01072572 | 28 | 0 | 0.59 |  |  |
| FBgn0036931 | CG14183 | 0.00550842 | 21 | 8 | 2.38 |  |  |
| FBgn0036934 | sNPF-R | 0.01986136 | 22 | 19 | 0.76 |  |  |
| FBgn0036942 | CG7328 | 0.01611484 | 22 | 19 | 0.53 |  |  |
| FBgn0036960 | CG13814 | 0.00066671 | 21 | 18 | 1.21 |  |  |
| FBgn0036962 | CG17122 | 0.0085975 | 21 | 8 | 1.18 | yes |  |
| FBgn0036967 | CG6597 | 0.02030158 | 28 | 0 | 0.65 |  |  |
| FBgn0036969 | Spn77Bb | 0.00505711 | 23 | 10 | 1.41 |  |  |
| FBgn0036970 | Spn77Bc | 0.00103466 | 20 | 10 | 1.66 |  |  |
| FBgn0037035 | CG10589 | 0.00429895 | 20 | 8 | 3.14 |  |  |
| FBgn0037038 | CG11037 | 0.00394049 | 23 | 8 | 1.29 |  |  |
| FBgn0037076 | ebd2 | 0.00015584 | 20 | 18 | 0.72 |  |  |
| FBgn0037094 | CG7611 | 0.00693355 | 28 | 14 | 0.51 |  |  |
| FBgn0037101 | CG7634 | 0.01153158 | 21 | 7 | 2.41 |  |  |
| FBgn0037115 | CG11249 | 0.00837901 | 26 | 7 | 1.72 |  |  |
| FBgn0037141 | DNApol-eta | 0.00128904 | 20 | 19 | 0.94 |  |  |
| FBgn0037147 | CG7140 | 0.0083005 | 21 | 8 | 1.82 |  |  |
| FBgn0037164 | CG11438 | 0.00976074 | 26 | 24 | 0.76 |  |  |
| FBgn0037251 | CG9804 | 0.01687344 | 20 | 19 | 0.63 |  |  |
| FBgn0037265 | CG12001 | 0.00865497 | 22 | 19 | 0.56 |  |  |
| FBgn0037322 | Or83a | 0.00770551 | 21 | 18 | 1.08 |  |  |
| FBgn0037325 | CG12147 | 0.01065726 | 22 | 7 | 1.63 |  |  |
| FBgn0037364 | Rab23 | 3.4164E-05 | 20 | 19 | 0.71 |  |  |
| FBgn0037379 | CG10979 | 0.01306647 | 22 | 17 | 0.72 |  |  |
| FBgn0037396 | CG11459 | 0.00885244 | 21 | 18 | 0.73 |  |  |
| FBgn0037409 | CG15589 | 0.00377284 | 22 | 18 | 0.86 |  |  |
| FBgn0037445 | CG9727 | 0.00265522 | 23 | 19 | 1.37 |  |  |
| FBgn0037455 | CG2336 | 0.00536961 | 21 | 8 | 1.66 |  |  |
| FBgn0037460 | sowi | 0.00922074 | 21 | 8 | 2.04 |  |  |
| FBgn0037462 | sunz | 0.00256487 | 20 | 8 | 2.53 |  |  |
| FBgn0037492 | CG10050 | 0.01262497 | 25 | 22 | 0.79 |  |  |
| FBgn0037500 | CG17944 | 0.01706097 | 20 | 8 | 1.82 |  |  |
| FBgn0037504 | CG1142 | 0.01793467 | 26 | 25 | 0.66 |  |  |
| FBgn0037506 | CG1287 | 0.01011469 | 22 | 7 | 1.52 |  |  |
| FBgn0037517 | CG10086 | 0.00593609 | 23 | 9 | 1.30 |  |  |
| FBgn0037521 | CG2993 | 0.00844118 | 20 | 19 | 0.85 | yes |  |
| FBgn0037546 | CG7918 | 0.01075591 | 23 | 17 | 0.73 |  |  |
| FBgn0037556 | CG9636 | 0.00429079 | 21 | 17 | 0.70 |  |  |
| FBgn0037567 | Gfr | 0.01756143 | 22 | 19 | 0.65 |  |  |
| FBgn0037616 | CG8136 | 0.01672233 | 22 | 6 | 2.34 | yes |  |
| FBgn0037626 | CG8236 | 0.01338068 | 21 | 9 | 1.52 |  |  |
| FBgn0037635 | CG9837 | 0.00392193 | 27 | 17 | 0.58 |  |  |
| FBgn0037659 | Kdm2 | 0.01472793 | 27 | 14 | 0.51 |  |  |
| FBgn0037683 | CG18473 | 0.02094733 | 21 | 12 | 1.41 | yes |  |
| FBgn0037697 | CG9363 | 0.00289255 | 27 | 27 | 1.24 | yes |  |
| FBgn0037702 | CG8176 | 0.01948155 | 28 | 13 | 0.52 |  |  |
| FBgn0037741 | CG16908 | 0.00305843 | 26 | 14 | 0.53 |  |  |
| FBgn0037777 | CG11722 | 0.01777867 | 28 | 26 | 0.85 | yes |  |
| FBgn0037802 | Sirt6 | 0.00106978 | 20 | 18 | 0.89 |  |  |
| FBgn0037848 | Tsp86D | 0.01899673 | 21 | 17 | 0.68 |  |  |
| FBgn0037860 | CG6629 | 0.00045425 | 20 | 8 | 1.93 |  |  |
| FBgn0037876 | CG4820 | 0.01229214 | 28 | 25 | 0.89 | yes |  |
| FBgn0037915 | CG6790 | 0.00927283 | 21 | 8 | 1.84 |  |  |
| FBgn0037938 | CG14717 | 0.00400666 | 20 | 10 | 1.33 |  |  |
| FBgn0037956 | CG6959 | 0.01887425 | 21 | 18 | 0.61 |  |  |
| FBgn0037985 | ssp5 | 0.00783058 | 22 | 7 | 1.50 |  |  |
| FBgn0037995 | CG3809 | 0.00434754 | 20 | 8 | 1.27 |  |  |
| FBgn0038000 | CG10014 | 0.0030552 | 20 | 8 | 1.43 |  |  |
| FBgn0038034 | Cyp9f3Psi | 0.01909711 | 27 | 16 | 0.89 |  |  |
| FBgn0038052 | CG5538 | 0.00065102 | 21 | 8 | 1.43 |  |  |
| FBgn0038074 | CG6188 | 0.00242255 | 21 | 2 | 0.95 | yes |  |
| FBgn0038079 | CG14394 | 0.00878464 | 23 | 17 | 0.66 |  |  |
| FBgn0038102 | CG14383 | 0.01258897 | 21 | 7 | 1.58 |  |  |
| FBgn0038123 | CG8508 | 0.00253806 | 21 | 9 | 1.66 |  |  |
| FBgn0038124 | CG14380 | 0.01107412 | 21 | 9 | 2.34 |  |  |
| FBgn0038140 | CG8784 | 0.01618746 | 24 | 18 | 1.30 |  |  |
| FBgn0038165 | Task6 | 0.00258495 | 22 | 19 | 1.20 |  |  |
| FBgn0038200 | CG9920 | 0.01542652 | 21 | 7 | 2.52 |  |  |
| FBgn0038201 | Pk1r | 0.0064706 | 21 | 19 | 0.72 |  |  |
| FBgn0038248 | CG7886 | 0.0057406 | 20 | 8 | 1.71 |  |  |
| FBgn0038266 | CG3610 | 0.00307694 | 20 | 8 | 2.48 |  |  |
| FBgn0038268 | CG3631 | 0.01316967 | 21 | 18 | 0.54 |  |  |
| FBgn0038269 | Rrp6 | 0.00194121 | 21 | 8 | 0.59 |  |  |
| FBgn0038275 | CG3817 | 0.00321062 | 27 | 1 | 0.61 |  |  |
| FBgn0038295 | Gyc88E | 0.00412229 | 21 | 18 | 0.91 |  |  |
| FBgn0038326 | CG5044 | 0.01995352 | 22 | 18 | 0.59 |  |  |
| FBgn0038332 | CG6136 | 0.00974137 | 24 | 23 | 0.57 |  |  |
| FBgn0038334 | h-cup | 0.00608163 | 20 | 9 | 1.79 |  |  |
| FBgn0038341 | CG14869 | 0.00463322 | 26 | 14 | 0.91 |  |  |
| FBgn0038354 | CG5404 | 0.01774417 | 21 | 1 | 1.07 |  |  |
| FBgn0038371 | Pbp45 | 0.01565487 | 21 | 19 | 0.93 |  |  |
| FBgn0038377 | CG9632 | 0.00566771 | 21 | 8 | 1.51 |  |  |
| FBgn0038390 | Rbf2 | 0.00644087 | 21 | 19 | 0.75 |  |  |
| FBgn0038412 | Zip3 | 0.00335992 | 21 | 19 | 0.62 |  |  |
| FBgn0038436 | Gyc-89Db | 0.01313233 | 22 | 19 | 1.58 | yes |  |
| FBgn0038439 | Cad89D | 0.01781844 | 23 | 18 | 1.10 |  |  |
| FBgn0038445 | CG14891 | 0.00580619 | 20 | 9 | 1.35 |  |  |
| FBgn0038450 | CG17560 | 0.00994863 | 22 | 8 | 1.54 |  |  |
| FBgn0038533 | CG7523 | 0.0001625 | 21 | 20 | 0.74 |  |  |
| FBgn0038541 | TyrRII | 0.02009518 | 22 | 20 | 0.95 |  |  |
| FBgn0038607 | CG7669 | 0.00087689 | 21 | 8 | 1.47 |  |  |
| FBgn0038629 | CG14304 | 0.01117305 | 27 | 16 | 0.82 |  |  |
| FBgn0038630 | CG14305 | 0.00198261 | 20 | 9 | 1.42 |  |  |
| FBgn0038697 | CG3581 | 0.02033464 | 22 | 7 | 2.38 |  |  |
| FBgn0038706 | CG3517 | 0.00660324 | 20 | 8 | 1.32 |  |  |
| FBgn0038718 | CG17752 | 0.00204437 | 22 | 10 | 1.42 |  |  |
| FBgn0038762 | CG4836 | 0.00100376 | 20 | 8 | 1.61 |  |  |
| FBgn0038765 | CG4424 | 0.00650283 | 27 | 24 | 0.78 |  |  |
| FBgn0038769 | CG10889 | 0.00596588 | 21 | 19 | 1.01 |  |  |
| FBgn0038797 | Dic2 | 0.00100377 | 21 | 8 | 1.68 |  |  |
| FBgn0038819 | Cpr92F | 0.00888085 | 28 | 14 | 2.57 |  |  |
| FBgn0038826 | CG17838 | 0.01036633 | 22 | 7 | 1.23 |  |  |
| FBgn0038865 | CG10824 | 0.0018348 | 22 | 18 | 0.56 |  |  |
| FBgn0038909 | CG6569 | 0.00380625 | 21 | 9 | 1.17 |  |  |
| FBgn0038915 | CG17819 | 0.00445902 | 20 | 9 | 1.72 |  |  |
| FBgn0038917 | CG6678 | 0.01213184 | 22 | 17 | 0.81 |  |  |
| FBgn0038926 | CG13409 | 0.00556484 | 28 | 14 | 0.56 | yes |  |
| FBgn0038928 | BG4 | 0.00366055 | 21 | 19 | 0.64 |  |  |
| FBgn0038967 | CG13847 | 0.00887725 | 23 | 21 | 0.97 |  |  |
| FBgn0038978 | CG7045 | 0.00525143 | 21 | 7 | 1.73 |  |  |
| FBgn0039006 | Cyp6d4 | 0.00326527 | 21 | 17 | 0.98 |  |  |
| FBgn0039008 | CG6972 | 0.00100714 | 21 | 18 | 0.75 |  |  |
| FBgn0039010 | CG4907 | 0.00334453 | 21 | 7 | 1.86 |  |  |
| FBgn0039020 | CG17141 | 0.01384205 | 21 | 19 | 0.79 |  |  |
| FBgn0039028 | CG13840 | 0.00527064 | 21 | 20 | 1.34 |  |  |
| FBgn0039041 | CG13838 | 0.00731475 | 22 | 8 | 1.27 |  |  |
| FBgn0039070 | CG17083 | 0.00035523 | 20 | 8 | 1.45 |  |  |
| FBgn0039071 | CG4434 | 0.01853495 | 23 | 8 | 2.28 |  |  |
| FBgn0039083 | CG10177 | 0.00308204 | 20 | 9 | 1.43 |  |  |
| FBgn0039098 | CG13822 | 0.00134525 | 22 | 18 | 0.58 | yes |  |
| FBgn0039157 | Myo95E | 0.00419631 | 23 | 16 | 0.63 |  |  |
| FBgn0039159 | mRpS24 | 0.00373693 | 20 | 19 | 0.72 |  |  |
| FBgn0039160 | CG5510 | 0.00659675 | 21 | 20 | 0.85 |  |  |
| FBgn0039177 | CG13611 | 0.00932555 | 21 | 8 | 1.77 |  |  |
| FBgn0039190 | CG5762 | 0.00901672 | 20 | 8 | 2.17 |  |  |
| FBgn0039226 | Ude | 0.01207789 | 21 | 18 | 0.61 |  |  |
| FBgn0039235 | CG10899 | 0.0001163 | 21 | 8 | 1.42 |  |  |
| FBgn0039250 | CG11120 | 0.02028126 | 24 | 1 | 0.87 |  |  |
| FBgn0039255 | CG13646 | 0.00425969 | 20 | 18 | 0.76 |  |  |
| FBgn0039298 | to | 0.00526205 | 27 | 23 | 0.60 | yes |  |
| FBgn0039312 | CG10514 | 0.00333304 | 21 | 8 | 0.58 | yes |  |
| FBgn0039323 | CG10559 | 0.01045026 | 20 | 17 | 0.89 |  |  |
| FBgn0039324 | CG10553 | 0.00068671 | 21 | 19 | 0.69 | yes |  |
| FBgn0039343 | CG5111 | 0.00499428 | 22 | 7 | 2.09 |  |  |
| FBgn0039354 | Lgr3 | 0.01802116 | 26 | 17 | 1.26 |  |  |
| FBgn0039373 | CG5024 | 0.00860566 | 20 | 8 | 1.69 |  |  |
| FBgn0039376 | CG14354 | 0.00849732 | 22 | 8 | 1.96 |  |  |
| FBgn0039379 | CG5886 | 0.00810547 | 22 | 7 | 0.50 |  |  |
| FBgn0039407 | CG14544 | 0.00389303 | 22 | 19 | 0.78 |  |  |
| FBgn0039466 | CG5521 | 0.01183558 | 24 | 18 | 0.93 |  |  |
| FBgn0039471 | CG6295 | 0.01367681 | 22 | 9 | 1.52 |  |  |
| FBgn0039478 | Nep5 | 0.00991438 | 26 | 23 | 0.86 |  |  |
| FBgn0039488 | CG6066 | 0.01151181 | 20 | 18 | 0.61 |  |  |
| FBgn0039491 | CG6059 | 0.00061935 | 22 | 7 | 1.37 |  |  |
| FBgn0039501 | CG5987 | 0.00338665 | 21 | 8 | 1.79 | yes |  |
| FBgn0039589 | CG9986 | 0.01762897 | 23 | 20 | 0.70 |  |  |
| FBgn0039623 | CG1951 | 0.0176996 | 23 | 16 | 0.54 |  |  |
| FBgn0039641 | CG14511 | 0.00096902 | 20 | 19 | 0.57 | yes |  |
| FBgn0039644 | CG11897 | 0.0070487 | 27 | 13 | 0.80 | yes |  |
| FBgn0039667 | CG2010 | 0.00149184 | 23 | 19 | 0.58 |  |  |
| FBgn0039742 | CG15528 | 0.00129331 | 25 | 22 | 0.66 |  |  |
| FBgn0039792 | CG2267 | 0.00123368 | 21 | 8 | 1.69 |  |  |
| FBgn0039829 | CG15561 | 0.00642976 | 21 | 18 | 1.24 | yes |  |
| FBgn0039873 | Smvt | 0.00872494 | 21 | 9 | 2.33 |  |  |
| FBgn0039915 | CG1732 | 0.00167282 | 28 | 13 | 0.98 |  |  |
| FBgn0039925 | Kif3C | 0.01403069 | 21 | 19 | 1.79 |  |  |
| FBgn0039941 | CG17167 | 0.00607986 | 22 | 18 | 0.88 |  |  |
| FBgn0040001 | CG17374 | 0.00422 | 23 | 10 | 2.28 |  |  |
| FBgn0040022 | CG40351 | 0.01173706 | 23 | 13 | 0.50 |  |  |
| FBgn0040091 | Ugt58Fa | 0.008452 | 28 | 11 | 0.71 |  |  |
| FBgn0040207 | kat80 | 0.01494738 | 27 | 13 | 0.74 |  |  |
| FBgn0040212 | Dhap-at | 0.00981537 | 21 | 18 | 0.85 |  |  |
| FBgn0040297 | Nhe2 | 0.01287828 | 23 | 16 | 0.85 | yes |  |
| FBgn0040343 | CG3713 | 0.00946068 | 24 | 15 | 1.25 |  |  |
| FBgn0040344 | CG3711 | 0.01328045 | 24 | 16 | 0.59 |  |  |
| FBgn0040366 | CG11398 | 0.00223085 | 20 | 19 | 0.53 |  |  |
| FBgn0040368 | eIF4E-7 | 0.00603469 | 20 | 8 | 1.66 |  |  |
| FBgn0040371 | CG12470 | 0.01662754 | 21 | 7 | 2.18 |  |  |
| FBgn0040372 | G9a | 0.00673549 | 24 | 15 | 0.75 |  |  |
| FBgn0040388 | boi | 0.01317745 | 23 | 20 | 1.32 |  |  |
| FBgn0040392 | CG14050 | 0.01018647 | 27 | 19 | 1.03 |  |  |
| FBgn0040467 | Dip1 | 0.00547317 | 28 | 24 | 0.66 |  |  |
| FBgn0040505 | Alk | 0.01842859 | 22 | 19 | 1.67 |  |  |
| FBgn0040519 | CG15219 | 0.020033 | 22 | 7 | 1.71 |  |  |
| FBgn0040600 | CG13631 | 0.00313295 | 21 | 19 | 0.76 | yes |  |
| FBgn0040609 | CG3348 | 0.00408765 | 28 | 5 | 0.64 | yes |  |
| FBgn0040653 | IM4 | 0.01322954 | 26 | 0 | 0.79 |  |  |
| FBgn0040688 | CG12483 | 0.0052358 | 21 | 18 | 0.70 |  |  |
| FBgn0040694 | CG14974 | 0.00565854 | 20 | 8 | 3.55 |  |  |
| FBgn0040717 | Nplp4 | 0.0008191 | 27 | 21 | 1.15 |  |  |
| FBgn0040726 | dpr | 0.00653483 | 27 | 23 | 1.25 |  |  |
| FBgn0040734 | CG15065 | 0.01520292 | 21 | 7 | 0.74 |  |  |
| FBgn0040843 | CG15213 | 0.00877216 | 27 | 25 | 1.16 | yes |  |
| FBgn0040859 | CR32658 | 0.00507239 | 20 | 9 | 2.30 |  |  |
| FBgn0040907 | mRpL33 | 0.02052367 | 28 | 26 | 1.39 | yes |  |
| FBgn0040928 | CG15345 | 0.00177493 | 22 | 17 | 1.17 | yes |  |
| FBgn0040929 | CG12659 | 0.00293729 | 21 | 18 | 0.84 |  |  |
| FBgn0040963 | CG18662 | 0.00839135 | 22 | 7 | 1.35 |  |  |
| FBgn0040996 | CG12617 | 0.02017946 | 21 | 7 | 1.55 |  |  |
| FBgn0041092 | tai | 0.01571532 | 21 | 17 | 0.50 |  |  |
| FBgn0041096 | rols | 0.00437803 | 24 | 16 | 0.70 |  |  |
| FBgn0041102 | ocn | 0.00200915 | 21 | 8 | 2.09 |  |  |
| FBgn0041711 | yellow-e | 0.01294918 | 22 | 18 | 0.67 |  |  |
| FBgn0041713 | yellow-c | 0.01558019 | 22 | 18 | 0.87 |  |  |
| FBgn0042105 | CG18748 | 0.00341603 | 24 | 8 | 1.01 |  |  |
| FBgn0042111 | CG18766 | 0.01173786 | 21 | 18 | 0.58 |  |  |
| FBgn0042206 | GstD10 | 0.00545843 | 26 | 25 | 0.96 |  |  |
| FBgn0042627 | v(2)k05816 | 0.00567908 | 24 | 10 | 1.85 |  |  |
| FBgn0042630 | Sox21b | 0.00414703 | 20 | 18 | 1.12 |  |  |
| FBgn0042650 | disco-r | 0.02050208 | 20 | 18 | 0.64 |  |  |
| FBgn0043043 | desat2 | 0.00292525 | 24 | 19 | 1.43 |  |  |
| FBgn0043530 | Obp51a | 0.02088663 | 23 | 6 | 1.64 |  |  |
| FBgn0043783 | CG32444 | 0.01934525 | 24 | 13 | 0.78 |  |  |
| FBgn0043854 | slam | 0.01304322 | 21 | 19 | 0.70 |  |  |
| FBgn0044511 | mRpS21 | 0.01185577 | 26 | 2 | 0.50 | yes |  |
| FBgn0045202 | CG33992 | 0.00123496 | 21 | 8 | 1.59 |  |  |
| FBgn0045770 | S-Lap3 | 0.00332461 | 20 | 8 | 1.96 |  |  |
| FBgn0046294 | CG12699 | 0.01938913 | 22 | 7 | 2.30 |  |  |
| FBgn0046297 | CG9284 | 0.00037096 | 20 | 8 | 2.18 |  |  |
| FBgn0046332 | gskt | 0.005905 | 20 | 8 | 1.31 |  |  |
| FBgn0046685 | Wsck | 0.00223966 | 22 | 20 | 0.55 | yes |  |
| FBgn0046793 | CG32236 | 0.00039158 | 20 | 8 | 1.52 |  |  |
| FBgn0047038 | CG6463 | 0.00897239 | 26 | 1 | 0.58 |  |  |
| FBgn0047351 | CG31468 | 0.01295173 | 22 | 7 | 1.72 |  |  |
| FBgn0050033 | CG30033 | 0.01605717 | 20 | 20 | 0.97 |  |  |
| FBgn0050044 | s-cup | 0.00536195 | 24 | 20 | 0.61 | yes |  |
| FBgn0050096 | CG30096 | 0.00234316 | 21 | 19 | 1.21 |  |  |
| FBgn0050103 | CG30103 | 0.00136778 | 21 | 9 | 2.37 |  |  |
| FBgn0050105 | CG30105 | 0.01582833 | 20 | 19 | 1.18 |  |  |
| FBgn0050156 | CG30156 | 0.00323089 | 21 | 8 | 1.72 |  |  |
| FBgn0050177 | CG30177 | 0.01673434 | 20 | 9 | 2.30 |  |  |
| FBgn0050181 | CG30181 | 0.01533273 | 22 | 20 | 1.56 |  |  |
| FBgn0050203 | CG30203 | 0.0182863 | 24 | 15 | 0.81 |  |  |
| FBgn0050268 | CG30268 | 0.00696831 | 20 | 8 | 3.89 |  |  |
| FBgn0050293 | Cht12 | 0.01445862 | 21 | 7 | 2.74 |  |  |
| FBgn0050338 | CG30338 | 0.01158947 | 20 | 18 | 0.86 |  |  |
| FBgn0050350 | CG30350 | 0.00917826 | 21 | 8 | 2.00 |  |  |
| FBgn0050357 | CG30357 | 0.00324976 | 27 | 11 | 1.59 |  |  |
| FBgn0050360 | Mal-A6 | 0.00056545 | 21 | 9 | 2.56 |  |  |
| FBgn0050361 | mtt | 0.00158435 | 24 | 17 | 1.17 | yes |  |
| FBgn0050365 | spaw | 0.01375755 | 21 | 8 | 1.78 |  |  |
| FBgn0050381 | CG30381 | 0.0029044 | 20 | 19 | 0.97 |  |  |
| FBgn0050390 | Sgf29 | 0.01311864 | 27 | 26 | 0.70 |  |  |
| FBgn0050403 | CG30403 | 0.00703215 | 21 | 18 | 1.29 |  |  |
| FBgn0050414 | CG30414 | 0.00299813 | 21 | 18 | 0.73 |  |  |
| FBgn0050463 | CG30463 | 0.01604913 | 28 | 15 | 0.77 | yes |  |
| FBgn0051010 | CG31010 | 0.00677696 | 21 | 8 | 1.71 |  |  |
| FBgn0051025 | CG31025 | 0.00742287 | 22 | 7 | 1.38 |  |  |
| FBgn0051029 | CG31029 | 0.00041177 | 20 | 8 | 1.40 | yes |  |
| FBgn0051032 | CR31032 | 0.00162 | 22 | 22 | 0.52 |  |  |
| FBgn0051055 | CG31055 | 0.00071094 | 22 | 7 | 1.51 |  |  |
| FBgn0051100 | CG31100 | 0.00721515 | 24 | 15 | 0.77 |  |  |
| FBgn0051104 | CG31104 | 0.00691348 | 20 | 10 | 0.96 |  |  |
| FBgn0051157 | CG31157 | 0.01436281 | 26 | 8 | 1.13 |  |  |
| FBgn0051198 | CG31198 | 0.00768527 | 20 | 10 | 1.65 |  |  |
| FBgn0051204 | CG31204 | 0.01080153 | 21 | 8 | 2.03 |  |  |
| FBgn0051205 | CG31205 | 0.01151443 | 20 | 2 | 0.64 |  |  |
| FBgn0051223 | CG31223 | 0.00669467 | 21 | 20 | 0.58 |  |  |
| FBgn0051226 | CG31226 | 0.00085168 | 20 | 8 | 1.85 |  |  |
| FBgn0051231 | CG31231 | 0.00140406 | 21 | 7 | 1.63 |  |  |
| FBgn0051244 | CG31244 | 0.00529629 | 21 | 7 | 1.70 |  |  |
| FBgn0051324 | CG31324 | 0.01217843 | 21 | 17 | 1.18 |  |  |
| FBgn0051327 | CG31327 | 0.00105078 | 21 | 8 | 1.35 |  |  |
| FBgn0051347 | CG31347 | 0.01539028 | 20 | 8 | 1.07 |  |  |
| FBgn0051361 | dpr17 | 0.00103343 | 20 | 9 | 0.63 | yes |  |
| FBgn0051365 | CG31365 | 0.01307903 | 21 | 18 | 0.80 |  |  |
| FBgn0051391 | CG31391 | 0.00842886 | 20 | 8 | 3.43 |  |  |
| FBgn0051459 | CG31459 | 0.01171006 | 21 | 7 | 2.81 |  |  |
| FBgn0051481 | pb | 0.00489843 | 20 | 18 | 1.11 |  |  |
| FBgn0051482 | CG31482 | 0.00500961 | 21 | 8 | 1.84 |  |  |
| FBgn0051635 | CG31635 | 0.00075791 | 24 | 18 | 0.70 |  |  |
| FBgn0051639 | CG31639 | 0.01136028 | 20 | 8 | 1.91 |  |  |
| FBgn0051675 | CG31675 | 0.00304231 | 27 | 23 | 0.54 | yes |  |
| FBgn0051693 | CG31693 | 0.00913594 | 21 | 8 | 3.70 |  |  |
| FBgn0051709 | CG31709 | 0.00523282 | 20 | 8 | 1.25 |  |  |
| FBgn0051719 | RluA-1 | 0.01759884 | 24 | 16 | 0.91 | yes |  |
| FBgn0051730 | CG31730 | 0.00047723 | 22 | 6 | 1.96 |  |  |
| FBgn0051773 | CG31773 | 0.00948335 | 21 | 8 | 1.94 |  |  |
| FBgn0051782 | CG31782 | 0.01635955 | 21 | 18 | 0.78 |  |  |
| FBgn0051788 | CG31788 | 0.00724598 | 21 | 8 | 1.55 |  |  |
| FBgn0051798 | CG31798 | 0.00089823 | 20 | 8 | 1.69 |  |  |
| FBgn0051816 | CG31816 | 0.00796081 | 21 | 9 | 2.86 |  |  |
| FBgn0051849 | CG31849 | 0.00576752 | 21 | 19 | 1.04 | yes |  |
| FBgn0051869 | CG31869 | 0.00517826 | 22 | 20 | 0.52 |  |  |
| FBgn0051870 | CG31870 | 0.00866997 | 20 | 8 | 2.01 |  |  |
| FBgn0051874 | CG31874 | 0.0052597 | 21 | 7 | 2.13 |  |  |
| FBgn0051921 | CG31921 | 0.0054239 | 21 | 7 | 1.88 |  |  |
| FBgn0051949 | CG31949 | 0.00977236 | 22 | 6 | 1.97 |  |  |
| FBgn0052026 | CG32026 | 0.00719168 | 21 | 8 | 1.32 |  |  |
| FBgn0052057 | dpr10 | 0.01703094 | 22 | 18 | 0.72 | yes |  |
| FBgn0052064 | S-Lap4 | 0.00200635 | 20 | 8 | 1.94 |  |  |
| FBgn0052081 | CG32081 | 0.01421418 | 21 | 7 | 2.26 |  |  |
| FBgn0052095 | CG32095 | 0.01862811 | 21 | 2 | 0.54 |  |  |
| FBgn0052111 | CG32111 | 0.00523192 | 22 | 17 | 0.74 |  |  |
| FBgn0052119 | CG32119 | 0.00848592 | 23 | 7 | 1.34 |  |  |
| FBgn0052181 | CG32181 | 0.01163042 | 20 | 8 | 3.18 |  |  |
| FBgn0052351 | S-Lap2 | 0.00137145 | 20 | 8 | 2.12 |  |  |
| FBgn0052371 | CG32371 | 0.00098584 | 20 | 8 | 1.47 |  |  |
| FBgn0052407 | CG32407 | 0.01438549 | 24 | 3 | 0.58 | yes |  |
| FBgn0052436 | CG32436 | 0.00135106 | 20 | 8 | 3.11 | yes |  |
| FBgn0052437 | CG32437 | 0.0157251 | 21 | 8 | 2.30 |  |  |
| FBgn0052479 | CG32479 | 0.01166295 | 21 | 8 | 1.55 | yes | yes |
| FBgn0052487 | CG32487 | 0.00761846 | 20 | 8 | 3.10 |  |  |
| FBgn0052537 | CG32537 | 0.00261087 | 20 | 19 | 0.75 | yes |  |
| FBgn0052582 | CG32582 | 0.0005779 | 25 | 23 | 0.61 |  |  |
| FBgn0052643 | CG32643 | 0.00337719 | 23 | 7 | 1.08 |  |  |
| FBgn0052652 | CG32652 | 0.00132734 | 20 | 9 | 2.29 |  |  |
| FBgn0052686 | CG32686 | 0.00054659 | 20 | 8 | 1.37 |  |  |
| FBgn0052690 | CR32690 | 0.00435669 | 20 | 9 | 1.78 |  |  |
| FBgn0052703 | CG32703 | 0.00223963 | 21 | 8 | 2.36 |  |  |
| FBgn0052718 | CG32718 | 0.00361135 | 21 | 9 | 1.74 |  |  |
| FBgn0052855 | CG32855 | 0.0111363 | 23 | 20 | 1.17 |  |  |
| FBgn0053007 | CG33007 | 0.01160029 | 21 | 19 | 0.93 |  |  |
| FBgn0053093 | CG33093 | 0.00367538 | 26 | 13 | 2.76 |  |  |
| FBgn0053096 | CG33096 | 0.0164851 | 20 | 20 | 0.67 |  |  |
| FBgn0053145 | CG33145 | 0.00054682 | 21 | 18 | 0.63 |  |  |
| FBgn0053181 | CG33181 | 0.00230184 | 21 | 21 | 0.73 | yes |  |
| FBgn0053208 | Mical | 0.01518775 | 26 | 14 | 0.97 |  |  |
| FBgn0053213 | CG33213 | 0.01074546 | 21 | 18 | 0.54 |  |  |
| FBgn0053284 | CG33284 | 0.00048497 | 20 | 8 | 2.04 |  |  |
| FBgn0053286 | CG33286 | 0.00085246 | 20 | 9 | 1.72 |  |  |
| FBgn0053287 | CG33287 | 0.00603422 | 21 | 8 | 1.42 |  |  |
| FBgn0053290 | CG33290 | 0.01225605 | 27 | 2 | 3.19 |  |  |
| FBgn0053296 | CG33296 | 0.0007101 | 28 | 14 | 1.12 |  |  |
| FBgn0053340 | CG33340 | 0.00659947 | 21 | 8 | 1.63 |  |  |
| FBgn0053510 | CG33510 | 0.00785001 | 22 | 20 | 1.28 |  |  |
| FBgn0054001 | CG34001 | 0.01789696 | 20 | 19 | 1.46 |  |  |
| FBgn0054026 | CG34026 | 0.00389733 | 22 | 8 | 1.89 |  |  |
| FBgn0054034 | CG34034 | 0.01496199 | 24 | 5 | 1.81 |  |  |
| FBgn0061197 | SIP2 | 0.00616599 | 21 | 7 | 1.75 |  |  |
| FBgn0062449 | CG13197 | 0.00623295 | 20 | 8 | 0.71 |  |  |
| FBgn0063368 | Gpb5 | 0.00958355 | 21 | 20 | 0.74 | yes |  |
| FBgn0067629 | CG33332 | 0.00258504 | 21 | 20 | 0.80 |  |  |
| FBgn0083015 | snoRNA:Psi18S-920 | 0.01406672 | 20 | 19 | 1.35 |  |  |
| FBgn0083077 | mld | 0.01236202 | 28 | 15 | 0.55 | yes |  |
| FBgn0083946 | CG34110 | 0.01061082 | 21 | 8 | 1.70 |  |  |
| FBgn0083949 | CG34113 | 0.01329345 | 26 | 15 | 0.72 |  |  |
| FBgn0085193 | CG34164 | 0.00158985 | 21 | 19 | 0.93 | yes |  |
| FBgn0085203 | CG34174 | 0.01734609 | 20 | 19 | 1.10 |  |  |
| FBgn0085209 | CG34180 | 0.00123372 | 26 | 24 | 1.30 |  |  |
| FBgn0085223 | CG34194 | 0.01858501 | 24 | 5 | 1.89 |  |  |
| FBgn0085279 | CG34250 | 0.01750463 | 24 | 3 | 0.58 |  |  |
| FBgn0085282 | CG34253 | 0.00236521 | 21 | 19 | 1.11 | yes |  |
| FBgn0085329 | CG34300 | 0.0205422 | 22 | 6 | 3.01 |  |  |
| FBgn0085380 | CG34351 | 0.00466321 | 22 | 16 | 0.55 |  |  |
| FBgn0085382 | CG34353 | 0.00185363 | 23 | 18 | 0.87 | yes |  |
| FBgn0085384 | CG34355 | 0.00962674 | 21 | 18 | 0.76 |  |  |
| FBgn0085386 | CG34357 | 0.01150805 | 23 | 18 | 1.35 | yes |  |
| FBgn0085404 | CG34375 | 0.02027875 | 22 | 18 | 1.00 |  |  |
| FBgn0085431 | CG34402 | 0.02039719 | 20 | 18 | 1.14 |  |  |
| FBgn0085434 | NaCP60E | 0.0175526 | 24 | 17 | 0.99 |  |  |
| FBgn0085454 | CG34425 | 0.00344248 | 21 | 8 | 1.46 |  |  |
| FBgn0085479 | CG34450 | 0.00456916 | 20 | 8 | 2.42 |  |  |
| FBgn0085481 | CG34452 | 0.01346446 | 27 | 20 | 1.18 |  |  |
| FBgn0085732 | CR40190 | 0.00891561 | 20 | 15 | 0.78 |  |  |
| FBgn0085786 | CR41501 | 0.00639915 | 20 | 18 | 1.05 |  |  |
| FBgn0085793 | CR41510 | 0.00497071 | 21 | 18 | 0.94 |  |  |
| FBgn0086365 | Orct2 | 0.00685875 | 21 | 19 | 0.80 |  |  |
| FBgn0086370 | sra | 0.00662161 | 26 | 12 | 0.76 |  |  |
| FBgn0086448 | Gpi1 | 0.01880469 | 20 | 17 | 0.78 | yes |  |
| FBgn0086655 | jing | 0.01007147 | 24 | 18 | 0.90 |  |  |
| FBgn0086681 | Mst36Fa | 0.01005915 | 20 | 8 | 1.97 |  |  |
| FBgn0086915 | Mst77F | 0.01281381 | 21 | 7 | 2.08 | yes |  |
| FBgn0087007 | bbg | 0.00829403 | 23 | 20 | 0.62 | yes |  |
| FBgn0243486 | rdo | 0.01314228 | 26 | 14 | 0.62 | yes |  |
| FBgn0243512 | puc | 0.01126811 | 20 | 13 | 0.60 | yes |  |
| FBgn0250755 | CG42233 | 0.01093182 | 20 | 18 | 0.97 |  |  |
| FBgn0250821 | CG14644 | 0.00478844 | 20 | 8 | 1.70 |  |  |
| FBgn0250841 | CG17242 | 0.00760314 | 22 | 8 | 1.71 |  |  |
| FBgn0250845 | CG1288 | 0.01878857 | 21 | 7 | 2.52 |  |  |
| FBgn0250849 | CG32388 | 0.01843533 | 22 | 7 | 1.50 |  |  |
| FBgn0250868 | CG42239 | 0.00415495 | 20 | 18 | 1.16 |  |  |
| FBgn0259108 | futsch | 0.01398389 | 24 | 17 | 0.99 |  |  |
| FBgn0259109 | CG42251 | 0.00376386 | 21 | 18 | 1.19 |  |  |
| FBgn0259164 | CG42269 | 0.00487593 | 23 | 19 | 2.11 |  |  |
| FBgn0259167 | CG42272 | 0.01654458 | 23 | 19 | 1.14 |  |  |
| FBgn0259171 | Pde9 | 0.01485616 | 22 | 18 | 0.72 |  |  |
| FBgn0259222 | CG42322 | 0.0011977 | 27 | 14 | 0.82 | yes |  |
| FBgn0259701 | CG42355 | 0.01672882 | 21 | 8 | 2.71 |  |  |
| FBgn0259734 | CG42388 | 0.01079049 | 22 | 18 | 1.02 | yes |  |
| FBgn0259795 | loopin-1 | 0.00263236 | 21 | 8 | 1.52 |  |  |
| FBgn0259823 | CG42404 | 0.00214752 | 20 | 18 | 0.97 |  |  |
| FBgn0259896 | nimC1 | 0.00497775 | 21 | 20 | 1.04 |  |  |
| FBgn0260386 | mtg | 0.00256574 | 26 | 17 | 1.08 |  |  |
| FBgn0260429 | CG42524 | 0.00062284 | 21 | 19 | 0.88 |  |  |
| FBgn0260480 | CG32295 | 0.01839841 | 20 | 8 | 1.24 |  |  |
| FBgn0260648 | Rrp40 | 0.00166683 | 20 | 20 | 0.78 |  |  |
| FBgn0260655 | l(3)76BDm | 0.00127947 | 26 | 13 | 0.62 |  |  |
| FBgn0260742 | CG12213 | 0.01718616 | 22 | 17 | 0.80 |  |  |
| FBgn0260749 | Utx | 0.00976625 | 27 | 14 | 0.58 |  |  |
| FBgn0260764 | CG42562 | 0.00376958 | 21 | 19 | 0.75 |  |  |
| FBgn0260776 | CG42570 | 0.01028642 | 20 | 8 | 3.46 |  |  |
| FBgn0260789 | mxc | 0.00421 | 23 | 19 | 0.69 |  |  |
| FBgn0260862 | Vti1 | 0.00430538 | 20 | 19 | 0.76 |  |  |
| FBgn0260959 | MCPH1 | 0.00236929 | 21 | 7 | 0.55 |  |  |
| FBgn0261016 | clos | 0.0077847 | 25 | 14 | 0.96 |  |  |
| FBgn0261046 | Dscam3 | 0.00997335 | 25 | 18 | 1.24 | yes |  |
| FBgn0261112 | APP-BP1 | 0.00121491 | 20 | 9 | 0.50 |  |  |
| FBgn0261287 | ymp | 0.00550647 | 20 | 9 | 1.65 |  |  |
| FBgn0261349 | Mst36Fb | 0.02047909 | 20 | 9 | 1.84 |  |  |
| FBgn0261380 | mRpL37 | 0.01112753 | 21 | 20 | 0.56 |  |  |
| FBgn0261385 | scra | 0.01061382 | 21 | 19 | 0.59 |  |  |
| FBgn0261397 | didum | 0.01123449 | 26 | 13 | 1.00 |  |  |
| FBgn0261508 | CG42656 | 0.00705371 | 28 | 26 | 1.91 |  |  |
| FBgn0261529 | ms(2)34Fe | 0.01210949 | 21 | 8 | 2.61 |  |  |
| FBgn0261545 | CG42663 | 0.01728278 | 23 | 15 | 0.73 |  |  |
| FBgn0261553 | CG42671 | 0.00203886 | 20 | 19 | 0.56 |  |  |
| FBgn0261612 | Cng | 0.01665849 | 24 | 19 | 1.42 |  |  |
| FBgn0261714 | Cpn | 0.00602162 | 27 | 12 | 0.53 | yes |  |
| FBgn0261791 | SmG | 0.00787904 | 28 | 26 | 1.07 |  |  |
| FBgn0261793 | Trf2 | 0.01574469 | 23 | 17 | 0.67 |  |  |
| FBgn0261811 | pico | 0.00748161 | 22 | 16 | 0.57 |  |  |
| FBgn0261835 | CR42767 | 0.00447019 | 20 | 9 | 1.72 |  |  |
| FBgn0261859 | CG42788 | 0.01954879 | 23 | 17 | 0.69 |  |  |
| FBgn0261975 | CG42806 | 0.00739622 | 21 | 20 | 0.62 |  |  |
| FBgn0261999 | CG42817 | 0.00200605 | 27 | 21 | 0.65 |  |  |
| FBgn0262002 | CG42820 | 0.00078547 | 20 | 8 | 1.55 |  |  |
| FBgn0262103 | CG42856 | 0.02069977 | 21 | 17 | 0.76 |  |  |
| FBgn0262539 | CG43093 | 0.01781339 | 24 | 1 | 0.97 |  |  |
| FBgn0262547 | CG43101 | 0.02024896 | 25 | 4 | 2.12 |  |  |
| FBgn0262573 | orb2 | 0.00096713 | 20 | 9 | 0.78 | yes | yes |
| FBgn0262574 | CG43114 | 0.01974943 | 27 | 0 | 0.87 |  |  |
| FBgn0262581 | CG43121 | 0.00646121 | 24 | 7 | 1.21 |  |  |
| FBgn0262729 | CG31369 | 0.01667642 | 21 | 6 | 0.96 |  |  |
| FBgn0262738 | norpA | 0.00704207 | 27 | 11 | 0.91 | yes | yes |
| FBgn0262743 | Fs(2)Ket | 0.01153233 | 22 | 11 | 0.66 |  | yes |
| FBgn0262993 | CR43301 | 0.00041806 | 21 | 18 | 0.61 |  |  |
| FBgn0263048 | CG43343 | 0.00495611 | 20 | 8 | 1.12 |  |  |
| FBgn0263076 | Klp54D | 0.00445746 | 21 | 15 | 1.09 | yes |  |
| FBgn0263111 | cac | 0.0072267 | 28 | 17 | 1.50 |  |  |
| FBgn0263237 | Cdk7 | 0.00841627 | 22 | 19 | 0.54 |  |  |
| *FBgn0263034* | *CG43329* | *0.00090707* | *24* | *8* | *15.11* |  |  |
| *FBgn0029817* | *CG15764* | *0.00227329* | *24* | *6* | *5.58* |  |  |
| *FBgn0050222* | *CG30222* | *0.00227329* | *24* | *8* | *3.99* |  |  |
| *FBgn0038775* | *CG17199* | *0.0032227* | *24* | *6* | *2.60* |  |  |
| *FBgn0036482* | *CG13457* | *0.0032227* | *24* | *6* | *2.99* |  |  |
| *FBgn0029703* | *CG12692* | *0.0032227* | *24* | *6* | *6.68* |  |  |
| *FBgn0084008* | *CR41443* | *0.00521172* | *24* | *6* | *6.88* |  |  |
| *FBgn0036029* | *CG16719* | *0.00720073* | *24* | *6* | *4.54* |  |  |
| *FBgn0029940* | *CG1958* | *0.00720073* | *24* | *6* | *4.08* |  |  |
| *FBgn0036204* | *Tim13* | *0.00720073* | *20* | *10* | *2.77* |  |  |
| *FBgn0036497* | *ran-like* | *0.00720073* | *24* | *6* | *4.15* |  |  |
| *FBgn0036443* | *CG13471* | *0.00720073* | *24* | *8* | *2.03* |  |  |
| *FBgn0030449* | *Fer3HCH* | *0.00720073* | *20* | *8* | *7.16* |  |  |
| *FBgn0035005* | *CG3483* | *0.00720073* | *24* | *6* | *2.07* |  |  |
| *FBgn0036688* | *Fit2* | *0.00720073* | *24* | *20* | *0.47* |  |  |
| *FBgn0028986* | *Spn3* | *0.00720073* | *24* | *8* | *2.51* |  |  |
| *FBgn0033248* | *Dic3* | *0.00720073* | *24* | *6* | *3.78* |  |  |
| *FBgn0030846* | *CG12992* | *0.00720073* | *24* | *6* | *8.00* |  |  |
| *FBgn0003174* | *pwn* | *0.00720073* | *28* | *16* | *1.27* |  |  |
| *FBgn0035217* | *FucTD* | *0.00720073* | *24* | *6* | *4.05* |  |  |
| *FBgn0038565* | *CG7794* | *0.00720073* | *24* | *6* | *1.66* |  |  |
| *FBgn0015577* | *alpha-Est9* | *0.00720073* | *20* | *0* | *0.48* |  |  |
| *FBgn0039331* | *CG11913* | *0.00720073* | *24* | *6* | *2.70* |  |  |
| *FBgn0032419* | *CG17217* | *0.00720073* | *24* | *7* | *2.90* |  |  |
| *FBgn0262487* | *CG43077* | *0.01108362* | *24* | *6* | *4.55* |  |  |
| *FBgn0262351* | *CG43049* | *0.01108362* | *24* | *6* | *1.78* |  |  |
| *FBgn0031831* | *CG11043* | *0.01108362* | *24* | *6* | *2.10* |  |  |
| *FBgn0031163* | *CG14579* | *0.01108362* | *24* | *6* | *8.19* |  |  |
| *FBgn0052086* | *CG32086* | *0.01108362* | *24* | *6* | *7.20* |  |  |
| *FBgn0030374* | *CG15734* | *0.01108362* | *24* | *6* | *5.53* |  |  |
| *FBgn0250844* | *CG4218* | *0.01108362* | *24* | *6* | *4.23* |  |  |
| *FBgn0031235* | *CG13693* | *0.01496651* | *24* | *7* | *5.24* |  |  |
| *FBgn0031130* | *CG15452* | *0.01496651* | *24* | *6* | *2.08* |  |  |
| *FBgn0013278* | *Hsp70Bb* | *0.01496651* | *20* | *0* | *1.24* |  |  |
| *FBgn0031585* | *CG2955* | *0.01496651* | *24* | *6* | *3.28* |  |  |
| *FBgn0030077* | *CG15365* | *0.01496651* | *24* | *18* | *1.32* |  |  |
| *FBgn0032313* | *CG14070* | *0.01496651* | *24* | *6* | *7.21* |  |  |
| *FBgn0028901* | *CG18109* | *0.01496651* | *24* | *8* | *2.51* |  |  |
| *FBgn0003250* | *Rh4* | *0.01496651* | *20* | *2* | *0.69* |  |  |
| *FBgn0031878* | *sip2* | *0.01496651* | *24* | *6* | *1.58* |  |  |
| *FBgn0031946* | *CG7164* | *0.01496651* | *24* | *7* | *4.99* |  |  |
| *FBgn0038321* | *CG6218* | *0.01496651* | *24* | *2* | *0.53* | *yes* |  |
| *FBgn0050362* | *boly* | *0.01496651* | *24* | *8* | *2.53* |  |  |
| *FBgn0051820* | *CG31820* | *0.01496651* | *24* | *6* | *4.22* |  |  |
| *FBgn0035481* | *CG12605* | *0.01496651* | *24* | *6* | *2.62* | *yes* |  |
| *FBgn0038475* | *Keap1* | *0.01496651* | *24* | *18* | *0.61* |  |  |
| *FBgn0261806* | *CG42752* | *0.01496651* | *24* | *6* | *7.67* |  |  |
| *FBgn0051624* | *CG31624* | *0.01496651* | *24* | *8* | *6.81* |  |  |
| *FBgn0085376* | *CG34347* | *0.01496651* | *24* | *18* | *1.43* | *yes* |  |
| *FBgn0083943* | *CG34107* | *0.01496651* | *28* | *4* | *10.06* |  |  |
| *FBgn0053191* | *CG33191* | *0.01496651* | *24* | *6* | *2.96* |  |  |
| *FBgn0085400* | *CG34371* | *0.01496651* | *24* | *18* | *0.66* |  |  |
| *FBgn0033168* | *CG11145* | *0.01496651* | *24* | *6* | *7.19* |  |  |
| *FBgn0034601* | *CG4286* | *0.01496651* | *24* | *8* | *4.44* |  |  |
| *FBgn0033063* | *CG14589* | *0.01496651* | *24* | *6* | *2.37* |  |  |
| *FBgn0032625* | *CG15136* | *0.01496651* | *24* | *6* | *1.42* |  |  |
| *FBgn0036837* | *CG18135* | *0.01496651* | *24* | *18* | *0.52* | *yes* |  |
| *FBgn0034464* | *CG11018* | *0.01496651* | *24* | *6* | *2.94* |  |  |
| *FBgn0034592* | *CG9406* | *0.01496651* | *20* | *10* | *11.22* |  |  |
| *FBgn0035371* | *CG9977* | *0.01496651* | *24* | *20* | *0.83* |  |  |

Note: The first 203 cycling mRNAs (shown in bold font) were identified by both ARSER and JTK_CYCLE. Next in the table are mRNAs identified by ARSER alone (in regular font), followed by those identified by JTK_CYCLE alone (in italic font).
