# Supplementary material for: Translational Profiling of Clock Cells Reveals Circadianly Synchronized Protein Synthesis
Source: PLoS Biol. 2013 Nov 5;11(11):e1001703. doi: 10.1371/journal.pbio.1001703 (PMC3864454; doi:10.1371/journal.pbio.1001703)
Supplement: Table S5 — Primers used in the Q-RT-PCR experiments. (DOCX) [file pbio.1001703.s014.docx]

Table S5. Primers used in the Q-RT-PCR experiments.

| **Primer Name** | **Sequence** |
| --- | --- |
| Rp49-F | GCCCAAGATCGTGAAGAAGC |
| Rp49-R | CGACGCACTCTGTTGTCG |
| inaF-B-F1 | ACGCATCCGATTGGCTAGGA |
| inaF-B-R1 | AACTGCTCCGCGGTCACTTC |
| CG17237-F2 | GTACGAGGCCTCCACCATGC |
| CG17237-R2 | CTGGTCCGCCTCGATCTGTT |
| Trxr-2-F2 | GTCCGGGGACCAGAAGATCC |
| Trxr-2-R2 | GGTGGATTCCCACCGTGTTC |
| TrxT-F1 | CGTGGACGAGAACGAGGACA |
| TrxT-R1 | TAAACGCCGGCATGCTTCTC |
| ACXC-F2 | ATATGGCGGCCTGTGGATTG |
| ACXC-R2 | CGTGCGTGTTCCACCTCAAC |
| CG8170-F2 | GCGTCAGCAAGACCGTGGAC |
| CG8170-R2 | GGCCCACTTAGCAGGCAGGT |
| Ubpy-F1 | ATCGAAGCTGCCACCTGTGC |
| Ubpy-R1 | GCGGGAAGCGCAAGTAGTTC |
| CG8888-F2 | TCGCCGTGTTCGTCTGGTT |
| CG8888-R2 | GGCCAAGTACCAGGCCAGTG |
| Ugt35b-2F | GACACCGCCATTTGGTGGAC |
| Ugt35b-2R | CCAGGCTGTGGTAGGCGAAG |
| Ugt35b-3F | TTATCACCCACGGCGGATTG |
| Ugt35b-3R | GGCCCGCCTTACATTCAGGA |
| CG17199-F2 | GCCTTTCAGCCGGAGCATAA |
| CG17199-R2 | GCCACGACGCTGGATTTGAT |
| CG9977-F1 | CTCATCGAGACGCTGGTGGA |
| CG9977-R1 | GCCAGGCAAAGATCGGGATT |
| Tdc2-F1 | CCAACAAATGGCTGCTGACG |
| Tdc2-R1 | GCATCCGAGTAGCCGTGCTT |
| Tdc2-4F | GCACGATGAGCTGTCCGAGA |
| Tdc2-4R | CTTGTTGATCGCCGGGTTGT |
| eIF4E-4-F1 | GACGACCCAGCAGACCGAAT |
| eIF4E-4-R1 | ACGGGCTTGCGAATATCCAT |
| eIF4E-3-2F | GCTGCATCTGGGCATTATGGA |
| eIF4E-3-2R | TATGGCGCCCTTGGAAATTG |
| eIF4E-5-F1 | CCCATGTGGGAGGATGAAGC |
| eIF4E-5-R1 | TGGCCCACCATCAGCAGTAG |
| eIF4E-7-F1 | GAGGACGCTGGGTCATCAAC |
| eIF4E-7-R1 | TTGACCACAACGCCACAAAG |
| eIF4E-6-F1 | CACCGGCTGCAGAACACAT |
| eIF4E-6-R1 | GGCGTATCGATGCGGAAATA |
| syt12-F1 | TGCAGAGGGACAGTGCGTTTC |
| syt12-R1 | TCGAACTTACCGCAAAAAGTTGTCA |
| CG13091-F1 | CGGAATGCGGGTGTTCCTTT |
| CG13091-R1 | CCACACAATCTTTCCGAGAGCAAG |
| CG13091-F2 | TGCCTTGCTCTCGGAAAGATTGT |
| CG13091-R2 | GAAAATCGGCCACCGGAGA |
| CG13725-F1 | CTCGCACTGTGGTGCTGGAC |
| CG13725-R1 | GGTCTACGGACGCCAACAGG |
| CG13725-F2 | TCGCACTGTGGTGCTGGACTA |
| CG13725-R2 | GGACGCCAACAGGTTCATCAC |
| CG18107-F | TCGTTTGTCCGGTGTGTTCAG |
| CG18107-R | GGCCAAAGCCAGAAGACCAA |
| POF-F | TGGTCGACTCACCGCTGAAC |
| POF-R | CTGAAAGGAGGTGGCGCACT |
| CG17261-F | GGAGCACGTCAGAGCCTTCG |
| CG17261-R | TGCGGACCGTCTTGGTCAT |
